# Global transcriptome analysis of two *ameiotic1 *alleles in maize anthers: defining steps in meiotic entry and progression through prophase I

**DOI:** 10.1186/1471-2229-11-120

**Published:** 2011-08-26

**Authors:** Guo-Ling Nan, Arnaud Ronceret, Rachel C Wang, John F Fernandes, W Zacheus Cande, Virginia Walbot

**Affiliations:** 1 Department of Biology, Stanford University, Stanford, CA 94305, USA; 2 Department of Molecular and Cell Biology, University of California, Berkeley, CA 94720, USA; 3 Institute of Plant and Microbial Biology (IPMB), Academia Sinica, Taipei, 11529, Taiwan

**Keywords:** meiosis, meiocytes, transcriptomes, leptotene/zygotene transition, pollen mother cells

## Abstract

**Background:**

Developmental cues to start meiosis occur late in plants. *Ameiotic1 *(*Am1*) encodes a plant-specific nuclear protein (AM1) required for meiotic entry and progression through early prophase I. Pollen mother cells (PMCs) remain mitotic in most *am1 *mutants including *am1-489*, while *am1-praI *permits meiotic entry but PMCs arrest at the leptotene/zygotene (L/Z) transition, defining the roles of AM1 protein in two distinct steps of meiosis. To gain more insights into the roles of AM1 in the transcriptional pre-meiotic and meiotic programs, we report here an in depth analysis of gene expression alterations in carefully staged anthers at 1 mm (meiotic entry) and 1.5 mm (L/Z) caused by each of these *am1 *alleles.

**Results:**

1.0 mm and 1.5 mm anthers of *am1-489 *and *am1-praI *were profiled in comparison to fertile siblings on Agilent^® ^4 × 44 K microarrays. Both *am1-489 *and *am1-praI *anthers are cytologically normal at 1.0 mm and show moderate transcriptome alterations. At the 1.5-mm stage both mutants are aberrant cytologically, and show more drastic transcriptome changes. There are substantially more absolute On/Off and twice as many differentially expressed genes (sterile versus fertile) in *am1-489 *than in *am1-praI*. At 1.5 mm a total of 4,418 genes are up- or down-regulated in either *am1-489 *or *am1-praI *anthers. These are predominantly stage-specific transcripts. Many putative meiosis-related genes were found among them including a small subset of allele-specific, mis-regulated genes specific to the PMCs. Nearly 60% of transcriptome changes in the set of transcripts mis-regulated in both mutants (N = 530) are enriched in PMCs, and only 1% are enriched in the tapetal cell transcriptome. All array data reported herein will be deposited and accessible at MaizeGDB http://www.maizegdb.org/.

**Conclusions:**

Our analysis of anther transcriptome modulations by two distinct *am1 *alleles, *am1-489 *and *am1-praI*, redefines the role of AM1 as a modulator of expression of a subset of meiotic genes, important for meiotic progression and provided stage-specific insights into the genetic networks associated with meiotic entry and early prophase I progression.

## Background

During sexual reproduction meiosis insures that progeny receive half their genetic information from each parent, thus maintaining the correct ploidy from generation to generation. Many genes essential for meiosis are highly conserved in fungi, invertebrates, mammals, and plants. On the contrary, mechanisms governing the initiation of meiosis are diverse [1]. Unlike animals, plants lack a germ line pre-determined early in development. Therefore, understanding the molecular changes that specify the archesporial cells, the progenitors of meiotic cells, is crucial in defining the network of cellular processes leading to a successful switch from mitosis to meiosis.

**Figure 1:**
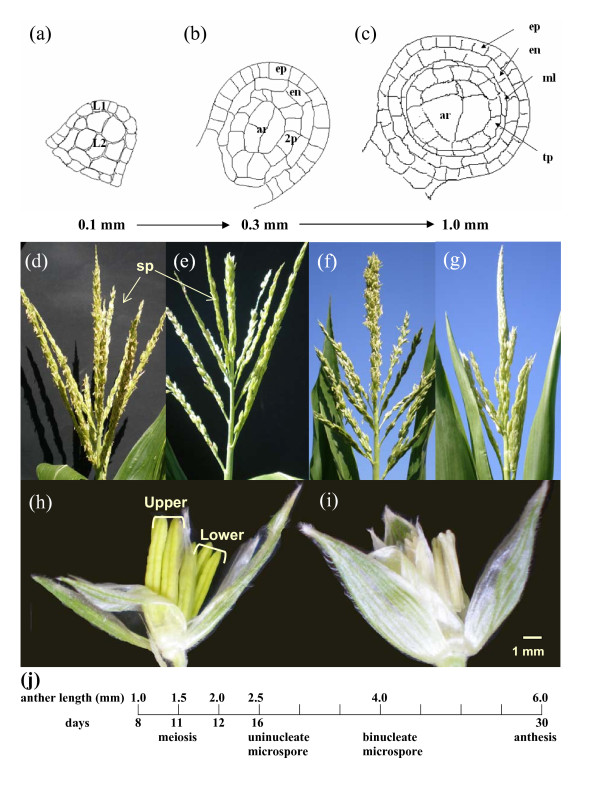
**Maize tassel and anther development**. Fertile maize anthers contain four locules. At ~0.1 mm there are about 50 cells in each locule. The line drawing of a transverse section **(a) **shows that the L2-derived (L2-d) cells are distinguishable from the L1-derived epidermis (ep) within a locule. At 0.3 mm **(b) **the L2-d has developed into endothecium (en), secondary parietal (2p) and archesporial cell types (ar). At 1.0 mm **(c) **middle layer (ml) and tapetum (tp) are apparent after a periclinal division of the 2p layer cells. Apart from the general morphology of no anther exertion, the mature tassel on the male-sterile *am1-praI *plant **(e) **is highly similar to a fertile sibling tassel **(d) **with the same number of tassel branches. In contrast, the *am1-489 *tassels **(g) **have fewer side branches (5-6) than fertile siblings **(f)**. The individual reproductive units on the tassel are spikelets (sp). There are two florets in each spikelet, and each floret contains 3 anthers **(h)**. The adaxial, upper florets used in the array experiment are developmentally 1 day ahead of the abaxial, lower florets. Anthers on a male sterile *am1-praI *plant **(i) **can reach 4-5 mm in length before they shrivel and degenerate; *am1-489 *anthers show growth arrest at 2-2.5 mm (data not shown). Based on cytological evidence and published results, anther sizes and the developmental timeline showing days after locular primordium initiation are diagrammed **(j)**.

Flowering is a late step in plant development, and nearly all floral cells are somatic. Mutants defective in the reproductive cells often have no or few defects in the soma and vice versa; once specified, the pre-meiotic cells can proliferate mitotically and then differentiate for meiosis successfully even when the surrounding somatic tissue is abnormal [2]. In Angiosperms meiotic cells originate from a handful of pluripotent somatic cells, derived from the L2 layer (L2-d) of a floral meristem. In maize (*Zea mays *L.), a few archesporial cells - the precursors of meiotically competent PMCs - are cytologically distinguishable by their central locular location and rapid enlargement when there are approximately 20 L2-d cells in locules of 150-170 μm anthers [3] (Figure 1a). Archesporial cells proliferate mitotically for several days (Figure 1b) then pause for 3 days as they mature into PMCs and functional meiocytes; concomitantly, the maize anthers grow from 1 mm to 1.5 mm (Figures 1c and 1j). L2-d cells that do not differentiate as archesporial cells form 3 somatic layers, each of a single cell type, that encircle the pre-meiotic population (Figure 1c). It is presently unclear how L2-d cells are programmed to be pre-meiotic within anthers or how this identity is retained during the mitotic divisions prior to PMC maturation. By 1.5 mm, prophase I has started and completion of meiosis depends on the correct expression of many genes [4,5]. Although archesporial cell divisions are asynchronous, maize meiosis is highly synchronized, likely facilitated by plasmodesmatal (cytoplasmic) connections among the PMCs.

Our focus is to understand the maturation of PMCs present in 1.0 mm anthers into meiocytes, that is, what processes are required for the cessation of mitosis, acquisition of meiotic competence, and initiation of meiosis? Many genes have been identified in flowering plants that are required for normal anther differentiation, but we are interested in defining the steps leading towards meiosis once the normal population of somatic and PMC cells are present. Several lines of evidence indicate that meiosis is set up as early as the pre-meiotic interphase [6-8], during the three day period of PMC maturation in maize anthers. Additionally, there may be differentiation steps preceding this stage that are not visualized cytologically.

Our entry point into analyzing meiotic competence is AM1; this is a maize nuclear protein required for normal PMC maturation [9], and it is necessary for both meiotic initiation and progression through early prophase I [10]. The actual roles of AM1 in the progression of meiosis at either the premeiotic/meiotic or the L/Z transition are still unclear at the molecular level. The closest homologs to AM1 are OsAM1 in rice [11] and SWI1/DYAD in *Arabidopsis thaliana *[12,13], but this gene is absent from both fungal and animal genomes. An analysis of double mutant combinations showed that *Am1 *is epistatic to many meiotic genes responsible for homologous chromosome pairing, including *afd1, dv1, ms43*, and *ms28 *[14]; additional meiotic processes impacted in *am1 *mutants include regulation of sister chromatid cohesion, telomere bouquet formation, recombination, synapsis, microtubule organization, and expression of other meiotic genes, particularly those acting during early prophase I [10].

Multiple *am1 *alleles have been distinguished cytologically [14,15], and all homozygotes are male-sterile. In all but one allelic state (*am1-praI*), central locular cells with PMC cellular anatomy undergo mitosis rather than meiosis. For example, the *am1-489 *allele contains a *Mu *insertion in an exon near the 3'-end; although the allele is transcribed, no protein is detected in mutant anthers [10]. The *am1-praI *allele encodes a semi-functional protein with a single amino acid substitution (R358W); the protein supports meiotic entry but PMCs fail to progress normally. In fertile maize, AM1 is expressed pre-meiotically and later loaded onto chromosomes during early prophase I; the defective AM1-PRAI protein remained mainly dispersed in nuclei and meiosis stalls at the L/Z transition [10]. The L/Z transition is a major step in meiotic progression during which pairing of homologous chromosomes is initiated as telomeres cluster on the nuclear envelope and double strand breaks are made as homologous recombination proceeds [16]. The *am1-praI *allele demonstrates that the L/Z transition is tightly regulated in plants, and it allows us to analyze this critical meiotic step in maize. The precise phenotypes of mutants are species-specific, but in each case AM1 and its homologs play a role in the regulation of early meiotic events. OsAM1 from rice [11] has a similar domain organization as maize, however, the SWI1/DYAD protein of Arabidopsis lacks N- and C-terminal domains found in AM1 and shares only 31% similarity in the central conserved PHD finger domain [12,13]. An RNAi inhibition of *OsAm1 *results in meiocytes that reach leptotene, similar to the maize *am1-praI *mutant [11]. Aberrant SWI1/DYAD1 proteins in Arabidopsis affect chromosome structure and cohesion during meiosis to various extents in both anthers and ovules [12,13,17] likely through meiotic chromosome remodeling [18]. The sister chromatids segregate unevenly during meiosis in *swi1-1/dyad *anthers, while in the ovule, meiosis is replaced by mitosis. Arabidopsis with specific *SWI1/DYAD1 *alleles show a small proportion of unreduced diploid gametes indicative of a meiotic or post-meiotic failure. This phenotype represents the first step (apomeiosis) to generate apomictic progeny, a novel prospect in plant breeding in which the progeny genotype is identical to the parent (meiotic failure class) or identical to a single meiotic product [19].

To better understand the required steps that normally occur during PMC maturation in preparation for meiosis and for progression through the critical L/Z transition, we compared the anther transcriptomes of mitotic *am1-489 *and L/Z meiotic arrest *am1-praI *lines using a custom Agilent 4 × 44 K microarray. Although *am1 *mutants affect both male and female meiosis, anthers were chosen because they are much easier to dissect than the female floral tissue and because comprehensive transcriptome studies of normal maize anther development are already available on this array platform [20-22].

## Results

### Development and morphology of *am1 *mutant tassels

To identify stages of anther development in fertile and male-sterile siblings relevant for this study, developmental progression was carefully followed for the two backgrounds harboring the *am1-praI *and *am1-489 *alleles. The male inflorescence (tassel) on a fertile maize plant bears hundreds of spikelets as shown in Figures 1d and 1f. When tassels reach maturity, the side branches held upright along the main spike relax to a lateral position, and pollen is shed from exerted anthers. The external morphology of *am1-praI *tassels (Figure 1e) is highly similar to a fertile sibling (Figure 1d) with the same number of tassel branches (8-10). In contrast, *am1-489 *tassels (Figure 1g) have fewer side branches (5-6) than their fertile siblings (8-10, Figure 1f). Delayed tassel development in *am1-489 *plants was also observed, because tassel branches remained upright about 5-7 days longer than in fertile siblings; tassels on *am1-praI *plants switched from upright to lateral simultaneously with fertile siblings.

Each spikelet contains two florets; each floret contains 3 anthers (Figure 1h) and each anther contains 4 locules. The upper floret is generally advanced 1 day relative to the lower floret. In our array experiment, only anthers from the upper florets were collected, and all measurements refer to this floret. Many male sterile mutants of maize with pre-meiotic defects exhibit delayed tassel maturation and anther growth arrest at about 2 mm (V. Walbot, unpublished data), that is, a failure to sustain anther growth during the three week period that follows completion of meiosis in fertile plants. Male sterile *am1-praI *anthers continue to grow and reach 4 to 5 mm in length (Figure 1i) at approximately the same time as fertile siblings. This indicates that the somatic cell layers of the *am1-praI *mutant anthers can sustain growth despite meiotic arrest. Sterile *am1-praI *anther locules are plump (not shown) until right before they degenerate (Figure 1g). In contrast, *am1-489 *anthers arrest at about 2 mm, the locules collapse, and anthers degenerate early as is typical of most *ameiotic1 *and other pre-meiotic male sterile mutants [9,22]. We conclude that somatic anther growth beyond 2 mm requires the presence of functional PMCs. We hypothesize that somatic anther tissues in *am1-praI *anthers perceive cues such as meiotic entry by the PMCs but progression through prophase I is not required to maintain anther growth.

### Cytological staging

**Figure 2:**
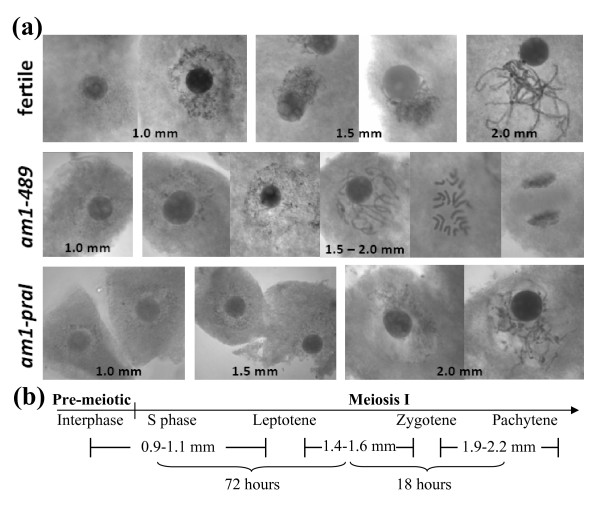
**Cytological staging of pollen mother cells (PMCs)**. **(a) **Acetocarmine-stained chromosomes in PMCs in anthers of various sizes. PMCs in both fertile and mutant anthers at 1.0 mm are mostly at interphase. PMCs in 1.5 mm fertile anthers are at the leptotene or zygotene stage of meiotic prophase I. PMCs in both 1.5 mm and 2.0 mm *am1-489 *anthers show unsynchronized, mitotic characteristics with 20 univalents; *am1-praI *central cells start meiosis successfully when anthers reach ~2.0 mm but arrest at the L/Z transition. **(b) **Developmental timing of meiotic stages at the corresponding anther lengths in fertile anthers.

Anthers were collected, fixed and later stained with acetocarmine to determine the cytological stage of PMCs in 1.0, 1.5 and 2.0 mm anthers (Figure 2). Both the 1.0 mm *am1-489 *and *am1-praI *PMCs were at pre-meiotic interphase at the 1.0 mm stage (Figure 2a) and cytologically normal. Archesporial cells in fertile plants finish most if not all the pre-meiotic mitotic divisions just before the 1.0 mm PMC stage, but the cells continue to expand in size from 1.0 to 1.5 mm as they mature [3]. Most PMCs of 1.0 mm fertile anthers were at interphase, but a few had advanced to early leptotene (Figure 2a). At 1.5 mm, the fertile PMCs are definitely in meiosis and achieve zygotene synchronously (Figure 2a), while the PMCs in *am1-489 *were found at various stages of mitosis (unsynchronized) with twenty unsynapsed chromosomes (Figure 2a). At 2.0 mm *am1-489 *PMCs continued one or more rounds of mitosis (Figure 2a) as previously reported [15].

*am1-praI *PMCs are delayed in maturation and remained at interphase (Figure 2a) at 1.5 mm. Eventually they start meiosis synchronously by the 2.0 mm stage (Figure 2a), about 18 h after the normal 1.5 mm stage (Figure 2b, [20]; the PMCs then arrest at the L/Z transition. Therefore, *am1-praI *PMCs are delayed in meiotic entry and progress more slowly through the initial meiotic stages.

### On/Off transcriptome differences in fertile and mutant anthers

Probes were scored as present (On) or absent (Off) as described in Methods. Maize anthers express an astonishing number of genes: the Agilent 4 × 44 K maize array has routinely detected expression of ~30,000 genes in both 1.0 mm and 1.5 mm anthers [21,22]. Because the two *am1 *alleles examined here are in different genetic backgrounds (see Methods) and maize inbred lines exhibit high levels of gene presence/absence and copy number variation [23] we first investigated the congruence of anther transcriptomes between the fertile siblings in these two lines. The majority of the transcriptomes (91% at 1.0 mm and 88% at 1.5 mm) are shared between the two fertile backgrounds (Table 1). Average variation of ~7% in gene content is reported for inbred lines [23], and we conclude that many of the differences observed in the two backgrounds are attributable to maize diversity. To minimize background effects in the On/Off analysis of gene expression, we have compared mutant anther transcriptomes directly to fertile siblings.

**Table 1:** Numbers of expressed genes with various patterns.

1.0 mm					1.5 mm				
***489*S**	***489*F**	***pra*S**	***pra*F**	**Genes**	***489*S**	***489*F**	***pra*S**	***pra*F**	**Genes**

Off	Off	Off	On	856	Off	Off	Off	On	389
Off	Off	On	Off	37	Off	Off	On	Off	1018
Off	Off	On	On	462	Off	Off	On	On	2519
Off	On	Off	Off	135	Off	On	Off	Off	150
Off	On	Off	On	**211**	Off	On	Off	On	**57**
Off	On	On	Off	3	Off	On	On	Off	81
Off	On	On	On	**135**	Off	On	On	On	**3487**
On	Off	Off	Off	491	On	Off	Off	Off	64
On	Off	Off	On	469	On	Off	Off	On	21
On	Off	On	Off	17	On	Off	On	Off	31
On	Off	On	On	231	On	Off	On	On	216
On	On	Off	Off	647	On	On	Off	Off	242
On	On	Off	On	**1720**	On	On	Off	On	**60**
On	On	On	Off	23	On	On	On	Off	67
On	On	On	On	**27196**	On	On	On	On	**24468**

*489*: *am1-*489; *pra*: *am1-praI*; F: fertile; S: sterile. Bold values are the common expressed sets in both *489 *and *pra *backgrounds.

At 1.0 mm *am1-489 *anthers are cytologically similar to fertile siblings; however, they are missing 2% (484/30,070) of the transcripts present in fertile siblings, which is above the estimated 0.13% false discovery rate (see Methods). Additionally, 4% (1,208) are ectopically expressed compared to fertile siblings (Table 1). These ectopically expressed genes include those that contribute to continued mitosis by the PMCs of *am1-489 *anthers. At 1.0 mm *am1-praI *anthers are also similar to fertile cytologically, but there are ~10% fewer [(31,280-28,104)/31,280] transcript types and ectopic expression of 80 genes (0.26%). Some of these transcriptome differences at 1.0 mm could reflect linkage disequilibrium (allelic variation between fertile and sibling sterile plants at loci linked to *am1 *alleles) or phenotypes too subtle to visualize by microscopy. As is explicated below, the major contributor to the transcriptome difference in *am1-praI *is the developmental delay in this mutant.

PMCs in 1.5 mm fertile anthers are at early prophase I between the leptotene and zygotene stages, somatic cells have differentiated, and somatic cell division has slowed. At 1.5 mm the more severe *am1-489 *anthers are missing more than 3,700 transcripts (~13%) expressed in fertile siblings (Table 1). Among these, 3,487 are also expressed in both *am1-praI *mutant and fertile siblings, hence among the thousands of loss-of-expression cases should be genes involved in meiotic entry. In contrast, just over 500 (< 2%) genes are off in *am1-praI *compared to fertile siblings (Table 1). The transcriptome data support the cytological observation of delayed meiotic entry in *am1-praI*, because 80% (2,634) of the transcripts missing at 1.0 mm are expressed by 1.5 mm. A linkage tree of the global transcriptome including all 8 sample types also demonstrated the transcriptomes are most divergent between the two backgrounds while the transcriptome differences between mutant and fertile samples at 1.0 mm are the least. In both backgrounds, the transcriptome of the mutant samples is more distinctive from fertile at 1.5 mm than at 1.0 mm (Additional File 1). Hence, it is important to exclude background difference before comparing the two alleles.

The lists of On/Off genes were evaluated for the presence of known meiosis-associated genes, but none were found. Thus although the *am1 *mutant alleles impact expression of ~10% of maize genes through ectopic expression and loss-of expression, these transcriptome differences must relate primarily to somatic cell processes. This demonstrates that meiotic genes are expressed in sterile *am1 *anthers but their expression levels could still be affected.

### Up- or down-regulated genes between stages or between alleles

**Figure 3:**
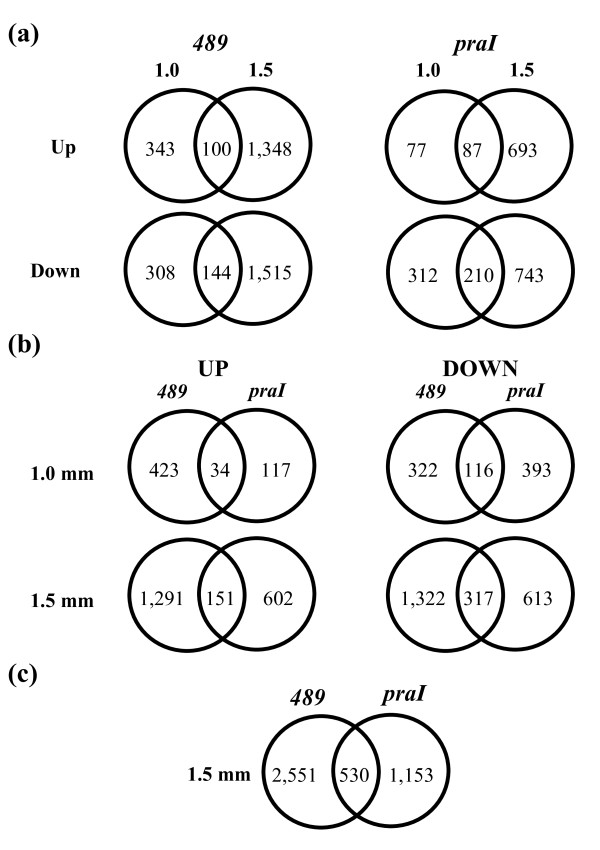
**Differential expression between mutant and fertile anthers**. Venn diagrams of up- or down-regulated genes in *am1 *alleles compared to the common fertile dataset. Number of transcripts unique to a stage or allele or expressed in common: **(a) **between two stages; **(b) **between alleles; and **(c) **comparison of the two mutant alleles at 1.5 mm, showing the number of differentially expressed transcripts unique to each allele and the common set (including the oppositely regulated with duplicates removed).

In addition to On/Off differences, we also evaluated transcript types that were expressed (On) and at least 1.5-fold distinct from the comparator with a p-value < 0.05 (see Methods). To minimize false discovery resulting from background differences while comparing the two mutant anther types, only transcript types detected in both fertile backgrounds (common fertile set: 29,262 at 1.0 mm and 28,072 at 1.5 mm; highlighted in Table 1) were used in the evaluation of up- or down-regulated genes. Both mutant anthers had about 4 times more genes differentially expressed at 1.5 mm than at 1.0 mm (Figure 3a); most transcripts are stage-specific confirming the distinctive properties of these two developmental stages [20]. The greater transcriptome abnormalities at 1.5 mm parallel the more aberrant mutant phenotypes at this stage. A subset of these transcripts is likely essential for entry and progression through early meiosis. The *am1-489 *mutants had about twice as many genes up- or down-regulated than *am1-praI *at 1.5 mm (Figure 3b), confirming molecular consequences of the more severe phenotype of *am1-489*. The 3,358 differentially expressed genes unique to *am1-489*, regardless of stage, are likely involved in meiotic entry or conversely highlight genes involved in the suppression of mitosis. The majority of these transcriptome differences (2,613, 78%) are found at 1.5 mm, including six meiotic genes, i.e., MEL1, SPO11-1, OSD1a, RPA70 (RPA1a), PS1, and MSH5.

In addition to the numerous allele-specific, differentially regulated genes, there are a total of 530 differentially expressed genes common to *am1-489 *and *am1-praI *at the 1.5 mm stage (Figure 3c). These include the non-redundant 468 genes that are differentially expressed in both (Figure 3c) and 62 oppositely regulated transcripts ("up versus not up" or "down versus not down"). Genes mis-regulated in both mutants at both stages (8 overlapping areas in Figure 3) are good candidates for common transcriptome responses of *am1 *mutant anthers relating to progression through the L/Z transition. The non-redundant set of 468 genes found at 1.5 mm is of particular interest, because this is a critical point of meiotic progression. Maize homologs of well known meiotic genes, e.g., *Skp1B, Zip4/Spo22-like*, *Rad54-like*, and meiosis-specific cyclins [24-32], show transcriptional mis-regulation in the *am1 *mutants.

Based on cytological evidence, development of PMCs in *am1-praI *is delayed then arrests while PMCs in *am1-489 *anthers undergo a mitotic division instead of a meiotic one. Therefore, the transcriptome changes in *am1-489 *anthers compared to fertile at 1.0 mm might also be important for meiotic entry. *Afd1 *(*Rec8/Rad21 *homolog), and *RPA70*, for example, are moderately down-regulated in *am1-489 *at 1.0 mm but not down in *am1-praI *at 1.5 mm. Homolog of *Afd1 *encodes protein associated with the cohesion complex, which establishes sister chromatid cohesion [33-35], while *RPA70 *is implicated in replication, repair, and transcription [36]. We also noted an RNA helicase SDE3 in this group, known for its role in post-transcriptional gene silencing via RNAi [37] and shown to be associated with meiosis in worms, flies, and mammals [38-40]. A heat map of all the meiotic genes included on this array (a total of 78 probes spanning 45 genes) is shown in Additional File 2.

Our results indicate that AM1 protein attenuates the expression level of many meiotic genes and other somatic genes that are crucial for anther maturation. Anther development is delayed (*am1-praI*) and ultimately stalled, causing male sterility.

### Data validation

A pilot array experiment was conducted on the same array platform prior to the study reported here. Anthers from families segregating for *am1-489 *and *am1-praI *were used in the pilot experiment and only male sterile anthers at 1.0 mm and 1.5 mm with 4 biological pools for each sample type were included. We compared data from the pilot experiment and found more than 90% of the pilot data corroborated results presented here (Additional File 3).

To further validate quantification and classification of key transcripts, a panel of 11 meiosis-associated genes including *Am1 *was analyzed by qRT (quantitative RT-PCR). Ten of them showed congruent results with the array data (Table 2 and Additional File 4). The one exceptional transcript (*Spo11-1*) was scored as borderline up-regulated (1.5-fold criterion, log2 = 0.6) in *am1-489 *mutant versus fertile at 1.5 mm on the array but was not significantly changed based on qRT results. Notably we checked the expression level of *Am1 *itself. The qRT analysis confirms the over-expression of the transcript in *am1-praI *in 1.5 mm anthers observed in the microarray data. It is likely that the temporal arrest at the L/Z stage and the inefficient loading of AM1-PRAI onto the chromosomes, when *Am1-praI *is highly expressed, lead to message accumulation in meiocytes. The increased transcript abundance is also reflected in at least 2 times higher levels of AM1-PRAI protein in tassels containing 1.0-3.0 mm anthers than in wild type siblings at these stages (Additional File 5).

**Table 2:** A panel of meiotic genes and their expression patterns in *ameiotic1 *(*am1*) anthers.

Maize Gene ID	Names	Homolog	Stage	qRT Verified	Differentially Expressed
GRMZM2G075563	*Ms5*	plant only	various		down in *am1-489 *at 1.5 mm
GRMZM2G059037	*Afd1*	*Rec8/Rad21*	prophase I	√	down in *am1-489 *no change in *am1-praI *at both stages
GRMZM5G883855	*Am1*	plant only	pre-meiotic	√	down in *am1-489*; up in *am1-praI *at both stages
GRMZM2G109618	*Dmc1*	*Dmc1*	prophase I	√	down in both mutant alleles at 1.5 mm
GRMZM2G408897	*Mmd1*	plant only	early-late		down in both mutant alleles at 1.5 mm
GRMZM2G060394	*Skp1A*	*Skp1A*	prophase I	√	significantly down in both mutant alleles at 1.5 mm
GRMZM2G032562	*Skp1B*	*Skp1B*		√	significantly down in both mutant alleles at 1.5 mm
GRMZM2G143590	*Zyp1*	*Zip1*	prophase I	√	down in both mutant alleles at both stages
GRMZM2G083138	*Rad54-like*	*Rad54-like*	prophase I	√	down in both mutant alleles, particularly at 1.5 mm
GRMZM2G100103	*Phs1*	*Rec114*	prophase I		low or no expression in all
GRMZM2G129913	*Spo11-1*	*Spo11*	prophase I	nc	marginal changes
GRMZM2G454838/GRMZM2G328795	*Zip4/Spo22-like*	*Zip4/Spo22-like*	prophase I		down in both mutant
GRMZM5G883855	*Aml1*	*Mei2*	prophase I		no changes
GRMZM2G102242	*Mnd1*	*Mnd1*	prophase I		no changes
GRMZM2G308884	*Prd1*	*Mei1*	prophase I		no changes
GRMZM2G121543	*Rad51A*	*Rad51*	prophase I	√	no changes
GRMZM2G084762	*Rad51A2*	*Rad51A2*	prophase I	√	no changes
GRMZM2G115013	*RPA70*	*RPA1a*	various	√	down in *am1-489 *at 1.0 and 1.5 mm
GRMZM2G315902	*Mlh3*	*Mlh3*	prophase I		no changes or not significant
GRMZM2G055899	*Pair1a*	plant only	pre-meiotic		not significant
GRMZM2G056075	*Msh5*	*Msh5*	various		not significant except up in *am1-489 *at 1.5 mm
GRMZM2G457370	*Ago1*	*Ago1*	pre-meiotic		up in both mutant alleles at both stages

√: qRT analyses found the same direction of regulation and approximately the same magnitude of change as calculated from the microarray analysis; nc: qRT and array results were non-congruent.

### Gene ontology (GO) annotation of differentially regulated transcripts

**Figure 4:**
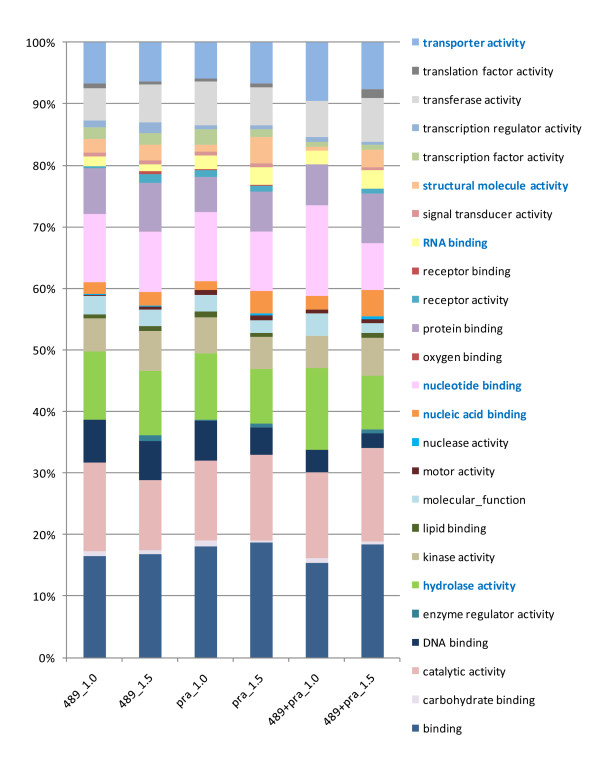
**Gene ontology annotation**. Percentage of each gene ontology category of differentially expressed transcripts in mutant anthers compared to fertile (excluding unknowns) found in: **489_1.0**: 1.0 mm *am1-489*; **489_1.5**: 1.5 mm *am1-489*; **pra_1.0**: 1.0 mm *am1-praI*; **pra_1.5**: 1.5 mm *am1-praI*; **489+pra_1.0**: both *am1-489 *and *am1-praI *at 1.0 mm; **489+pra_1.5**: both *am1-489 *and *am1-praI *at 1.5 mm. Categories are in alphabetical order, starting from the bottom of the list.

GO terms were assigned using Agbase http://www.agbase.msstate.edu, where more than 50% of the probes have one or more terms assigned. Figure 4 illustrates the classification of differential transcripts excluding the unknown category, which are included in the list of gene counts in Additional File 6. Several functional groups are prominent. Genes with catalytic activity and transporter activity are affected at 1.0 and 1.5 mm in both *am1-489 *and *am1-praI *anthers. At the pre-meiotic 1.0 mm stage, genes with hydrolase activity or nucleotide binding accounted for ~30% of the genes with known functions. These groups are likely critical in PMCs at interphase when cells are preparing for the cell cycle, including cell growth, DNA replication, and organelle multiplication and for the proper differentiation of somatic anther cells. Although no major cytological differences were observed between mutant and fertile anthers at this stage, many cell processes already appear to be compromised at the start of PMC maturation. Genes with nucleic acid binding activity are likely essential for L/Z transition. We conclude that a switch of cell processes programmed by new gene expression is necessary for subsequent meiotic entry and progression by the PMC and for normal development of somatic cell types.

### AM1 affects the transcriptomes of meiocytes and tapetum

Based on cell counts, the PMCs constitute less than 1% of anther cells at the 1.0 and 1.5 mm stages in maize [3]. In transverse sectional area, however, these giant cells comprise 33% of the locule (Figure 1c) and about 10% of the anther (data not shown). Using data in Tang et al. 2010 [41] reporting the yield of total RNA from isolated rice PMCs we calculate that rice PMCs contain at least 25 times more RNA than a typical cell; similarly using data in Chen et al. 2010 [42], Arabidopsis PMCs must contain at least 100 times more RNA than typical diploid cells. Therefore in analyzing whole anthers, the expectation would be that 22 - 53% of extracted RNA would be derived from the PMC population. As maize is more closely related to rice and anther size and morphology are also more similar, we take a figure of about 20% as the expected PMC contribution to total RNA. Based on these assumptions, transcripts enriched 4-5 fold in fertile anthers compared to either *am1 *mutant are likely to be PMC-enriched or PMC-specific transcripts.

Nonetheless, a substantial fraction of the 4,587 transcriptome changes documented in Figure 3b (with 496 redundant ones removed from the 5,083 total) as specific to either *am1-489 *or *am1-praI *compared to their fertile counterparts could occur in somatic tissue surrounding the pre-meiotic cells. Male-sterile mutants with tapetal defects are very common (c.f. [22] references therein) because these nurse cells play critical roles in relaying nutrients and structural components to the PMCs. Although not investigated to date, the absence of proper PMC maturation could readily impact neighboring tapetal cell differentiation over the 1.0 to 1.5 mm growth period as was hypothesized for the On/Off class of transcripts. As a first step in assigning transcripts to the pre-germinal or somatic tissues, we cross checked against an independent array data set for laser-dissected pools of PMCs or tapetal cells from fertile anthers at 1.5 mm (T. Kelliher and V. Walbot, unpublished data). PMC-enriched genes were defined as those expressed at least 2 times higher in the PMCs than the surrounding tapetum; conversely, those expressed at least 2-fold higher in the tapetum compared to PMCs constitute the tapetum-enriched class. Of the mis-regulated genes in whole anthers unique to either allele (N = 4,587), 13% (N = 613) were found in the PMC-enriched class; 10% (N = 486) were in the tapetum-enriched class. Considering each allele separately, of the 3,174 non-redundant *am1-489 *specific changes, 10% (N = 325) are in the PMC-enriched class and 12% (N = 393) are tapetum-enriched. A different proportion is found in *am1-praI*: of the non-redundant *am1-praI *specific changes (N = 1,577), 20% (N = 317) are PMC-enriched and 7% (N = 107) are tapetum-enriched. We conclude that AM1 has a major impact on tapetal differentiation during PMC maturation, with a higher ratio of tapetal defects in *am1-489 *than in *am1-praI*; we hypothesize that the more substantial alterations in tapetal cells in *am1-489 *ultimately lead to under-developed, shorter *am1-489 *mutant anthers. We hypothesize that genes found in the combined list of PMC- and tapetal-enriched genes are likely to be important for normal growth of other anther cell types. For both *am1 *mutant alleles, this class comprises 75% of the mis-regulated genes indicating that mis-differentiation of the PMCs has a major impact on somatic anther cells.

Among the 530 genes mis-regulated in both mutants, 297 (56%) detected in whole anthers are in the PMC-enriched class while only 1% are expressed ~2 times higher in tapetum. AM1 protein was previously detected by immunolocalization only in meiocytes and not in the adjacent tapetal layer in fertile meiotic anthers [10], and the *Ameiotic1 *transcript intensities detected in the PMCs (early meiosis) are about 4-5 times higher than in the tapetum at 1.5 mm. Therefore, the absence of AM1 or the presence of an aberrant protein has a major impact on PMC gene expression. Sixty-four of the highly PMC-enriched genes are listed in Table 3; at the criterion of 4 fold or higher in meiocytes than tapetum at 1.5 mm many of these genes could be PMC-specific based on our estimate of the expected contribution of PMC RNA to total anther RNA. Members include *Am1*, *Skp1B*, *AGO2 *[43], and *UBA *(ubiquitin-associated) genes.

**Table 3:** List of L/Z transition genes.

Maize Est	Maize Gene ID	Description
AI692111	AC198518.3_FG002	Sterile alpha motif domain family protein
AW231811	GRMZM2G033236	ND
BM378145	GRMZM2G041418	Putative NADH dehydrogenase
BM500607	GRMZM2G007736	Alpha-trehalose-phosphate synthase (UDP-forming)
CB280793	GRMZM2G029385	Mitochondrial import inner membrane translocase subunit
CD436448	GRMZM2G342807	Unknown protein
CF059625	GRMZM2G149347	Nucleic acid binding zinc finger CCCH type
CF626131	GRMZM2G454838/GRMZM2G328795	ZIP4/SPO22-LIKE
CN844996	GRMZM2G149392	Unknown protein
DQ663482	GRMZM5G883855	**Ameiotic1**
DR830496	GRMZM2G113631	ND
DT946613	GRMZM2G310739	Glucan endo-1,3-beta-glucosidase 5
TC279480	GRMZM2G041328	Histone-arginine methyltransferase CARM1
TC279550	GRMZM2G146206	Triosephosphate isomerase, cytosolic
TC280195	GRMZM2G134708	Pyridine nucleotide-disulphide oxidoreductase
TC281079	GRMZM2G027173	Phagocytosis and cell motility ELMO domain-containing protein 2
TC282924	GRMZM2G107495	Unknown protein
TC283097	GRMZM2G050684	CBS (cystathionine beta-synthase) domain containing protein
TC284163	GRMZM2G347717	UDP-glucuronic acid decarboxylase
TC284639	GRMZM2G122965	Unknown protein
TC286486	GRMZM2G071630	Cytosolic glyceroldehyde-3-phosphate dehydrogenase GAPC3
TC287318	GRMZM2G028369	Chorismate mutase type II
TC287640	GRMZM2G139941	ND
TC287858	GRMZM2G360681	Heat-shock protein 101
TC288590	GRMZM2G097135	BAG domain containing protein (BCL-2-ASSOCIATED ATHANOGENE 5)
TC289172	GRMZM2G047372	Unknown protein
TC289341	GRMZM2G116243	Calcineurin B subunit-related
TC289354	GRMZM2G052474	Similar to NC domain-containing protein-related
TC289458	Unannotated	ND
TC289461	GRMZM2G140994	Rhomboid domain containing 1
TC289712	GRMZM2G071739	Ubiquitin-associated (UBA)/TS-N domain-containing protein
TC289753	GRMZM5G878823	Putative RNA-binding protein RNP-D precursor
TC290471	GRMZM2G109496	Probable protein phosphatase 2C
TC291009	GRMZM2G032562	**SKP1-like protein 1B**
TC295884	GRMZM2G148872	SOUL heme-binding protein
TC296255	GRMZM2G080930	Putative RSZp22 splicing factor
TC296845	GRMZM2G067350	4/1 protein
TC297030	GRMZM2G020766	Pseudogene of a potassium transporter
TC298798	GRMZM5G889299	Unknown protein
TC301446	GRMZM2G045970	Cytidylyltransferase family protein
TC301790	GRMZM2G354827	Benzothiadiazole-induced somatic embryogenesis receptor kinase 1 (SERK1)
TC302695	GRMZM2G017388	Cation/calcium exchanger 4
TC303479	GRMZM2G007791	Argonaute protein, similar to AGO2
TC303749	GRMZM2G020766	Pseudogene of a potassium transporter
TC304331	GRMZM2G457370	AGO1
TC305157	GRMZM2G159724	NADP-dependent malic enzyme
TC305158	GRMZM2G159724	NADP-dependent malic enzyme
TC305266	GRMZM2G007300	Ubiquitin-conjugating enzyme E2 UBC7
TC305399	GRMZM2G050329	SPX (SYG1/Pho81/XPR1) domain-containing protein
TC305717	GRMZM5G801875	MATE efflux family protein
TC306072	GRMZM2G179215	ND
TC306331	GRMZM2G349996	ND
TC307437	GRMZM5G832378	ND
TC308672	Unannotated	ND
TC309747	Unannotated	ND
TC309993	GRMZM2G001803	ND
TC310318	GRMZM2G306679	17.4 kDa class I heat shock protein 3
TC310688	GRMZM2G178960	Ribulose-phosphate 3-epimerase
TC310843	GRMZM2G170927	Vacuolar proton pyrophosphatase
TC312974	GRMZM2G351810	Unknown protein
TC314126	GRMZM2G043992	Unknown protein
TC314427	GRMZM2G125775	AN17; zinc finger (AN1-like)-like protein
TC314676	GRMZM2G119523	Unknown protein

These are genes affected in anthers with either mutant allele compared to fertile anthers at 1.5 mm and are predominantly expressed (four times or higher) in PMCs than in tapetum at 1.5 mm.

Items in bold are known genes previously reported to be associated with meiosis or meiosis-related processes; ND: not determined.

Maize homologs of well known meiotic genes show transcriptional mis-regulation in the *am1*-mutants (Additional File 7): *Skp1*, an essential component of the ubiquitin-ligase complex involved in cohesion distribution and nuclear re-organization during leptotene to pachytene stage [24,26,44]; *Rad54*, a *Rad51 *partner involved in chromatin re-modeling and repair of DNA double-strand breaks [28,29,45]; *Msh4 *and *Zip4/Spo22-like*, required genes for interferent crossing-over formation [27,46]; and *Zyp1*, the central element of the synaptonemal complex that starts to assemble on chromosome at the L/Z transition [47]. We also note the mis-expression of the meiotic cyclin SDS essential for the control of meiotic prophase I progression in Arabidopsis and rice [48].

### K-median clustering of PMC-enriched genes

**Figure 5:**
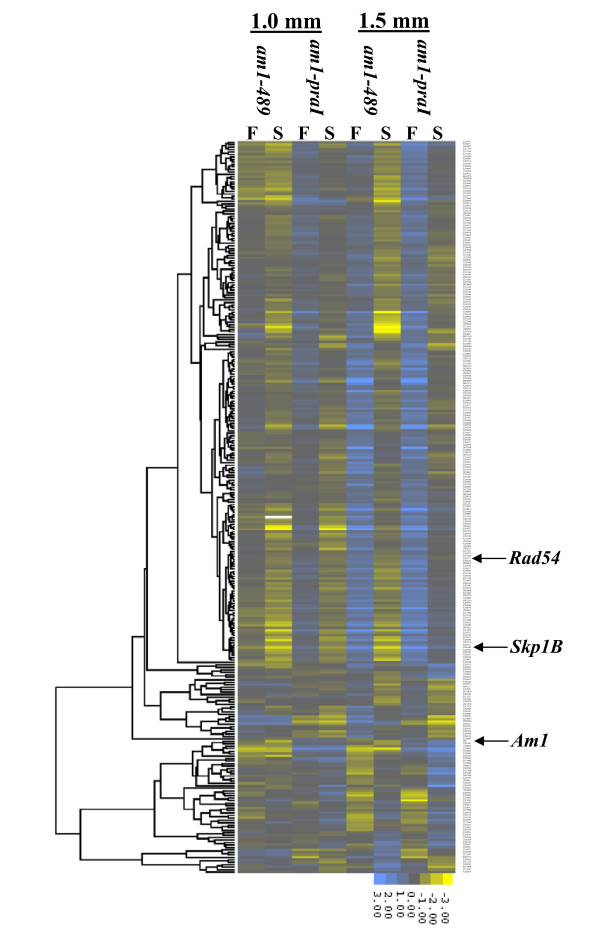
**Heat map of 297 PMC-enriched, differentially expressed genes in mutant (S) versus fertile (F) anthers**.

**Figure 6:**
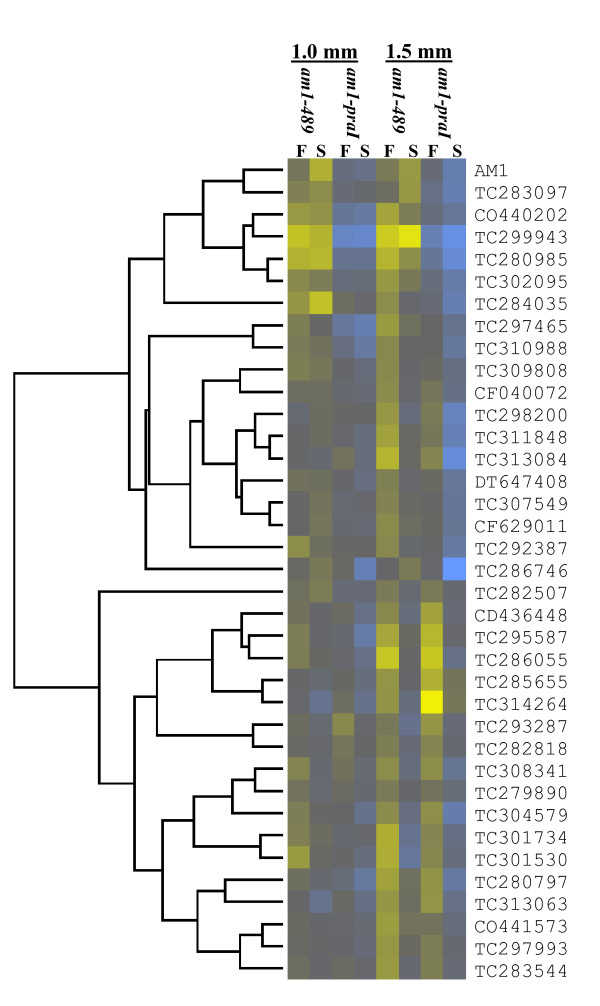
**Heat map of 37 PMC-enriched genes that clustered with the *Am1* gene expression profile as in Figure 5**.

**Figure 7:**
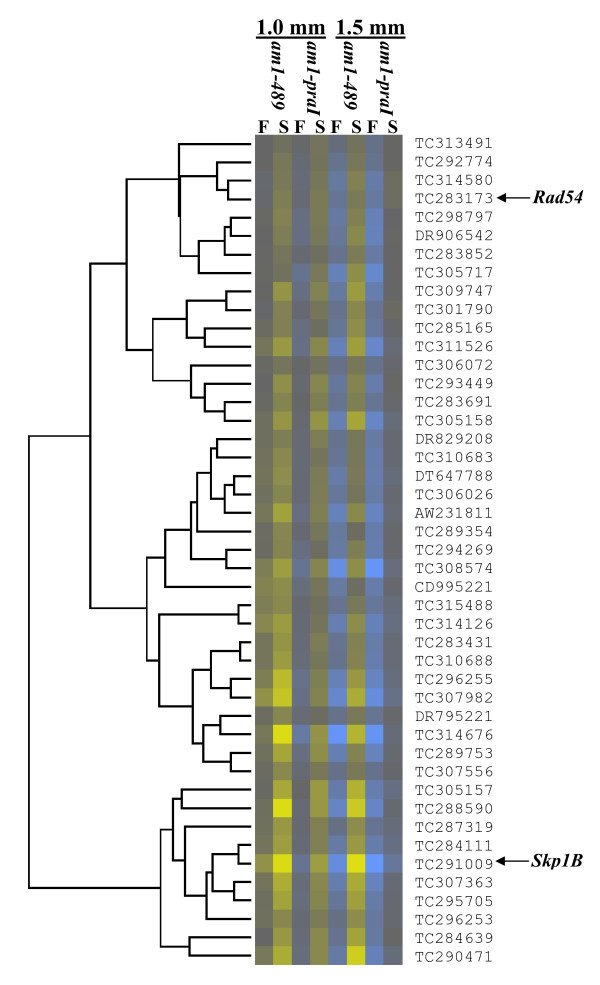
**Heat map of 45 PMC-enriched genes that clustered with the *Skp1B *and *Rad54 *expression profiles as in Figure 5**.

The 297 PMC-enriched genes differentially expressed in both mutants (Additional File 7) were clustered with algorithms from the Cluster 3.0 program http://bonsai.hgc.jp/~mdehoon/software/cluster/. K-median clustering based on Euclidean distance, uncovered several interesting clusters, and these are displayed in a heat map (Figure 5). A group of 37 genes were up-regulated in *am1-praI *but down-regulated in *am1-489 *anthers compared to fertile at 1.5 mm (Figure 6 and Additional File 8). A subset of these parallel *Am1 *expression, including genes homologous to Agamous-like MADS-box transcription factor [49], cell cycle associated Mob1-like [50], SPX domain-containing, zinc finger (C3HC4-type RING finger)-related, and Suppressor of Gene Silencing 3 (SGS3) [51,52] homolog (Additional File 9). These genes could have high relevance for meiotic entry or suppression of mitosis. Genes that have similar patterns in both *am1-489 *and *am1-praI *could be essential for meiotic prophase I progression. For instance, 45 genes are down-regulated in both mutants and cluster with *Skp1B *and *Rad54-like *(Figure 7 and Additional File 10). Other clusters contain genes with defined meiosis-related functions include *Mmd1*, *Zyp1, Apospory*, and *Werner syndrome ATP-dependent helicase *gene families [53-56].

## Discussion

Meiotically competent cells in plants differentiate late in floral ontogeny. Presently, the steps required for specification of meiotic competence in a tiny number of floral somatic cells are unknown. However, nuclear events in meiotic cells are readily distinguishable from those in mitotic cells, and the developmental program of archesporial cells and PMCs is independent of successful somatic cell development at least through progression into prophase I [2]. The *ameiotic1 *mutants of maize demonstrate that conducting meiosis is distinguishable from the preceding PMC differentiation events such as cell and nuclear enlargement and a rounded cell shape. In all *am1 *mutant anthers, archesporial cells are normal and proliferate to make the normal numbers and morphology of highly enlarged PMCs present in 1.0 mm maize anthers; somatic cell differentiation is also cytologically normal. For all *am1 *alleles except *am1-praI*, the PMCs remain mitotic rather than switching to meiosis, suggesting that both repression of mitosis and activation or maintenance of meiotic programs could be required to enter meiosis and that AM1 contributes to these processes. Cells in the somatic layers are still dividing at 1.0 mm but most layers have stopped dividing by 1.5 mm [3]; the somatic cessation of mitosis does not require AM1.

Global transcriptome analysis reinforces distinctions in the anatomical phenotypes of *am1-489 *and *am1-praI*. Both mutants have few cytological and moderate transcriptome differences compared to fertile at 1.0 mm. At 1.5 mm fertile PMCs have entered meiosis while mutant PMCs have not; unsynchronized mitosis is observed in *am1-489 *PMCs, and these cells in *am1-praI *are still at the pre-meiotic interphase. The mutant transcriptomes are more distinctive compared to fertile anthers at 1.5 mm. A comparison of the *am1-praI *profiles at 1.0 and 1.5 mm confirms that these anthers are developmentally delayed at 1.0 mm and that a substantial fraction of the transcriptome expected at 1.0 mm appears later at 1.5 mm. These results indicate that AM1 has an early impact on the pace of PMC maturation that is critical for timely meiotic entry.

From profiling we discovered maize genes associated with meiotic entry and the L/Z transition in PMCs using three methods: analysis of meiosis-associated genes, K-median clustering, and comparison to transcripts detected in isolated normal PMCs. Many meiosis-associated transcript types identified in our transcriptome profiling study have been reported to play major roles in sister chromatid cohesion, synapsis, homologous recombination, and double-strand repair [57]. Based on homology to defined proteins, a region shared between SWI1/DYAD1 and AM1 is predicted to have DNA binding ability like other PHD finger homeodomain proteins [10] and likely to be involved in regulating transcription. Our array data clearly demonstrate that AM1 attenuates the expression of other meiotic genes. Interestingly, none of the meiosis-associated genes are regulated in an absolute On/Off pattern, somewhat surprising given that *am1-489 *PMCs perform mitosis instead of meiosis. These results redefine a role of AM1 in the modulation of transcript accumulation for many critical meiotic genes rather than simply switching them on or off. This could also imply that many meiotic genes including those associated with DNA repair are expressed whether or not meiosis will proceed; this distinguishes plants from other well-studied eukaryotes.

One critical step to distinguish meiosis from mitosis is homologous chromosome pairing. Only during meiosis are homologous chromosomes held together by the formation of the synaptonemal complex and remain attached until meiosis prophase I has completed by the formation of chiasmata, the cytological manifestation of crossovers. Extensive studies have identified numerous genes involved in these processes after meiotic entry [58], and several genes highlighted in comparing *am1 *to fertile are in this category (Additional File 2). Transcript types affected only in *am1-489 *include those with homology to *Afd1*, *RPA70*, *Ms5*, and *Msh5 *gene families. Transcript differences found in both mutant anthers include those with homology to *Skp1B*, *Rad54-like*, *Msh4*, *Sds*, and *Zyp1*. AFD1, a maize REC8 homolog acting downstream of ASY1/HOP1, is associated with the maintenance of the axial/lateral elements of the synaptonemal complex and regulation of sister chromatid cohesion [33]. Pawlowski et al. [10] found that AFD1 is loaded onto the chromatin in *am1-praI *but not in *am1-1 *PMCs. Both the array and qRT data showed a moderate yet significant reduction of *Afd1 *transcripts at 1.0 mm in *am1-489*, which is phenotypically similar to *am1-1*, but not in *am1-praI*. *Dmc1*, a *RecA *homolog, is involved in meiotic double strand DNA break repair [59]. In plants *Dmc1 *transcripts are found in PMCs and ovule meiotic cells [60] and sporadically in mitotic cells [61]. The maize *Dmc1 *homolog analyzed is down-regulated in *am1-489 *but not in *am1-praI *compared to fertile. Thus the single amino acid mutation in the *am1-praI *protein does not impact the transcript levels of *Dmc1 *or *Afd1 *genes. We can conclude that an important transcriptional regulation of *Afd1 *and *Dmc1 *occurs during meiotic entry but is not essential later during the L/Z transition. The status of the *praI *"meiocytes" is probably affected by another level of control for achieving a zygotene-like stage. Other mechanisms such as post-transcriptional regulation and/or nuclear entry of specific meiotic proteins might then be more important for meiotic prophase progression [62].

RAD51, another *RecA *homolog, is required for homology searching, chromosome pairing, DNA strand transfer (D-loop formation), and double-strand break repair in meiosis and in DNA repair in somatic cells [63]. RAD51 protein is absent in *am1 *mutants [10]. Neither *Rad51A *nor *Rad51A2 *transcript levels are affected, therefore, post-transcriptional regulation of RAD51 protein levels is likely. RAD54 is a branch migration protein that is involved in dissociating D-loops formed by DMC1 during meiosis as well as D-loops formed by RAD51 though less efficiently [45]. Interestingly, *Rad54-like *transcript is significantly reduced in both *am1 *mutants.

*Skp1B *of another known meiotic gene family is also down-regulated in both *am1 *mutants. The Arabidopsis *Skp1-like1 *(*ASK1*) gene was previously reported to perform a critical role in recombination during the leptotene to pachytene stages [24,25]. ASK1 protein is important for many cell processes including modifying and reconstructing meiotic chromatin [24,25] and telomere formation [26]. Telomere bouquets are missing in *am1-489 *and are retarded or incomplete in *am1-praI *[10]. Telomere clustering on the nuclear envelope is another crucial event of meiotic chromosome organization during prophase I that occurs at the L/Z transition in many organisms [64,65]. Based on clustering analysis, *Rad54-like *and *Skp1B *expression patterns are very similar and dependent on *Am1*. Therefore, all three proteins might work in concert to advance meiocytes through the L/Z transition, the stage when *am1-praI *is arrested.

Other interesting genes differentially expressed in both mutants compared to fertile that have been implicated in the meiotic process include *Always Early1 *[66], *Werner syndrome helicase *[56], *Zap-3 telomerase *[67], and *kinesin *[68]. Our analysis of these genes along with *Skp1B*, *Rad54-like *and *Am1 *shows a predominant expression in meiocytes (at least 5 times higher than whole anthers and at least 3 times higher than tapetum). This finding suggests that AM1 interacts directly or indirectly with these proteins in establishing or stabilizing meiotic chromosomes. Two good candidate genes for apomixis, *Afd1 *and *Brassinosteroid insensitive1-associated receptor kinase *genes [69,70], were down-regulated in *am1-489 *anthers at 1.0 mm and in both mutant anthers at 1.5 mm, respectively.

According to Palmer [9], *am1-1 *anthers degenerate after one or 2 rounds of ectopic PMC mitosis and fail by the ~ 2.2 mm anther stage. *am1-489 *anthers also cease growth, and this is similar to most other pre-meiotic male-sterile mutants [22]. Therefore, the continued growth of *am1-praI *anthers to 4-5 mm and their normal morphology indicate that the transcriptome differences are primarily causing defects in the PMCs and not significantly impairing somatic cell layers, despite inferred changes in the somatic and detected changes in the tapetal transcriptomes. We further conclude that meiotic entry by PMCs is sufficient to trigger somatic anther growth; the nature of the developmental signal is yet to be determined.

To more critically address the cell type specificity of transcriptome alterations, the 530 non-redundant genes differentially expressed in both *am1 *alleles were used to query gene lists derived from array profiling of laser microdissection-enriched PMCs or tapetal cells in normal anthers. Fifty-six percent were expressed at least 2-fold higher in PMCs compared to those found in the immediately adjacent tapetum. Our results indicate that either the complete absence of AM1 or the presence of excess aberrant AM1-PRAI protein has a profound impact on gene expression in the PMCs during the maturation phase from 1.0 to 1.5 mm anther length, including the period of meiotic entry up to the L/Z transition stage. Based on estimation of the contribution of PMC RNA to total anther RNA [41], we hypothesize that transcripts enriched 4-fold or higher could be PMC-specific. In fact, the highest enrichment observed was 9-fold, initially puzzling because PMCs are less than 1% of the cell population but should contain many unique transcripts associated with meiosis. After accounting for RNA contribution, however, enrichment of 4- to 5-fold in isolated PMC is the expectation.

Recently, multiple microarray or RNA-Seq analyses of purified meiocytes have been reported [5,41,42,71]. Using manual methods or laser capture microdissection, meiocytes relatively free of contaminating somatic cells were obtained in sufficient numbers for analysis. The limitation of these rice and Arabidopsis analyses was that all stages of meiosis were represented; even if there is little or no *de novo *transcription during meiosis, transcripts present initially in PMCs may have decayed during meiosis, and the mixed meiocyte population is hence not ideal for analysis. In these studies from 800 to 1,586 meiocyte-specific transcript types were identified. For maize, we used whole anther analysis and comparison to an independent dataset of laser microdissected PMCs and tapetal cells in carefully staged 1.5 mm fertile anthers at prophase I, and we also used the comparison of *am1-489 *and *am1-praI *to discover meiosis-associated genes. Prophase I spans only one day of the 30 days in maize anther development. From our analysis, 297 PMC-enriched genes were identified as mis-regulated by both mutants within the narrow window of prophase I. Additionally, we identified genes mis-regulated at the start of PMC maturation, particularly in the *am1-489 *mutant, that are likely associated with the suppression of mitosis and early preparation for meiosis.

Our *am1 *transcriptome data can serve as a resource to decipher the complex networks of meiosis initiation in plants. Comparing results from the two *am1 *alleles provides insights into multiple control points.

## Conclusion

Transcriptome changes in *am1 *anthers prior to meiosis lead to loss of meiotic competence in the PMCs. Transcript types affected only in *am1-489 *anthers and resembling the *Am1 *expression pattern likely define requirements for suppression of mitosis and entry into meiosis and ultimately influence the development and maturation of anther somatic layers; meiosis-associated processes affected in *am1-489 *mutants include genes important for sister chromatid cohesion, and RNA interference. Transcripts differentially regulated in both mutants are more likely related to progression through the L/Z transition. This microarray analysis indicates that maize meiotic prophase is controlled at multiple levels and that AM1 controls the RNA level of only a subset of meiotic genes at the critical steps of meiotic entry and the L/Z transition.

## Methods

### Plant materials

Two maize (*Zea mays *L.) stocks with different hybrid backgrounds containing the *am1-489 *(50% B73 + 25% A619 + 25% mixed other or unknown) or *am1-praI *allele (75% A619 + 25% mixed other or unknown) were propagated by male-sterile (*am1//am1*) × fertile sibling (*am1//Am1*) crosses or self-pollination of fertile heterozygotes (*am1//Am1*). Immature tassel branches were carefully removed from 4-6 week old plants and kept moist in wet paper towels until dissection. Anthers were recovered from upper florets with two pairs of sharp forceps, measured with a micrometer, and collected directly into screw-cap microcentrifuge tubes chilled in liquid nitrogen. Sixty 1.0 mm and thirty 1.5 mm anthers were collected for each biological replicate. For cytological staging, the anther locule contents were expelled onto a microscope slide using dissecting needles. Standard acetocarmine staining [72] was performed, and samples were observed at 400× with an oil lens to classify meiotic stages or confirm mitotic chromosomes.

### Microarray experiment

Total RNA was extracted using the TRIzol^® ^Plus RNA Purification Kit (Invitrogen, Carlsbad, CA) from each anther pool. RNA quality and quantity were examined on a Bioanalyzer^® ^(Agilent, Santa Clara, CA). Amplification and balanced dye-labeling of four biological replicates were performed according to manufacturer's recommendation. Three technical replicates for each sterile biological pool (12 hybridizations) and one technical replicate for each fertile biological (4 hybridizations) were performed. After hybridization on a customized maize 4 × 44 K array (Agilent ID 16047, original probe design as described in [20], the slides were washed, dried, and scanned on Agilent's High Resolution Microarray Scanner; data were processed with Feature Extraction Software. The resulting median foreground values for the red and green channels were normalized in two steps using the limma package [73] in R: "within arrays" using the lowess method and "Between arrays" using the quartile method. Probes with expression values greater than 3.0 standard deviations above the average foreground of the array's negative controls were considered "on", resulting in an estimated false discovery rate of 0.13%. However, probes with fewer than 75% of the replicate measurements scored as "On" were then excluded from further analysis. Significance for differential expression was set at ~1.5-fold (log2 ~ 0.58) with a p-value ≤ 0.05. Microarray data for these experiments are available online at GEO http://www.ncbi.nlm.nih.gov/geo/ under accession number GSE30149.

### Quantitative RT-PCR (qRT)

Transcript abundances were quantified from three biological replicates for each sample type and four technical replicates for each sample (12 RT reactions for each gene in each type of sample). First-strand cDNA was synthesized from 1 μg total RNA using Superscript III^® ^reverse transcriptase (Invitrogen). Diluted cDNA was amplified in a 20 μl qRT reaction containing 10 μl of iQ SYBR Green Supermix (Bio-Rad, Hercules, CA) and 250 nM of each primer on an Opticon2^® ^system (Bio-Rad). The cyanase gene was used as the constitutive internal standard [74]. Primer sequences and product sizes for the genes surveyed in qRT are listed in Additional File 11. The amplification protocol was 95°C for 5 min followed by 40 cycles of 95°C for 10 sec, 60°C for 30 sec, and plate read. Melting curve analysis was performed every 0.5°C from 55 to 95°C with a 10 sec hold at each step to check for production of a single amplicon. The cycle threshold (Ct) value and amplification efficiency were determined using PCR Miner version 2 [75].

## List of abbreviations

***Am1***: *Ameiotic1*; **L2-d: **derived from the L2 layer; **L/Z: **leptotene/zygotene; **PMCs**: pollen mother cells; **qRT: **quantitative RT-PCR.

## Competing interests

The authors declare that they have no competing interests.

## Authors' contributions

GN designed and carried out the microarray experiments, performed qRT validation, analyzed the data, and wrote the initial draft of the manuscript. AR annotated the data and wrote sections of the manuscript. RCW helped in the design and carried out the cytological staging, and performed the Western analysis. JFF performed the statistical analysis. WZC and VW conceived of the study, participated in its design and coordination, and helped to edit the manuscript. All authors read, edited, and approved the final manuscript.

## Supplementary Material

Additional file 1 **Ordered K-median (K = 4) hierarchy linkage tree of global transcriptome data among 8 biological sample types: male sterile (S) and fertile (F) *am1-489 *(489) and *am1-praI *(pra) anthers at 1.0 and 1.5 mm stages**. Coph (cophenetic correlation) = 0.9842. Click here for file

Additional file 2 **Heat map of 78 probes spanning 45 meiotic genes on the Agilent 4 × 44 K array**. F = fertile; S = male sterile; ND = not determined. Click here for file

Additional file 3 **Congruence between the pilot array (P) and the reported array (M) studies**. A chart **(a) **showing average intensities as listed in **(b) **of 297 differentially expressed, PMC-enriched genes from the two independent studies. 489S: male sterile *am1-489*; praS: male sterile *am1-praI*; 1.0: 1.0 mm anthers; 1.5: 1.5 mm anthers. B. Click here for file

Additional file 4 **Quantitative RT-PCR (qRT) validation of the levels of *Am1 *and 10 other meiotic genes in anthers: male sterile (S) and fertile (F) *am1-489 *(489) and *am1-praI *(pra) anthers at 1.0 and 1.5 mm stages**. Standard deviations are indicated for the qRT results. Click here for file

Additional file 5 **Western analysis of total proteins extracted from tassel branches containing anthers ranging from 1.0 to 3.0 mm in length, collected from homozygous *am1-489 *mutant (*am1-489*), homozygous *am1-praI *mutant (*am1-praI*), and wild type (WT) plants**. Polyclonal antibodies raised to the AM1 protein and specific to actin (control) are described in Pawlowski et al. (10). Click here for file

Additional file 6 **Gene counts of GO-annotated transcripts of: total detected transcripts (all); differentially expressed in *am1-489 *and *am1-praI *anthers compared to common fertile dataset at 1.0 mm (489_1.0, pra_1.0) and 1.5 mm (489_1.5, pra_1.5), respectively; differentially expressed in both mutants compared to fertile at 1.0 mm (489+pra_1.0) and 1.5 mm (489+pra_1.5)**. Click here for file

Additional file 7 **List of 297 PMC-enriched genes differentially expressed in both am1-489 and am1-praI mutants**. Click here for file

Additional file 8 **List of 37 PMC-enriched genes clustered with the *Am1 *transcript expression pattern as in Figure **6. Highlighted items are genes previously reported to be associated with meiosis or meiosis-related processes; ND: not determined. Click here for file

Additional file 9 **Heat map and annotation of the six transcripts most similarly regulated with the *Ameiotic1 *gene**. F = fertile; S = male sterile; ND = not determined. Click here for file

Additional file 10 **List of 45 PMC-enriched genes clustered with the *Skp1B *transcript expression pattern as in Figure **7. Highlighted items are genes previously reported to be associated with meiosis or meiosis-related processes. ND: not determined. Click here for file

Additional file 11 **Primer sequences and RT product sizes of all genes surveyed in qRT validation experiments**. Click here for file
