# Supplementary figures and images for: Global transcriptome analysis of two ameiotic1 alleles in maize anthers: defining steps in meiotic entry and progression through prophase I
Source: BMC Plant Biol. 2011 Aug 26;11:120. doi: 10.1186/1471-2229-11-120 (PMC3180651; doi:10.1186/1471-2229-11-120)

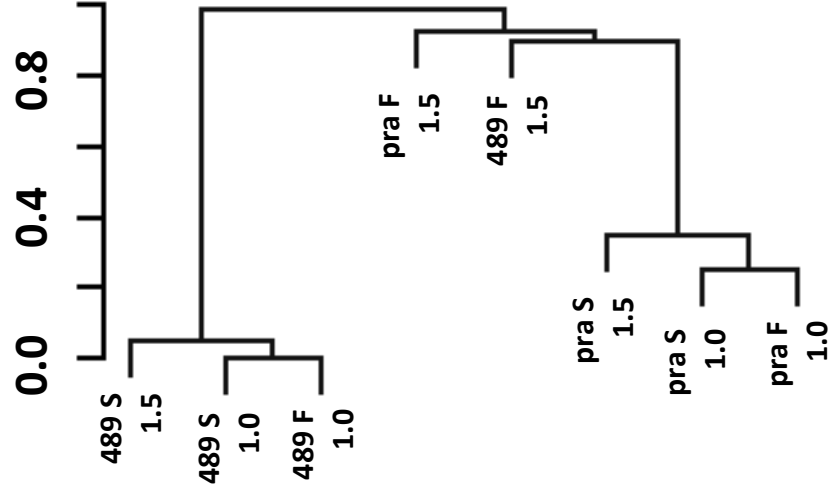

Supplement: Additional file 1 — Ordered K-median (K = 4) hierarchy linkage tree of global transcriptome data among 8 biological sample types: male sterile (S) and fertile (F) am1-489 (489) and am1-praI (pra) anthers at 1.0 and 1.5 mm stages. Coph (cophenetic correlation) = 0.9842. [file 1471-2229-11-120-S1.PDF]

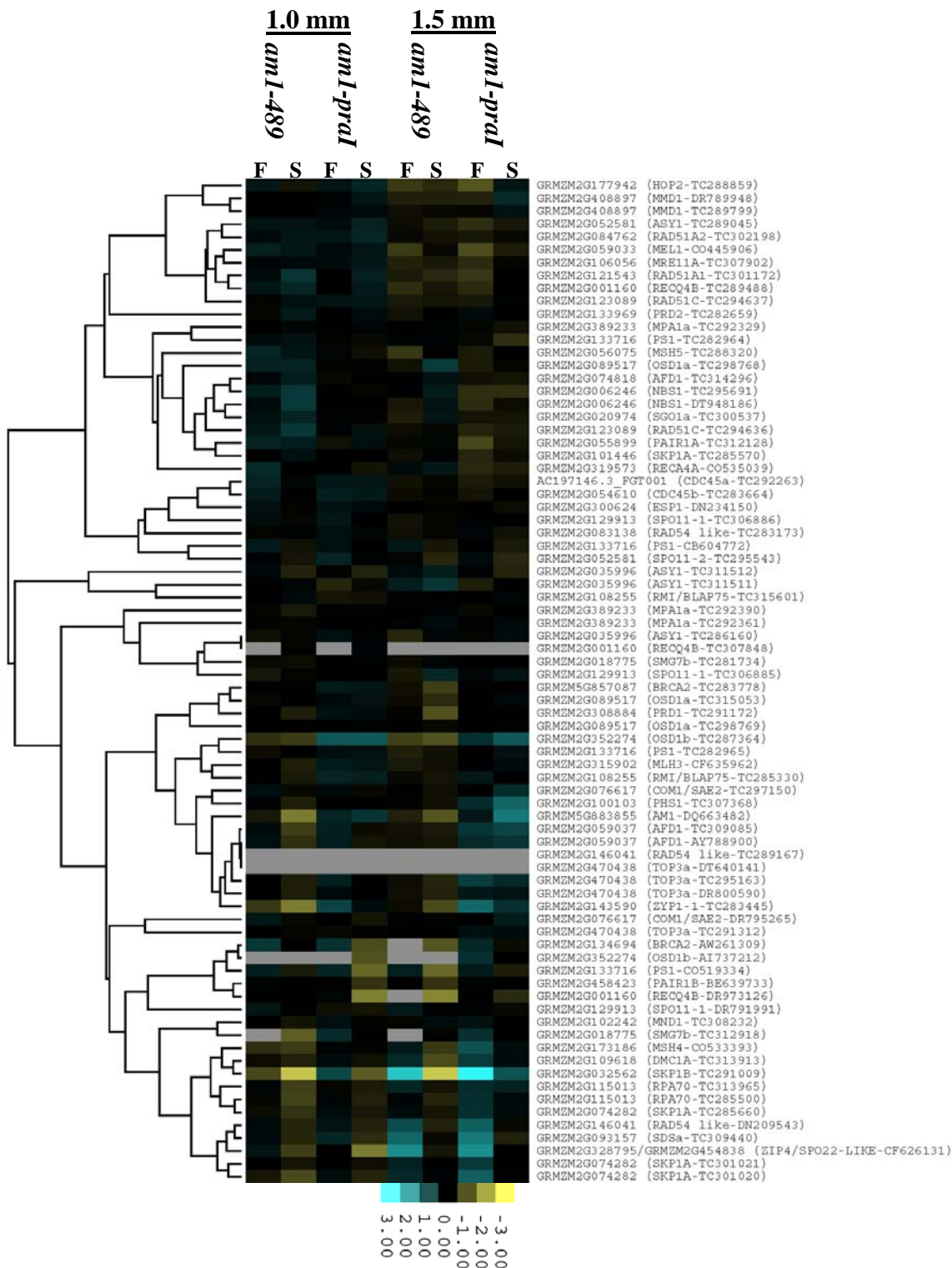

Supplement: Additional file 2 — Heat map of 78 probes spanning 45 meiotic genes on the Agilent 4 × 44 K array. F = fertile; S = male sterile; ND = not determined. [file 1471-2229-11-120-S2.PDF]

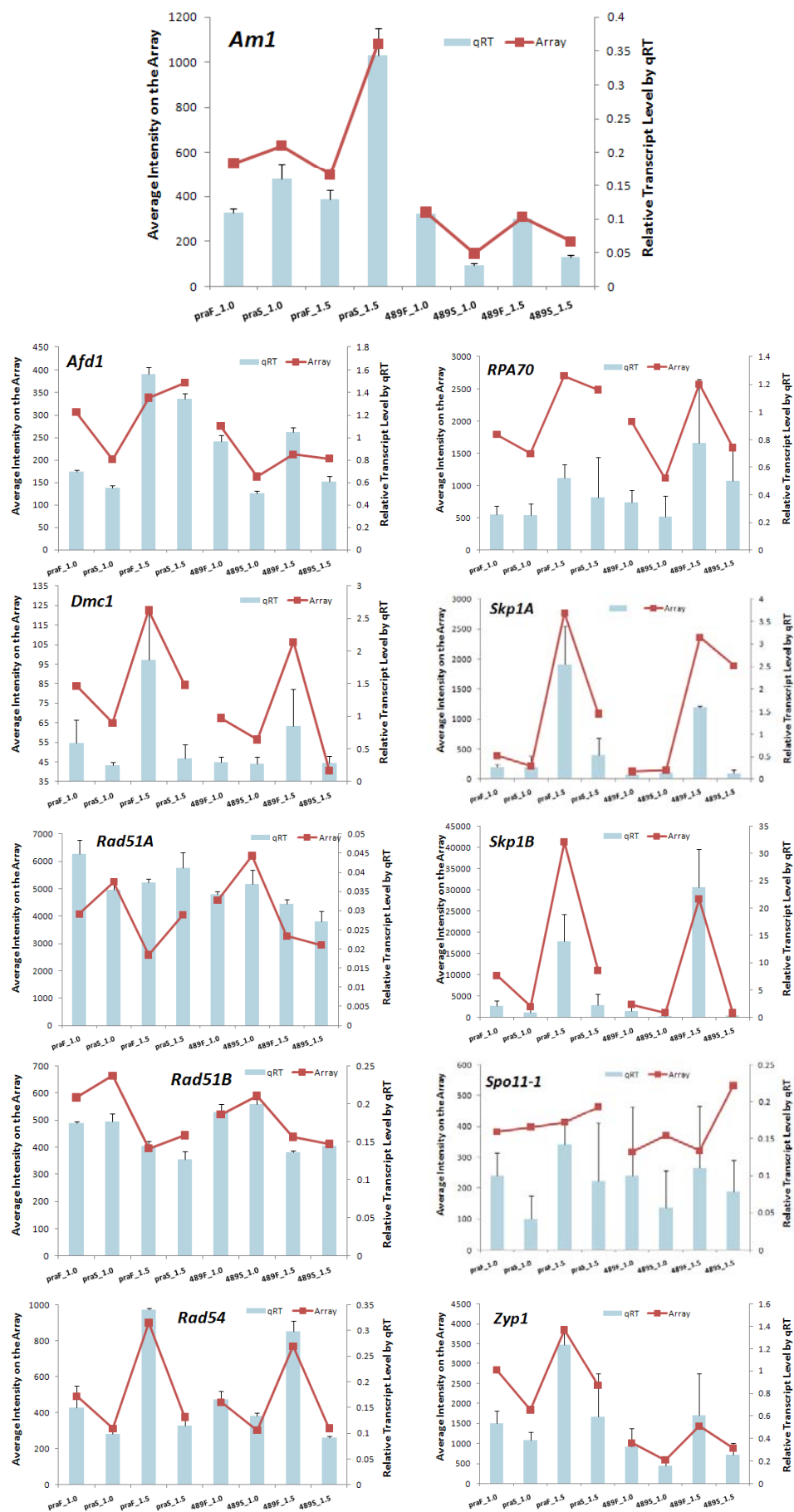

Supplement: Additional file 4 — Quantitative RT-PCR (qRT) validation of the levels of Am1 and 10 other meiotic genes in anthers: male sterile (S) and fertile (F) am1-489 (489) and am1-praI (pra) anthers at 1.0 and 1.5 mm stages. Standard deviations are indicated for the qRT results. [file 1471-2229-11-120-S4.PDF]

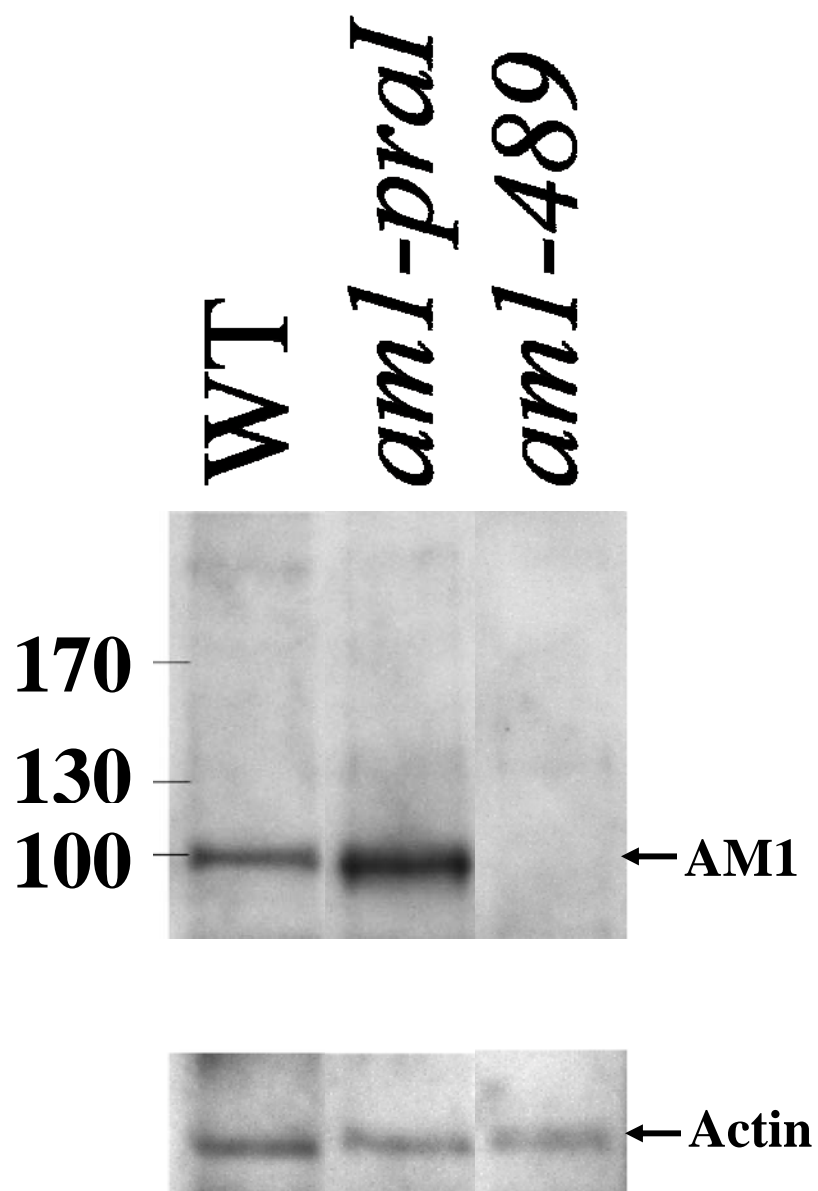

Supplement: Additional file 5 — Western analysis of total proteins extracted from tassel branches containing anthers ranging from 1.0 to 3.0 mm in length, collected from homozygous am1-489 mutant (am1-489), homozygous am1-praI mutant (am1-praI), and wild type (WT) plants. Polyclonal antibodies raised to the AM1 protein and specific to actin (control) are described in Pawlowski et al. (10). [file 1471-2229-11-120-S5.PDF]
