# Supplementary material for: Global transcriptome analysis of two ameiotic1 alleles in maize anthers: defining steps in meiotic entry and progression through prophase I
Source: BMC Plant Biol. 2011 Aug 26;11:120. doi: 10.1186/1471-2229-11-120 (PMC3180651; doi:10.1186/1471-2229-11-120)
Supplement: Additional file 3 — Congruence between the pilot array (P) and the reported array (M) studies. A chart (a) showing average intensities as listed in (b) of 297 differentially expressed, PMC-enriched genes from the two independent studies. 489S: male sterile am1-489; praS: male sterile am1-praI; 1.0: 1.0 mm anthers; 1.5: 1.5 mm anthers. B. [file 1471-2229-11-120-S3.PDF]

(a)

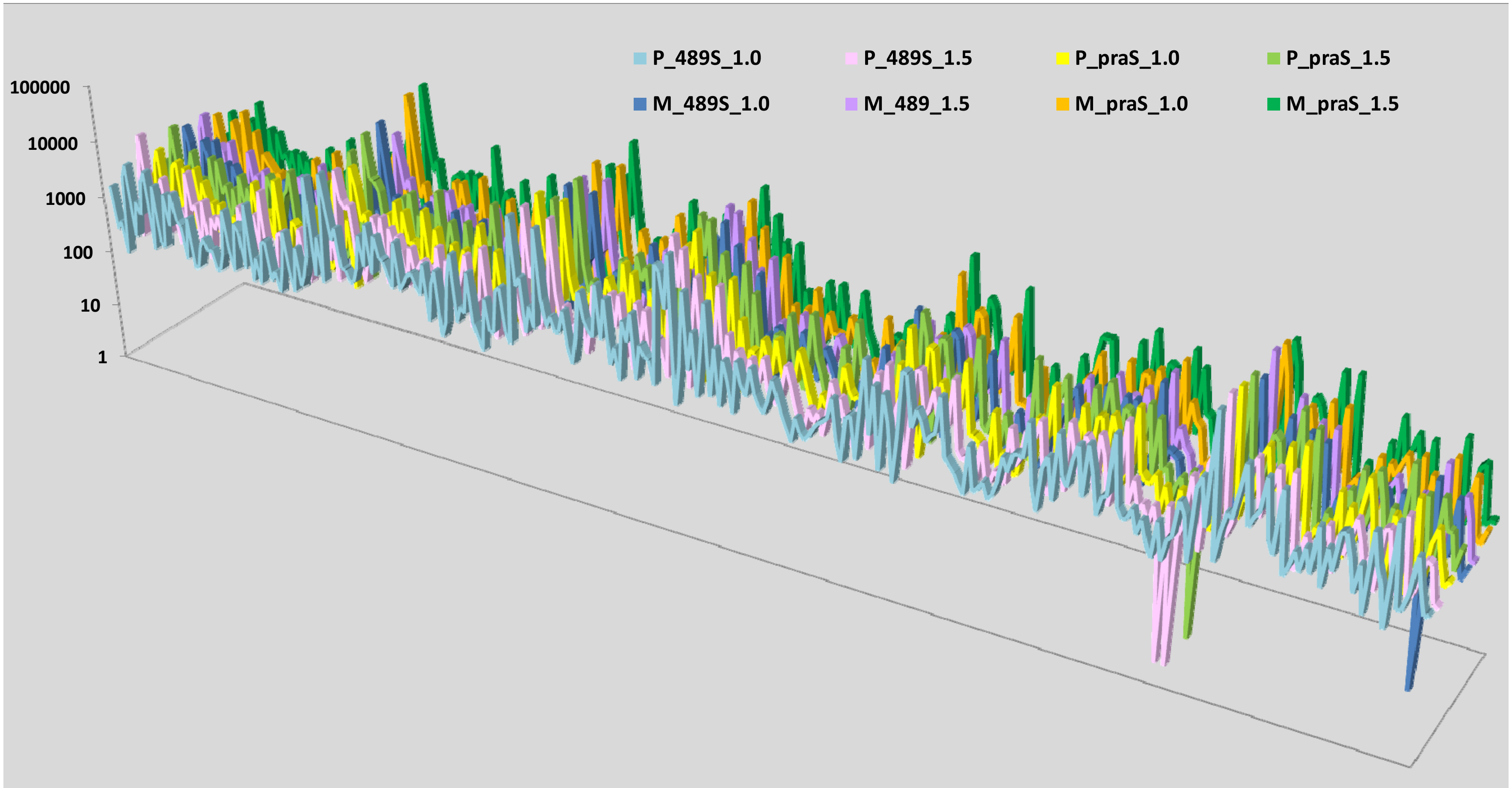

(b)

GeneName	P_489S_1.0	P_489S_1.5	P_praS_1.0	P_praS_1.5	M_489S_1.0	M_489S_1.5	M_praS_1.0	M_praS_1.5
AI692111	1422.5	900.3	1268.4	696.9	1185.4	710.7	813.2	379.2
AI944295	257.9	442.8	267.5	317.0	138.6	227.0	172.6	313.6
AM1	348.5	357.2	1193.1	1489.4	146.6	200.5	630.8	1081.1
AW231811	101.0	141.5	152.8	417.5	63.8	90.1	102.9	183.1
BG319836	2532.2	1666.9	2136.2	1743.8	4823.5	3175.4	3986.1	3137.7
BG319898	4198.7	10125.9	3852.8	7024.9	2611.9	5840.5	1169.4	528.9
BG837957	227.3	241.7	226.1	237.2	112.8	107.6	150.8	126.0
BG841754	983.1	658.0	888.4	576.7	693.0	623.7	462.6	300.0

BM259506	612.3	796.9	562.1	741.2	401.7	427.3	182.8	221.2
BM340065	2442.3	1205.2	2212.3	1367.3	3310.0	1944.3	3658.1	2651.5
BM378145	3909.5	2065.5	2840.7	3060.6	1849.9	1479.4	1222.8	2588.2
BM500607	170.3	219.3	185.9	447.1	65.2	126.6	70.0	121.7
BQ163730	2376.7	1190.2	2378.7	1075.1	3677.4	2463.3	6203.4	6213.5
CA827264	234.1	400.9	273.0	380.1	163.3	279.4	180.3	219.9
CB278279	209.9	166.9	203.6	178.7	102.7	99.7	116.6	148.8
CB280793	1878.3	729.2	2626.1	2892.2	1594.4	633.6	2953.0	2345.6
CD436448	536.7	346.6	508.0	239.0	162.0	200.9	238.2	189.2
CD447985	2081.0	3484.9	1625.6	2782.8	1560.1	1955.7	1240.4	2114.1
CD573220	725.8	778.9	614.0	726.5	927.8	582.8	906.2	732.5
CD995221	604.3	992.9	737.2	1089.9	520.5	791.2	704.5	967.9
CD995946	168.2	140.0	227.4	193.6	216.5	99.9	555.3	534.3
CF019406	924.7	1271.9	756.6	1023.8	831.4	1039.2	658.8	1141.1
CF040072	121.8	124.9	128.2	126.4	103.9	128.5	142.9	177.4
CF059625	135.0	703.7	195.8	1938.2	146.2	647.5	250.5	1126.8
CF626131	321.5	439.0	198.8	464.7	240.7	330.7	145.2	398.0
CF629011	455.0	804.1	551.6	1259.3	391.2	435.0	583.8	888.8
CF633046	285.4	251.9	244.7	201.8	195.9	156.1	206.3	174.0
CF635716	321.1	244.4	305.8	213.5	372.3	338.3	447.4	427.3
CK787298	149.3	182.7	147.1	203.4	70.7	96.5	69.0	96.8
CN844996	157.4	213.4	163.7	254.1	102.6	73.6	155.3	188.7
CO440202	307.1	199.4	732.3	461.1	94.9	128.1	393.8	346.0
CO441573	1966.0	1592.7	2919.7	2737.5	1560.6	1233.5	1752.9	1930.5
CO526721	395.3	373.0	437.4	1536.1	304.1	209.8	261.1	747.0
CO533393	181.3	204.8	239.8	324.1	87.9	91.0	139.8	164.0
CX725290	1288.2	466.2	1008.7	609.7	850.9	422.4	1060.1	487.0
DN559761	957.2	1359.3	819.9	1405.6	519.2	780.0	439.5	841.0
DN586214	233.9	341.2	355.8	1835.9	103.5	171.9	121.7	405.8
DR795221	3261.3	3850.0	4235.7	4143.4	2108.6	2515.1	3010.4	3575.2
DR813132	151.0	153.9	172.0	191.0	61.1	74.2	72.3	73.1
DR829208	389.8	336.4	394.6	387.3	253.3	279.0	253.4	401.5
DR830496	147.5	107.9	193.0	182.9	75.2	72.3	81.7	108.8
DR906542	812.6	771.5	913.2	1288.3	615.6	523.4	666.0	836.8
DT643307	889.0	855.6	760.5	969.8	572.3	474.4	691.7	626.0
DT645987	196.6	218.7	195.9	303.7	84.0	106.8	118.1	110.2
DT647408	272.5	252.5	349.7	407.7	132.2	166.5	215.1	307.5
DT647788	128.1	129.6	139.6	220.4	61.3	79.8	78.0	137.4
DT650280	344.9	384.1	195.8	151.6	475.8	402.1	60.5	40.8
DT652253	1639.6	1243.3	1945.3	3980.8	712.2	571.1	944.3	3041.3
DT943053	330.7	389.6	330.4	450.5	268.6	252.8	281.8	281.2
DT943054	161.9	163.9	152.0	176.1	96.6	94.7	104.0	104.6

DT943243	993.5	532.7	944.4	576.2	817.2	517.4	699.7	731.9
DT943270	215.3	238.8	191.0	212.3	94.0	154.5	81.8	80.0
DT946613	350.8	351.5	258.1	311.1	200.2	214.9	156.0	147.6
TC279480	793.8	869.9	6798.5	7181.7	787.0	973.8	4333.0	5483.4
TC279550	21353.7	12033.7	12114.6	19541.9	10926.1	7495.8	7140.2	10018.3
TC279560	403.1	496.4	575.0	572.0	373.8	383.7	639.0	691.4
TC279580	1984.9	1839.5	1857.7	2042.6	1336.8	945.2	1548.9	951.5
TC279657	346.0	248.5	120.4	118.4	371.8	216.1	97.4	87.6
TC279806	28364.7	22327.6	17579.1	46059.9	51924.7	22606.6	78024.7	82862.8
TC279890	8063.7	6820.7	8623.3	8305.2	9306.5	8488.1	9077.6	10200.9
TC280195	2160.6	5203.4	2124.1	7644.2	1361.9	2582.7	1227.9	2674.0
TC280500	3594.3	6147.5	3048.7	5088.9	2820.9	4012.0	2303.4	4100.7
TC280737	743.3	821.8	830.0	943.0	458.5	597.4	415.5	417.1
TC280740	708.3	743.8	722.4	765.4	432.3	546.3	384.5	461.5
TC280797	492.4	318.3	654.1	456.5	286.9	219.1	554.8	576.5
TC280985	500.0	772.4	2125.5	3930.2	184.5	326.1	988.5	2510.4
TC281079	872.2	962.6	2233.9	3026.7	817.0	803.7	1799.6	2856.9
TC281453	3465.6	5084.0	3883.3	5120.7	2150.6	2564.6	1943.1	2436.1
TC281589	1037.5	1460.4	1165.3	1407.3	591.7	1146.1	694.4	1226.5
TC282058	5894.7	5502.8	6937.5	6729.8	2902.3	3235.1	3540.0	3463.3
TC282176	857.4	411.6	784.3	482.1	841.3	526.0	986.5	790.6
TC282507	4061.4	3774.5	5194.2	3007.9	2245.8	3625.7	3692.9	3504.2
TC282818	1781.6	1854.1	1450.4	1546.1	901.1	1052.2	860.2	960.3
TC282918	1613.3	2951.3	1641.4	3118.9	879.6	1526.7	714.6	856.0
TC282924	971.9	1749.4	1267.7	1705.3	916.0	2081.7	1039.4	2137.6
TC283041	630.4	455.5	546.2	373.6	401.2	348.2	334.1	244.2
TC283097	4043.3	2163.2	6724.8	9548.2	2879.7	2465.2	5476.7	13487.9
TC283173	631.6	744.4	793.8	741.9	302.6	313.4	312.5	375.9
TC283431	838.9	1075.7	988.6	1216.1	682.1	889.7	952.2	1588.1
TC283445	1222.1	1450.6	3532.9	3233.9	587.4	887.3	1844.2	2457.6
TC283469	1372.7	1014.5	925.4	695.2	1622.3	1594.7	906.8	880.1
TC283544	867.7	957.8	881.6	880.6	428.6	454.2	449.2	503.7
TC283684	664.0	624.3	685.6	673.0	388.9	307.0	395.3	325.6
TC283691	2236.7	3076.4	2324.3	3736.7	2689.6	2862.1	2924.5	4425.9
TC283769	252.7	270.8	232.3	281.1	177.3	171.9	157.7	188.2
TC283790	1123.7	1938.4	1153.8	1710.6	398.6	740.4	493.5	819.4
TC283852	1968.4	1349.7	2144.5	1770.6	1223.4	1132.2	1327.1	1537.6
TC283905	153.3	214.8	169.4	452.4	72.9	84.7	76.0	130.4
TC284035	270.5	710.2	816.4	2452.7	104.0	405.9	404.9	994.3
TC284042	1642.5	1353.8	1929.1	1982.7	684.5	626.7	1435.2	1219.9
TC284111	4701.6	3048.7	6775.1	10629.5	2401.1	2521.2	3577.0	7532.9
TC284146	337.3	178.0	493.5	351.2	211.2	130.6	329.6	296.1

TC284163	612.5	525.4	713.6	515.4	468.3	409.9	590.0	573.8
TC284316	517.7	613.2	519.0	664.1	383.5	417.4	443.7	593.8
TC284424	2681.1	4856.0	2861.0	4868.9	2457.6	3822.7	2666.5	4692.4
TC284496	382.8	421.4	460.2	677.6	359.7	795.5	470.5	710.1
TC284526	225.9	240.5	325.6	314.1	139.4	143.9	157.7	200.3
TC284552	141.2	150.8	166.9	161.5	104.5	90.9	155.0	119.2
TC284637	1123.9	797.5	1343.9	1012.6	773.8	801.8	985.2	1091.0
TC284639	323.0	322.7	349.2	520.3	259.7	223.6	321.7	507.5
TC284770	338.2	308.3	431.2	286.4	175.8	165.5	234.9	197.6
TC284771	1857.9	2271.0	1858.9	2466.0	1343.7	1135.6	1279.7	1138.7
TC285165	2185.7	1526.8	2662.3	2199.6	1122.3	926.7	1432.4	1736.7
TC285351	395.8	429.8	737.7	2408.2	317.4	503.8	639.1	2426.5
TC285412	264.8	241.9	299.0	241.4	179.2	261.1	204.5	183.1
TC285655	42646.9	41597.7	46357.1	25491.8	30625.3	28153.5	35126.5	21508.0
TC286055	2803.1	2632.9	4793.7	2934.5	2125.3	2227.6	3496.1	3404.6
TC286409	4105.5	4571.2	3453.7	3461.3	3424.8	4003.0	2272.6	3355.3
TC286486	480.8	388.7	468.2	505.4	489.0	520.4	432.8	613.4
TC286746	1403.4	1381.9	5966.3	5447.8	1587.9	1562.2	6173.7	17016.7
TC287318	647.1	681.9	812.5	1022.3	2083.3	2002.8	2859.9	3556.1
TC287319	33682.1	33319.3	44591.6	72911.4	27970.2	32621.7	38776.4	73353.2
TC287640	2272.1	1795.9	2439.5	2433.4	1463.9	1460.9	1384.5	1713.1
TC287642	798.0	540.8	959.6	888.5	342.3	308.5	393.2	335.3
TC287674	1220.9	1206.0	1539.1	1474.2	555.6	679.6	512.7	753.1
TC287826	719.0	291.9	589.5	390.1	549.0	319.2	393.2	322.1
TC287858	746.5	592.7	986.3	983.6	1514.2	526.8	3646.6	1726.2
TC287864	1755.4	740.9	1562.9	744.9	1447.4	640.5	1259.1	789.5
TC288463	439.2	1200.7	653.3	1031.7	278.6	596.9	536.7	1264.2
TC288590	248.1	244.7	471.1	1240.9	217.1	278.9	557.9	1241.8
TC288800	687.0	696.0	941.6	986.7	697.5	933.3	746.4	962.0
TC289172	4496.6	3788.4	3445.1	4667.7	2549.6	2586.7	3250.6	3319.3
TC289341	1571.8	2257.1	1716.2	3671.2	958.3	1340.5	971.3	1037.1
TC289354	1073.4	2316.3	1252.8	2184.0	1044.5	1475.2	1293.3	1757.5
TC289387	869.7	879.2	848.8	790.1	753.3	924.6	808.3	929.7
TC289458	7315.1	3352.9	7334.5	6828.2	6926.3	3687.2	10216.5	11870.7
TC289461	902.8	963.1	824.7	2666.7	1275.3	678.4	1246.5	1293.5
TC289712	4462.4	3425.2	5412.1	4547.8	1874.2	2530.1	3486.0	3205.8
TC289727	377.1	307.9	426.2	352.9	268.9	303.8	357.4	329.1
TC289753	736.7	1124.9	887.9	1931.9	668.6	1059.1	922.8	1829.5
TC289757	222.1	217.9	276.0	253.3	254.3	244.6	450.4	474.5
TC289774	2319.4	3020.0	3299.5	3790.6	1820.0	2096.4	2080.4	2049.4
TC290304	1068.5	656.2	1003.0	768.2	916.6	504.5	630.2	427.7
TC290471	3436.8	2521.8	5986.9	14337.0	3166.1	1976.1	5125.7	10516.6

TC290945	282.4	228.8	218.4	212.7	152.1	133.4	116.8	122.2
TC291009	1070.7	916.8	1507.2	3998.2	1101.7	1059.9	2562.5	10983.7
TC291467	752.2	872.9	543.7	496.1	651.0	810.6	506.6	569.2
TC291853	686.3	534.1	581.1	536.2	792.0	518.0	666.4	734.6
TC292021	273.9	242.8	262.6	236.5	402.7	220.7	400.9	261.8
TC292121	30288.2	20067.5	4717.5	2885.8	16844.9	13034.7	2748.9	1911.1
TC292342	24050.4	62767.7	30024.6	65242.6	14656.6	41328.6	10997.1	6764.7
TC292387	6033.9	9230.9	12728.1	14067.8	2558.4	3279.7	2955.3	6662.4
TC292774	50614.3	41302.9	54975.1	56806.2	35679.5	35343.1	37322.1	44522.1
TC293138	155.9	201.6	160.8	217.0	113.2	125.5	129.4	126.1
TC293183	763.8	2151.0	929.7	4158.2	497.2	1230.2	685.2	1076.2
TC293263	14132.6	16108.6	11737.0	17842.3	16434.9	13991.3	14707.5	16648.5
TC293287	716.2	981.6	695.9	1518.1	406.0	576.6	378.6	435.7
TC293448	3182.2	4178.5	3878.0	5545.9	2907.1	4624.6	2815.1	6454.1
TC293449	282.7	243.6	316.2	318.6	127.5	147.5	144.2	221.0
TC293566	1601.6	1355.2	1378.8	1414.8	1519.3	1482.9	1150.3	1421.6
TC293567	11315.9	14586.9	12724.8	15125.4	6618.3	8254.6	6065.6	6697.8
TC294126	338.2	408.1	158.2	175.1	151.2	168.4	78.1	74.9
TC294269	2233.9	2422.7	2866.7	5261.8	696.1	757.1	950.7	1140.2
TC294308	247.4	254.9	297.5	358.3	115.8	139.5	147.2	147.8
TC294408	1543.9	1454.8	987.5	831.7	1000.9	778.4	576.8	528.1
TC294630	736.5	902.3	917.0	686.4	633.1	577.1	974.8	860.5
TC294651	221.3	465.9	457.0	579.8	109.1	197.6	148.6	163.4
TC295047	1741.2	1247.4	1674.3	1341.0	1750.2	1292.3	2265.9	2024.0
TC295182	767.3	490.7	836.0	968.0	454.6	344.5	372.4	426.1
TC295193	516.8	582.2	481.7	540.5	343.2	298.5	316.5	233.7
TC295239	1555.8	1475.5	1916.7	3351.7	1094.9	1176.4	1003.3	2109.6
TC295259	599.2	442.4	600.8	525.7	493.6	343.6	478.9	333.8
TC295272	389.0	332.1	356.6	395.5	525.3	351.6	499.9	571.7
TC295587	322.4	285.3	598.3	218.5	197.3	177.2	452.4	205.1
TC295697	540.2	531.4	446.6	547.8	353.7	312.4	323.6	362.8
TC295705	1265.1	1428.6	1637.8	4405.4	733.3	780.1	1102.1	1934.1
TC295868	715.7	772.1	871.3	870.7	481.4	496.4	572.9	419.3
TC295884	368.1	329.1	394.2	462.3	281.0	234.2	292.0	258.1
TC295891	226.2	171.6	186.0	188.3	132.9	111.6	142.4	116.6
TC295938	140.7	135.1	116.3	112.4	89.9	91.8	100.6	90.0
TC296050	378.2	341.0	387.6	352.2	185.9	173.4	255.5	178.7
TC296253	221.3	219.3	242.1	402.2	111.5	106.2	134.2	206.2
TC296255	180.1	326.3	449.5	1687.6	105.2	170.1	234.5	465.6
TC296658	222.1	142.0	269.3	154.2	304.1	96.1	1550.4	479.4
TC296799	318.3	345.3	367.2	467.0	202.7	208.2	241.4	232.3
TC296831	403.6	1282.3	1448.6	5257.1	246.7	766.0	466.4	938.3

TC296845	455.3	620.4	623.2	1083.8	357.9	323.0	657.1	880.9
TC297030	543.0	735.7	453.6	922.8	331.9	700.3	287.9	429.4
TC297071	1219.3	302.6	541.4	329.5	1478.4	295.4	645.9	423.8
TC297465	248.3	155.0	461.1	185.5	151.5	132.4	421.6	264.0
TC297564	117.3	130.5	120.7	155.1	62.4	66.3	63.8	55.8
TC297828	678.0	645.7	330.8	319.8	500.2	496.9	226.8	251.3
TC297993	787.4	621.1	865.2	703.0	558.5	501.5	674.4	607.8
TC298179	150.6	144.6	237.0	275.3	88.5	125.1	82.5	84.4
TC298200	1135.6	1696.1	1767.3	3324.9	553.8	785.7	618.6	2030.5
TC298303	7817.1	2829.1	1281.1	764.0	9190.6	3566.3	1417.8	979.6
TC298797	448.4	309.3	494.3	380.3	241.4	245.1	267.8	365.8
TC298798	3175.4	4503.2	2989.4	6305.6	1514.1	2150.6	1536.9	2690.5
TC299289	128.3	176.2	111.2	133.1	115.0	132.3	116.5	101.7
TC299943	2542.4	908.0	12787.3	15390.3	1601.9	836.6	18444.3	25891.8
TC300898	897.4	1561.9	1389.2	5921.2	546.0	602.6	599.2	679.2
TC300972	94.9	99.9	98.8	109.8	85.9	97.1	109.6	111.3
TC301331	530.8	665.0	437.5	698.8	412.6	630.6	412.6	843.0
TC301356	7253.8	4715.5	7460.9	6417.2	5823.4	4502.0	5556.5	5968.1
TC301395	6849.9	5174.8	7038.5	6498.9	4450.6	3306.4	4465.5	4176.2
TC301402	972.5	944.2	974.9	1176.7	656.1	592.0	799.3	803.1
TC301446	1986.0	1278.8	2377.9	1850.3	853.6	722.3	1018.0	825.2
TC301530	1398.6	2796.6	835.4	1651.2	434.2	937.4	612.8	613.4
TC301734	632.2	885.9	839.7	1071.2	245.7	471.9	291.9	404.2
TC301790	866.3	875.5	926.6	1742.8	762.9	686.9	892.5	1042.3
TC302041	1020.3	858.9	1001.3	1155.8	912.5	580.7	731.5	453.2
TC302095	4259.9	5327.0	6609.2	9821.5	3704.6	3903.4	6525.4	12149.2
TC302216	459.4	371.8	428.9	400.5	221.1	213.9	241.5	196.6
TC302598	345.9	276.7	425.7	308.2	173.7	152.1	248.9	184.5
TC302617	275.3	318.4	346.0	642.3	185.8	246.5	227.7	227.2
TC302695	189.5	168.7	195.8	217.3	98.0	141.2	157.3	107.5
TC302844	134.5	138.0	170.0	205.9	79.9	67.8	99.1	89.3
TC302888	910.2	589.5	1337.1	763.5	530.4	631.6	1131.9	844.4
TC303407	167.9	152.8	150.1	165.7	118.5	97.6	135.6	118.6
TC303479	201.2	164.2	304.2	357.6	126.0	99.9	149.1	138.8
TC303615	267.3	255.3	300.7	281.4	156.0	118.1	131.8	126.3
TC303749	148.1	183.4	145.6	249.5	67.5	92.1	72.3	87.0
TC304232	186.0	180.4	159.4	176.1	91.4	96.0	81.4	84.7
TC304331	323.8	1556.5	501.5	8657.5	91.3	565.2	138.7	1674.4
TC304530	881.0	386.5	838.7	429.5	579.2	279.4	596.3	318.2
TC304557	514.2	545.9	227.0	302.4	322.0	393.9	107.1	201.3
TC304579	1074.1	1575.2	1710.9	2349.9	717.7	1048.4	1232.4	1861.3
TC305157	1147.4	1150.7	1320.0	3459.6	1192.8	1038.7	1440.0	4223.9

TC305158	1035.5	1388.9	866.5	2566.5	1669.0	1330.7	1680.9	4088.5
TC305266	4228.7	2803.0	6684.8	5760.5	2571.8	2344.0	3692.9	4206.2
TC305399	407.3	279.3	385.0	360.2	399.1	238.7	410.2	393.0
TC305717	160.6	187.5	271.2	335.1	180.2	121.6	161.0	215.8
TC305979	869.9	679.1	1042.3	933.6	810.7	636.0	795.4	674.8
TC306026	1431.7	1585.7	1605.7	2002.1	645.1	812.1	735.8	1147.0
TC306070	287.0	261.7	369.9	395.1	181.9	148.6	247.7	199.9
TC306072	3844.2	3687.3	4122.3	5387.0	3759.3	3599.6	3925.8	5042.5
TC306103	802.5	1198.6	743.5	775.7	707.7	1164.5	415.5	415.2
TC306328	573.5	583.2	723.6	1863.6	321.3	243.6	240.5	755.0
TC306331	1686.5	2542.8	3269.9	7912.8	1575.6	1894.9	2790.6	9027.1
TC306547	3005.3	1870.6	3465.3	2384.2	3027.6	1759.6	2837.1	1260.1
TC306976	468.8	371.3	464.5	466.3	620.3	565.3	579.5	848.1
TC307255	3460.8	3140.0	4145.6	3343.9	2967.7	2945.2	3857.2	3135.9
TC307363	1278.1	1551.1	1923.6	3056.5	862.0	905.7	1457.0	2792.2
TC307437	277.8	244.0	316.2	274.9	127.8	148.0	172.3	258.9
TC307549	570.6	706.4	671.7	1021.2	396.1	455.5	520.8	925.7
TC307556	638.1	789.7	739.3	1184.6	297.4	362.2	368.2	508.2
TC307673	6722.8	6886.3	7192.5	5190.6	7611.4	6966.7	7000.3	6686.9
TC307873	452.2	325.7	636.8	377.2	335.0	276.8	392.2	284.8
TC307982	321.3	839.5	500.8	3327.5	648.7	921.1	1590.6	3723.1
TC307997	342.3	279.6	493.4	446.4	580.2	487.0	800.8	699.2
TC308047	507.2	380.5	702.7	588.2	485.8	370.5	647.9	622.8
TC308051	210.5	188.0	166.4	149.6	119.9	130.9	144.1	162.0
TC308341	379.8	393.2	512.5	334.4	196.2	234.0	199.0	310.1
TC308574	172.4	148.9	195.5	469.6	99.5	120.6	136.9	291.7
TC308593	103.6	BelowBG	98.2	98.2	69.3	57.6	78.7	83.8
TC308668	421.2	462.3	532.1	643.4	265.2	299.1	250.7	266.0
TC308672	100.2	BelowBG	104.0	BelowBG	324.3	53.4	779.3	821.5
TC309174	236.3	166.7	354.4	198.1	116.7	97.4	376.5	205.7
TC309440	285.1	320.9	286.6	289.9	141.7	178.1	133.5	145.7
TC309689	1130.7	1771.5	1214.7	2044.9	631.2	844.3	507.3	627.6
TC309747	729.8	581.3	740.2	821.3	487.6	439.0	621.1	916.7
TC309808	124.4	116.1	158.5	190.1	65.5	87.3	107.7	120.7
TC309875	2220.2	3298.2	2771.5	4538.4	1262.4	1819.4	1449.8	2462.6
TC309993	3456.7	5172.5	4076.9	5789.7	2540.7	3427.6	2735.0	3837.8
TC310105	561.8	399.6	572.2	508.9	211.2	161.9	307.9	192.4
TC310187	43425.7	51175.1	42523.7	39692.3	22875.1	40021.0	33297.3	24199.8
TC310318	177.7	276.8	335.4	2045.2	1886.9	518.1	10793.6	9677.4
TC310354	589.5	470.0	799.9	993.3	647.1	355.1	893.9	851.0
TC310367	1220.8	1497.7	666.7	637.9	601.0	851.2	283.6	309.8
TC310683	1029.7	1769.8	1339.8	2323.3	679.0	853.4	890.5	1268.0

TC310688	2925.9	4643.2	4267.2	5991.6	1487.9	2065.1	2467.5	3838.5
TC310843	8544.8	6707.8	6508.5	4821.2	7252.8	9788.6	4327.9	2982.0
TC310988	925.1	802.9	1252.8	948.6	539.0	639.2	1413.9	1325.3
TC311135	1400.9	1721.0	1588.0	2028.8	1399.9	1557.5	1416.2	1637.2
TC311214	1835.8	2602.3	1409.9	1439.8	1176.5	2092.7	532.1	610.8
TC311526	6440.5	5549.3	7970.8	18455.8	5081.8	4734.0	6357.8	12605.5
TC311757	693.5	800.2	852.4	1420.0	456.9	733.4	939.2	1193.9
TC311769	430.1	323.6	505.5	340.9	508.8	404.6	440.2	339.5
TC311848	5003.2	5604.0	9756.7	10887.5	4092.5	4332.0	6027.4	13290.6
TC312091	222.7	164.9	258.1	234.5	140.6	100.6	157.6	104.3
TC312257	325.9	246.8	318.2	246.4	255.1	173.1	402.7	169.3
TC312299	624.1	643.8	547.0	591.8	595.8	536.5	514.0	562.7
TC312497	306.6	242.7	305.4	251.9	189.0	161.1	149.7	159.4
TC312972	784.3	565.0	736.8	665.6	353.9	299.1	439.2	465.7
TC312974	322.7	481.7	309.3	573.4	152.2	200.2	184.7	232.0
TC313063	1935.5	1228.0	2614.0	1720.3	1584.7	822.5	1398.1	1478.8
TC313076	219.6	227.2	239.2	237.6	158.0	166.0	214.6	202.5
TC313084	969.6	920.4	1643.2	3998.9	1064.7	895.0	1237.5	4408.8
TC313491	438.9	491.6	472.1	504.7	828.9	802.8	785.4	951.1
TC313569	1878.3	914.7	1796.4	1124.8	1246.8	938.9	1323.4	1259.9
TC313596	2028.5	2064.0	5613.8	4484.3	1262.8	1354.8	2005.6	2689.7
TC313657	898.4	930.8	1760.4	1602.8	469.0	509.8	746.0	678.3
TC313810	258.0	323.4	261.9	373.2	129.7	188.4	115.2	151.8
TC313835	750.8	628.9	768.5	1368.7	1451.1	821.2	2239.2	2436.8
TC314126	127.8	180.8	133.0	297.3	63.2	77.5	107.4	187.7
TC314264	2834.3	1838.0	1918.9	738.8	1268.2	963.5	1214.7	573.5
TC314427	871.2	1961.9	1149.6	8258.0	489.4	742.5	318.0	522.3
TC314450	446.2	404.4	316.2	499.5	407.3	296.0	381.0	358.1
TC314530	95.8	196.6	119.8	195.2	0.0	110.8	66.2	152.5
TC314544	4944.7	3832.1	4495.0	3011.5	3316.9	3830.5	2899.6	3855.9
TC314580	221.1	219.1	250.8	270.3	134.6	120.0	139.8	155.8
TC314658	228.2	221.5	233.3	206.2	201.3	230.7	297.6	290.1
TC314676	422.4	466.1	1006.0	3308.8	229.4	394.2	627.7	1456.4
TC315034	1809.6	932.6	1809.3	1061.7	1990.5	1375.6	1755.3	1781.3
TC315043	575.5	846.0	575.7	1002.8	309.9	594.9	202.0	191.7
TC315488	231.6	216.5	247.0	267.8	112.8	141.1	157.2	236.2
TC315563	257.6	188.6	316.1	629.6	175.8	134.8	280.2	234.5
