# Supplementary material for: Global transcriptome analysis of two ameiotic1 alleles in maize anthers: defining steps in meiotic entry and progression through prophase I
Source: BMC Plant Biol. 2011 Aug 26;11:120. doi: 10.1186/1471-2229-11-120 (PMC3180651; doi:10.1186/1471-2229-11-120)
Supplement: Additional file 6 — Gene counts of GO-annotated transcripts of: total detected transcripts (all); differentially expressed in am1-489 and am1-praI anthers compared to common fertile dataset at 1.0 mm (489_1.0, pra_1.0) and 1.5 mm (489_1.5, pra_1.5), respectively; differentially expressed in both mutants compared to fertile at 1.0 mm (489+pra_1.0) and 1.5 mm (489+pra_1.5). [file 1471-2229-11-120-S6.PDF]

	All	*489_1.0*	*489_1.5*	*pra_1.0*	*pra_1.5*	*489+pra_1.0*	*489+pra_1.5*
Unknown	24045	501	1436	336	833	66	255
Binding	4280	105	434	95	253	21	75
catalytic activity	2547	93	294	69	190	19	62
RNA binding	2547	10	27	12	39	3	12
hydrolase activity	2324	71	268	57	121	18	36
nucleotide binding	2246	71	253	59	130	20	31
protein binding	1723	47	204	30	88	9	33
transferase activity	1463	33	161	37	83	8	29
DNA binding	1425	44	163	34	59	5	10
transporter activity	1286	43	164	31	91	13	31
kinase activity	1229	34	171	31	69	7	25
structural molecule activity	687	15	65	6	58	1	12
nucleic acid binding	595	12	57	8	49	3	18
molecular_function	484	20	73	14	29	5	7
enzyme regulator activity	427	0	26	1	9	0	2
transcription factor activity	423	12	49	13	17	1	3
transcription regulator activity	392	7	46	4	9	1	2
receptor activity	308	2	36	6	14	0	3
lipid binding	164	4	18	5	9	0	3
signal transducer activity	147	4	17	3	7	0	2
carbohydrate binding	126	5	18	5	4	1	2
translation factor activity	118	5	13	3	9	0	6
motor activity	115	1	9	4	9	1	2
nuclease activity	104	1	7	0	6	0	2
receptor binding	66	0	13	1	2	0	0
Chromatin binding	13	0	0	0	0	0	0
oxygen binding	5	0	1	0	0	0	0
