# Supplementary material for: Global transcriptome analysis of two ameiotic1 alleles in maize anthers: defining steps in meiotic entry and progression through prophase I
Source: BMC Plant Biol. 2011 Aug 26;11:120. doi: 10.1186/1471-2229-11-120 (PMC3180651; doi:10.1186/1471-2229-11-120)
Supplement: Additional file 7 — List of 297 PMC-enriched genes differentially expressed in both am1-489 and am1-praI mutants. [file 1471-2229-11-120-S7.PDF]

List of 297 PMC-enriched genes differentially expressed in both *am1-489* and *am1-praI* mutants.

Maize Est	Maize Gene ID	E-value	Maize Protein	Description	Start	End	Ori	Loc	Start	End	Ori	Score	E-val	%ID	Length	Best *Sorghum bicolor* hit	Best *Oryza sativa* hit	Best *Arabidopsis thaliana* hit
TC287826	GRMZM2G177445	5.80E-27	ACG47241 (491 aa)	TPR-containing protein kinase	1	60	+	Chr:1	294496038	294496097	+	60	5.80E-27	100.0	60	Sb01g002850	LOC_Os03g61010	At5g59010
AI692111	AC198518.3_FG002	5.80E-27	NP_00114967 7 (192 aa)	Sterile Alpha Motif domain family protein	1	60	-	Chr:3	107406443	107406502	+	60	5.80E-27	100.0	60	Sb01g001150	LOC_Os12g29480	ND
TC287642	GRMZM5G840225	5.80E-27	ND	ND	1	60	-	Chr:2	167327559	167327618	+	60	5.80E-27	100.0	60	ND	LOC_Os07g27950	ND
TC307437	GRMZM2G138526	4.10E-19	ND	ND	1	60	-	Chr:3	144926001	144926060	+	48	4.10E-19	95.0	60	ND	ND	ND
TC293567	GRMZM2G103287	5.80E-27	ACG32808 (332 aa)	**Apospory**-associated protein C ; aldose 1-epimerase family protein	1	60	-	Chr:10	70222871	70222930	+	60	5.80E-27	100.0	60	Sb04g005170	LOC_Os08g14330	ND
TC314580	GRMZM2G139657	5.80E-27	NP_00115255 0 (555 aa)	Auxin-independent growth promoter-like protein	1	60	+	Chr:6	88986716	88986775	+	60	5.80E-27	100.0	60	Sb10g027900	LOC_Os06g47290	ND
TC284526	GRMZM2G114192	5.80E-27	NP_00113272 7 (215 aa)	Rho GTPase-activator protein (RhoGAP)	1	60	-	Chr:2	15978126	15978185	+	60	5.80E-27	100.0	60	Sb06g026110	Os04g0577200	At2g46710
TC298179	GRMZM2G052255	1.40E-24	ND	ND	1	60	-	Chr:8	98049917	98049975	+	56	1.40E-24	98.3	60	ND	ND	ND
TC287640	GRMZM2G139941	5.80E-27	ND	ND	1	60	+	Chr:7	44456919	44456978	+	60	5.80E-27	100.0	60	ND	LOC_Os07g27950	ND
TC307873	GRMZM2G005374	5.80E-27	ND	ND	1	60	-	Chr:8	165557441	165557500	+	60	5.80E-27	100.0	60	ND	ND	ND
TC301446	GRMZM2G045970	5.80E-27	NP_00117070 3 (423 aa)	Cytidylyltransferase family protein	1	60	+	Chr:2	150628111	150628170	+	60	5.80E-27	100.0	60	Sb05g001470	LOC_Os12g02820, LOC_Os11g03050	At2g38670 = AtPECT1
DR830496	AC195587.4_FG003	5.80E-27	ND	ND	1	60	+	Chr:6*	78469182	78469241	+	60	5.80E-27	100.0	60	ND	LOC_Os06g06660, LOC_Os06g06670, LOC_Os06g06680, LOC_Os06g06580, LOC_Os06g06710	ND
DQ663482	GRMZM5G883855	5.80E-27	NP_00113953 8 (780 aa)	***Ameiotic1***	1	60	+	Chr:5	16098646	16098705	+	60	5.80E-27	100.0	60	Sb01g013215	LOC_Os03g44760	ND
TC311769	GRMZM2G004422	1.40E-24	NP_00114201 4 (272 aa)	Zinc finger (C3HC4-type RING finger) family protein	1	60	-	Chr:1	21469434	21469493	+	56	1.40E-24	98.3	60	Sb01g043460	LOC_Os03g10890	AtAt1g49850 = AtRHY1a
TC313596	GRMZM2G114192	3.60E-25	NP_00113272 7 (215 aa)	Rho GTPase-activator protein (RhoGAP)	1	60	-	Chr:2	15976638	15976698	+	57	3.60E-25	98.4	61	Sb06g026110	LOC_Os04g48790	At2g46710
DT647788	No annotated gene	5.80E-27	ND	ND	1	60	-	Chr:10 *	7075246	7075305	+	60	5.80E-27	100.0	60	ND	ND	ND
TC289712	GRMZM2G071739	5.80E-27	ACG31674 (291 aa)	Ubiquitin-associated (UBA)/TS-N domain-containing protein	1	60	+	Chr:3	187440397	187440456	+	60	5.80E-27	100.0	60	Sb03g037500	LOC_Os01g59160	At3g56740
TC283097	GRMZM2G050684	5.80E-27	ACG35725 (422 aa)	CBS (cystathionine beta-synthase) domain containing protein	1	60	-	Chr:1	257205749	257205808	+	60	5.80E-27	100.0	60	Sb01g012180	LOC_Os03g47120	At4g33700
TC293449	GRMZM2G138060	5.80E-27	AAB97167 (789 aa)	SU1 isoamylase	1	60	+	Chr:4	41372858	41372917	+	60	5.80E-27	100.0	60	Sb07g027200	LOC_Os08g40930	At2g39930 = AtISA1 8+J56
TC309808	GRMZM2G328780	5.80E-27	NP_00114979 3 (455 aa)	Flavin-containing monooxygenase YUCCA-type	1	60	+	Chr:3	116517874	116517933	+	60	5.80E-27	100.0	60	Sb08g020340	LOC_Os01g12490 (Os01g0224700 = YUCCA3	At1g04180 = At+J57YUCCA9
TC301402	GRMZM2G547542	3.40E-22	ND	ND	1	56	-	Chr:10	106952386	106952441	+	52	3.40E-22	98.2	56	ND	LOC_Os04g31000	ND
TC282176	GRMZM2G047204	5.80E-27	NP_00113176 3 (288 aa)	Unknown protein	1	60	+	Chr:6	74072433	74072492	+	60	5.80E-27	100.0	60	Sb10g003530	LOC_Os06g05720	At4g32960

TC282924	GRMZM2G107495	5.80E-27	ND	ND	1	60	-	Chr:1	67431865	67431924	+	60	5.80E-27	100.0	60	ND	LOC_Os03g27010	ND
TC301331	GRMZM2G153760	1.40E-24	NP_00113697 1 (222 aa)	Galactosyltransferas e	1	60	-	Chr:4	73605449	73605508	+	56	1.40E-24	98.3	60	Sb07g019420	LOC_Os08g29710	At5g53340 = beta-1,3-galactosyltransferase 11/Avr9 elicitor response protein-like
TC308051	GRMZM2G328795	1.40E-24	ND	ND	1	60	-	Chr:3	169115821	169115880	+	56	1.40E-24	98.3	60	ND	LOC_Os01g66690	ND
TC294308	GRMZM2G062761	5.80E-27	ND	ND	1	60	+	Chr:6	165951055	165951114	+	60	5.80E-27	100.0	60	ND	LOC_Os05g50120	ND
TC292774	GRMZM2G365160	5.80E-27	ND	ND	1	60	-	Chr:2	212180355	212180414	+	60	5.80E-27	100.0	60	ND	LOC_Os07g42960	ND
TC300972	GRMZM2G017634	5.80E-27	NP_00114125 9 (251 aa)	Unknown protein	1	60	+	Chr:7	20202461	20202520	+	60	5.80E-27	100.0	60	Sb02g006600	LOC_Os07g12220	At1g55535
TC298200	GRMZM2G035131	2.70E-14	ND	ND	1	17	-	Chr:3* N55	4553993	4554009	+	17	2.70E-14	100.0	17	ND	LOC_Os01g10250	ND
TC296658	GRMZM2G102711	5.80E-27	ND	ND	1	60	+	Chr:5	12233512	12233571	+	60	5.80E-27	100.0	60	ND	LOC_Os06g32760 +>20g	ND
TC296799	GRMZM2G474929	1.40E-24	NP_00114680 4 (150 aa)	Pentatricopeptide (PPR) repeat-containing protein	1	60	-	Chr:2	26934281	26934340	+	56	1.40E-24	98.3	60	Sb06g023080	LOC_Os05g36350	At2g20540
TC289458	No annotated gene	8.6	ND	ND	15	31	+	Chr:3	146966089	146966105	+	17	8.6	100.0	17	ND	ND	ND
TC287864	GRMZM2G037140	6.80E-14	NP_00113155 7 (128 aa)	Actin-depolymerizing factor (ADF)	1	40	-	Chr:6	35896782	35896821	+	40	6.80E-14	100.0	40	Sb09g001500	Os05g0113400 = OsADF7	At5g59890 =AtADF4
TC298797	GRMZM2G032003	5.80E-27	NP_00113074 2 (473 aa)	UTP--glucose-1-phosphate uridylyltransferase	1	60	-	Chr:7	146491700	146491759	+	60	5.80E-27	100.0	60	Sb04g001320	LOC_Os09g38030	At5g17310 =UDPGP 1
TC313569	GRMZM2G166897	5.80E-27	NP_00113059 5 (403 aa)	Unknown protein	1	60	+	Chr:7	138464006	138464065	+	60	5.80E-27	100.0	60	Sb02g029650	LOC_Os09g33780	At3g51980
CO440202	GRMZM2G083394	2.30E-26	ND	ND	1	59	-	Chr:8	15988675	15988733	+	59	2.30E-26	100.0	59	ND	LOC_Os01g07140	ND
TC302617	GRMZM2G039094	5.80E-27	ND	ND	1	60	+	Chr:1	167333104	167333163	+	60	5.80E-27	100.0	60	ND	LOC_Os12g40520	ND
TC315488	GRMZM2G066005	5.80E-27	NP_00114259 8 (356 aa)	Plant calmodulin-binding domain	1	60	-	Chr:3	149249215	149249274	+	60	5.80E-27	100.0	60	Sb03g045870	Os01g0950700	At5g04020
TC292021	GRMZM2G011078	5.60E-24	NP_00115046 0 (313 aa)	Secretory carrier-associated membrane protein (SC3) protein	1	55	-	Chr:1	246409111	246409165	+	55	5.60E-24	100.0	55	Sb01g015740	LOC_Os03g38590	At1g61250
TC297071	GRMZM2G090563	5.80E-27	NP_00115069 2 (409 aa)	Transmembrane receptor	1	60	+	Chr:8	117151069	117151128	+	60	5.80E-27	100.0	60	Sb09g022750	LOC_Os05g38720	At3g09570
TC289172	GRMZM2G047372	5.80E-27	NP_00116855 2 (199 aa)	Unknown protein	1	60	-	Chr:1	269970099	269970158	+	60	5.80E-27	100.0	60	Sb01g009840	LOC_Os03g51160	At3g12030
TC305266	GRMZM2G007300	5.80E-27	NP_00114948 0 (169 aa)	Ubiquitin-conjugating enzyme E2 UBC7	1	60	+	Chr:3	181451131	181451190	+	60	5.80E-27	100.0	60	Sb03g039320	LOC_Os01g62244	At5g59300
TC298303	No annotated gene	5.80E-27	ND	ND	1	60	-	Chr:1	296891037	296891096	+	60	5.80E-27	100.0	60	ND	ND	ND
TC303479	GRMZM2G007791	5.80E-27	NP_00116987 3 (330 aa)	Argonaute protein, similar to AGO2	1	60	-	Chr:2	9971626	9971685	+	60	5.80E-27	100.0	60	Sb06g028510	LOC_Os04g52540 (Os04g0615700 = OsAGO2), LOC_Os04g52550	At1g31280 =AtAGO2
TC308593	GRMZM2G157172	5.80E-27	NP_00116816 3 (804 aa)	Putative oxysterol binding protein	1	60	-	Chr:5	11944527	11944586	+	60	5.80E-27	100.0	60	Sb01g010720	LOC_Os03g49770	At4g08180 = ORP1C
TC301734	AC177897.2_FG002	5.80E-27	NP_00114260 6 (342 aa)	SPX (SYG1/Pho81/XPR1) domain-containing protein / zinc finger (C3HC4-type RING finger) protein-related	1	60	+	Chr:1	260137372	260137431	+	60	5.80E-27	100.0	60	ND	Os03g0650900	At2g38920 = SPX

TC282507	GRMZM2G414569)	1.40E-24	ACG27791 F76(207 aa)	Transmembrane emp24 domain-containing protein 10 precursor	5	60	-	Chr:5	210304427	210304482	+	56	1.40E-24	100.0	56	Sb10g006960	LOC_Os02g52920	At1g69460 = emp24/gp25L/p24 family protein
TC305979	GRMZM2G116427	5.80E-27	NP_001152309 (221 aa)	Unknown protein	1	60	+	Chr:6	84525498	84525557	+	60	5.80E-27	100.0	60	ND	LOC_Os06g49890	At3g51090
TC306547	GRMZM2G105283	5.80E-27	NP_001130109 (393 aa)	Unknown protein	1	60	+	Chr:3	183256978	183257037	+	60	5.80E-27	100.0	60	Sb03g013450	LOC_Os01g61440, LOC_Os01g18160	At3g11150
TC308672	Low matching score	8.6	ND	ND	20	36	+	Chr:5*	175644388	175644404	+	17	8.6	100.0	17	ND	ND	ND
TC310843	GRMZM2G170927	5.80E-27	NP_001146732 (771 aa)	Vacuolar proton pyrophosphatase ; similar to rice OVP1 and Arabidopsis AVP1	1	60	-	Chr:9	94177915	94177974	+	60	5.80E-27	100.0	60	Sb10g025280, Sb04g005710	LOC_Os06g43660 (Os06g0644200 =Ovp1, Os06g0178900 = ovp2)	At1g15690 = AVP1
TC284111	GRMZM2G128771	5.80E-27	ND	Ras-related protein Rab-6A (microtubule-dependent transport pathways through the Golgi)	1	60	+	Chr:2	212321308	212321367	+	60	5.80E-27	100.0	60	Sb02g039720	Os07g0496000	At2g44610
TC299943	GRMZM2G163514	3.60E-25	ND	SUPPRESSOR OF GENE SILENCING 3 (SGS3) homolog (NP_001105989 ; 138aa)	1	60	-	Chr:3	137393184	137393244	+	57	3.60E-25	98.4	61	ND	LOC_Os12g09580, LOC_Os12g09590	ND
TC314658	GRMZM2G447691	5.80E-27	NP_001142657 (177 aa)	Unknown protein	1	60	+	Chr:2	38139298	38139357	+	60	5.80E-27	100.0	60	Sb06g020010	LOC_Os01g14130	ND
DR829208	GRMZM2G020721	1.60E-18	NP_001170173 (391 aa)	Putative O-acetyltransferase	1	24	+	Chr:9*	134300759	134300782	+	24	1.60E-18	100.0	24	Sb01g037160	LOC_Os03g19970	At2g34410
CB278279	GRMZM2G145085	5.80E-27	ND	ND	1	60	+	Chr:9	142297585	142297644	+	60	5.80E-27	100.0	60	ND	LOC_Os03g14720	ND
TC306976	GRMZM2G162968	5.80E-27	NP_001131857 (492 aa)	Heat chock protein similar to CASEIN LYTIC PROTEINASE (clp) - type B	1	60	-	Chr:9	124034275	124034334	+	60	5.80E-27	100.0	60	Sb01g032210	LOC_Os03g31300	At5g15450 = CLPB3
TC297465	GRMZM2G091656	5.80E-27	ND	GRAS family transcription factor similar to SCARECROW-like 26	1	60	+	Chr:4	213371835	213371894	+	60	5.80E-27	100.0	60	Sb05g003820	LOC_Os11g06180	At4g08250 =AtSCL26
TC305157	GRMZM2G159724	5.80E-27	ACG47397 (593 aa)	NADP-dependent malic enzyme	1	60	-	Chr:3	201687018	201687077	+	60	5.80E-27	100.0	60	Sb09g005810	LOC_Os01g52500	At5g11670
CF059625	GRMZM2G149347	5.80E-27	NP_001148404 (318 aa)	Zinc finger CCCH type ; similar to OsC3H39 and AtC3H14	1	60	+	Chr:6	165862220	165862279	+	60	5.80E-27	100.0	60	Sb09g029330	LOC_Os05g50080 (Os05g0576300 = OsC3H39)	At1g66810 = AtC3H14
TC295587	GRMZM5G862467	3.60E-25	NP_001149189 (216 aa)	MPS one binder kinase activator-like 1A ; cell cycle associated protein Mob1-like protein	1	60	-	Chr:1	71145109	71145169	+	57	3.60E-25	98.4	61	Sb01g032780	LOC_Os03g29570, Os10g0396300	At4g19050
TC283469	GRMZM2G169931	5.80E-27	ACG27981 (525 aa)	Nonsense-mediated mRNA decay protein 3, 60S ribosomal export protein NMD3	1	60	+	Chr:1	99130437	99130496	+	60	5.80E-27	100.0	60	ND	ND	At2g03820 = NMD3
TC283041	GRMZM2G089962	2.20E-23	ND	ND	7	60	-	Chr:6	156595621	156595674	+	54	2.20E-23	100.0	54	ND	LOC_Os05g42010	ND
BQ163730	GRMZM2G410710	1.40E-24	NP_001104968 (282 aa)	Prohibitin 3 (lipid raft-associated integral membrane protein)	1	60	-	Chr:5	177824771	177824829	+	56	1.40E-24	98.3	60	Sb04g024070	LOC_Os02g37000	At5g40770 =ATPHB3
TC302216	GRMZM5G849107	5.80E-27	NP_001169155 (490 aa)	Lycopene beta-cyclase	1	60	-	Chr:5	100700619	100700678	+	60	5.80E-27	100.0	60	Sb04g006120	LOC_Os02g09750	At3g10230
TC279560	GRMZM2G324297	5.80E-27	NP_001143743 (302 aa)	Putative arogenate dehydrogenase isoform 2	1	60	-	Chr:9	61356034	61356093	+	60	5.80E-27	100.0	60	Sb10g021300	LOC_Os06g35050	At5g34930
TC313810	GRMZM2G039583	5.80E-27	NP_001140673 (267 aa)	SNARE associated Golgi protein	1	60	-	Chr:7	168084179	168084238	+	60	5.80E-27	100.0	60	Sb01g034220	LOC_Os07g44020	At2g02370

TC309993	GRMZM2G001803	5.80E-27	ND	ND	1	60	+	Chr:10 *	62267147	62267206	+	60	5.80E-27	100.0	60	ND	LOC_Os08g36420	ND
TC284146	GRMZM2G363253	5.80E-27	NP_001132199 (315 aa)	RING-finger domain	1	60	-	Chr:8	115905171	115905230	+	60	5.80E-27	100.0	60	Sb04g031430	LOC_Os05g28470, LOC_Os07g28640	ND
TC284637	GRMZM2G090262	5.80E-27	NP_001152445 (531 aa)	CPL3; RNA polymerase II ctd phosphatase	1	60	+	Chr:6	143865059	143865118	+	60	5.80E-27	100.0	60	Sb04g035920	LOC_Os05g32430	At5g58003 = AtCPL4
TC315563	GRMZM2G060324	6.40E-17	ND	Signal recognition particle 54 kDa protein 3 / SRP54 (SRP-54C)	1	29	-	Chr:6*	157824655	157824683	+	29	6.40E-17	100.0	29	Sb09g025050	LOC_Os05g43390	At1g48900
TC310683	GRMZM2G026807	5.80E-27	NP_001142197 (274 aa)	Ribulose-phosphate 3-epimerase, chloroplast precursor	1	60	+	Chr:1	14625481	14625540	+	60	5.80E-27	100.0	60	ND	LOC_Os03g07300	At5g61410
TC307549	GRMZM2G315726	4.20E-17	NP_001136720 (178 aa)	ASC1-like protein 2 (Alternaria stem canker resistance-like protein 2) ; similar to At LAG1 LONGEVITY ASSURANCE HOMOLOG 3	1	24	+	Chr:5*	204582949	204582972	+	24	4.20E-17	100.0	24	Sb04g029190	LOC_Os02g49590	At1g13580=At LAG13
CD436448	GRMZM2G342807	5.80E-27	ND	Unknown function	1	60	+	Chr:9	152826503	152826562	+	60	5.80E-27	100.0	60	ND	LOC_Os03g06180	At2g21720
TC285351	No hit																	
TC312972	GRMZM2G055795, GRMZM2G037413	5.80E-27	AAR23311 (1007 aa)	Cellulose synthase catalytic subunit 11	1	60	+	Chr:3*	198371821	198371880	+	60	5.80E-27	100.0	60	Sb03g034680	LOC_Os01g54620 (Os01g0750300 = OsCesA4)	ND
TC287674	GRMZM2G419806	5.80E-27	NP_001105917 (415 aa)	Oil yellow1 (magnesium chelatase subunit I)	1	60	-	Chr:10	9224067	9224126	+	60	5.80E-27	100.0	60	Sb08g004300	LOC_Os03g36540	At4g18480
TC306331	GRMZM2G349996	5.80E-27	ND	ND	1	60	-	Chr:5	127809679	127809738	+	60	5.80E-27	100.0	60	ND	LOC_Os02g19770	ND
TC295239	GRMZM5G841619	1.40E-24	NP_001123592 (319 aa)	Tryptophan synthase alpha	1	60	+	Chr:7	10776664	10776723	+	56	1.40E-24	98.3	60	Sb02g004580	LOC_Os07g08430	At3g54640
TC299289	GRMZM2G122479	5.80E-27	NP_001105383 (662 aa)	Non-photosynthetic NADP-malic enzyme	1	60	+	Chr:6	139280914	139280973	+	60	5.80E-27	100.0	60	Sb03g003220	LOC_Os01g09320	At1g79750 = ATNADP-ME4
TC289387	GRMZM2G439596	5.80E-27	ND	ND	1	60	+	Chr:5	138806170	138806229	+	60	5.80E-27	100.0	60	ND	LOC_Os02g16450	ND
CF635716	GRMZM2G034631	5.80E-27	NP_001159047 (511 aa)	Unknown protein	1	60	+	Chr:6	76491452	76491511	+	60	5.80E-27	100.0	60	Sb10g004013	LOC_Os06g06370	At1g08030
TC302888	GRMZM2G534430	5.80E-27	ND	ND	1	60	-	Chr:5	163945288	163945347	+	60	5.80E-27	100.0	60	ND	ND	ND
TC312091	GRMZM2G139903	1.40E-24	NP_001149062 (281 aa)	Electron transport SCO1/SenC family protein	1	60	+	Chr:2	177184425	177184484	+	56	1.40E-24	98.3	60	Sb02g023290	LOC_Os09g20430	At4g39740 = AtSCO1
BM340065	No gene annotated	*2.3*	ND	ND	41	58	-	Chr:1	229174422	229174439	+	18	2.3	100.0	18	ND	ND	ND
TC311135	GRMZM2G078283	5.80E-27	ND	ND	1	60	-	Chr:4	48543739	48543798	+	60	5.80E-27	100.0	60	ND	LOC_Os08g45160	ND
TC297564	GRMZM2G131012	5.80E-27	NP_001168772 (154 aa)	Unknown protein	1	60	+	Chr:1	251079888	251079947	+	60	5.80E-27	100.0	60	Sb01g013610	LOC_Os12g42170	ND
TC288463	GRMZM2G048324	5.80E-27	NP_001105407 (569 aa)	Nucleoredoxin1, PDI-like protein	1	60	+	Chr:1	70428600	70428659	+	60	5.80E-27	100.0	60	Sb01g032890	LOC_Os03g29240, LOC_Os03g29190 = OsNrx1-2	At1g60420
TC309174	GRMZM2G419409	5.80E-27	ND	SUVH5 (SU(VAR)3-9 HOMOLOG 5); histone H3-K9 methyltransferase	1	60	-	Chr:4*	74638370	74638429	+	60	5.80E-27	100.0	60	Sb07g019830, Sb07g019850, Sb07g019860	LOC_Os08g30900, LOC_Os08g30850, LOC_Os08g30870	At2g35160 = SUVH5
TC293566	GRMZM2G093945	1.70E-17	NP_001149130 (332 aa)	Apospory-associated protein C (D-hexose-6-phosphate epimerase-like**)**	1	44	+	Chr:4*	70840457	70840500	+	44	1.70E-17	100.0	44	Sb04g005170	LOC_Os08g14330	ND

DN586214	GRMZM2G111436	5.80E-27	NP_001149685 (204 aa)	**Werner syndrome ATP-dependent helicase (bind RECQ homologs)**	1	60	+	Chr:3	200068665	200068724	+	60	5.80E-27	100.0	60	Sb03g033980	LOC_Os04g14810	At4g13870
TC293183	GRMZM2G006468	5.80E-27	NP_001105908 (238 aa)	Putative wound responsive protein	1	60	-	Chr:4	59649525	59649584	+	60	5.80E-27	100.0	60	ND	LOC_Os08g26870	At1g75380
CF040072	GRMZM2G162497	1.40E-24	NP_001145298 (394 aa)	Unknown protein	1	60	-	Chr:6	100084927	100084986	+	56	1.40E-24	98.3	60	Sb02g032880	LOC_Os09g07160	ND
TC288800	GRMZM2G099080	5.80E-27	ACG39233 (511 aa)	PX domain containing protein, similar to At SORTING NEXIN 2b	1	60	+	Chr:3	183844654	183844713	+	60	5.80E-27	100.0	60	Sb03g038540	LOC_Os01g61150	At5g07120 = SNX2b
TC304557	GRMZM2G104425	5.80E-27	ND	ND	1	60	-	Chr:5	81081828	81081887	+	60	5.80E-27	100.0	60	ND	LOC_Os02g05930	ND
BM500607	GRMZM2G007736	1.40E-24	ND	Alpha-trehalose-phosphate synthase (UDP-forming), similar to At TPS6	1	60	+	Chr:4	61410478	61410537	+	56	1.40E-24	98.3	60	Sb07g021920	LOC_Os08g34580	At1g68020 = ATTPS6
TC284770	GRMZM2G408294	4.10E-19	ND	Ribonuclease H fold domain containing protein	1	60	+	Chr:8	6944112	6944171	+	48	4.10E-19	95.0	60	Sb03g008910	LOC_Os01g01400	At5g07380
CX725290	GRMZM2G111441	5.80E-27	ND	ND	1	60	-	Chr:3	172675546	172675605	+	60	5.80E-27	100.0	60	ND	LOC_Os01g65530	ND
TC309747	AC207628.4_FG005	4.00E-13	ND	ND	16	60	+	Chr:3	202941681	202941725	+	41	4.00E-13	97.8	45	ND	LOC_Os01g51770	ND
TC304232	GRMZM2G310739	5.80E-27	NP_001152213 (481 aa)	Glucan endo-1,3-beta-glucosidase 5	1	60	+	Chr:6	161115209	161115268	+	60	5.80E-27	100.0	60	Sb09g026690	LOC_Os05g45860	At4g17180
TC313491	GRMZM2G129175	5.80E-27	ND	ND	1	60	-	Chr:7	5552356	5552415	+	60	5.80E-27	100.0	60	ND	LOC_Os07g05870+I 121	ND
TC283445	GRMZM2G143590	1.40E-24	NP_001182397 (867 aa)	**ZYP1 protein (transverse filament of the synaptonemal complex)**	1	60	-	Chr:10	121717900	121717959	+	56	1.40E-24	98.3	60	Sb01g039160	LOC_Os04g37960	At1g22260 = AtZYP1a, At1g22275 = AtZYP1b
TC296255	GRMZM2G080930	8.6	NP_001132794 (173 aa)	Putative RSZp22 splicing factor	1	20	+	Chr:5	213039048	213039064	+	17	8.6	100.0	17	Sb04g035540	LOC_Os02g54770	At4g31580
TC309875	GRMZM2G028763	5.80E-27	NP_001151176 (397 aa)	Membrane related protein-like	1	60	+	Chr:5	159928705	159928764	+	60	5.80E-27	100.0	60	Sb04g017500	LOC_Os02g26860	At3g13062
DT943270	GRMZM2G170927	5.80E-27	NP_001146732 (771 aa)	Vacuolar proton-translocating inorganic pyrophosphatase (VP1)	1	60	-	Chr:9	94180908	94180967	+	60	5.80E-27	100.0	60	Sb10g025280	LOC_Os06g43660 (Os06g0644200 = OVP1	At1g15690 = At AVP1
TC295272	GRMZM2G336761	5.80E-27	ACG42347 (386 aa)	Zinc finger, C3HC4 type family protein	1	60	-	Chr:4	234400305	234400364	+	60	5.80E-27	100.0	60	Sb07g028950	LOC_Os02g09820	At3g02340
DT943054	GRMZM2G318174, GRMZM2G072088	5.80E-27	ND	Unknown protein	1	60	-	Chr:4*	223287460	223287519	+	60	5.80E-27	100.0	60	Sb01g041830	LOC_Os03g12550, LOC_Os03g12550	At2g41960
TC312257	GRMZM2G169089	3.70E-16	ABV91586 (494 aa)	Diacylglycerol acyltransferase	1	20	-	Chr:6*	104861302	104861321	+	20	3.70E-16	100.0	20	ND	LOC_Os06g36800	ND
TC283431	GRMZM2G070639	3.40E-22	NP_001146146 (158 aa)	ND	9	60	-	Chr:4	29169276	29169327	+	52	3.40E-22	100.0	52	ND	LOC_Os08g08230	ND
TC314544	GRMZM2G329710	5.80E-27	ND	ND	1	60	+	Chr:6	72806452	72806511	+	60	5.80E-27	100.0	60	ND	LOC_Os02g28970	ND
TC280740	GRMZM2G062024	5.80E-27	NP_001152095 (599 aa)	Carbohydrate transporter/ sugar porter/ transporter	1	60	-	Chr:2	26847703	26847762	+	60	5.80E-27	100.0	60	Sb06g023125	LOC_Os04g44430	At5g64500
TC309440	GRMZM2G093157	5.80E-27	NP_001149734 (453 aa)	**Cyclin-SOLO DANCERS-like protein**	1	60	+	Chr:9	145503934	145503993	+	60	5.80E-27	100.0	60	Sb01g042340	LOC_Os03g12414	At1g14750 = SDS
CF019406	No annotated gene	9.30E-16	ND	ND	1	34	+	Chr:6*	94405451	94405484	+	34	9.30E-16	100.0	34	ND	ND	ND
DT650280	GRMZM2G067563	8.70E-23	NP_001143249 (441 aa)	Unknown protein	1	57	-	Chr:5	175272777	175272833	+	53	8.70E-23	98.3	57	Sb04g023430	LOC_Os02g35830	At3g20300

TC295182	GRMZM5G851546, GRMZM2G138566	4.10E-19	NP_001147122 (472 aa)	Calcium binding atopy-related autoantigen 1	1	60	+	Chr:3*	113760547	113760606	+	48	4.10E-19	95.0	60	Sb04g025880	LOC_Os02g39950	At4g32060
TC288590	GRMZM2G097135	5.80E-27	ACG47983 (397 aa)	BAG domain containing protein (BCL-2-ASSOCIATED ATHANOGENE 5)	1	60	-	Chr:4	21320600	21320659	+	60	5.80E-27	100.0	60	Sb05g018030	LOC_Os11g31060	At1g12060 = AT BAG 5
TC289757	AC199705.3_FG008	5.80E-27	ND	ND	1	60	+	Chr:10	129944225	129944284	+	60	5.80E-27	100.0	60	ND	LOC_Os04g43030	ND
TC308341	GRMZM2G008226	1.40E-24	ND	Transferring glycosyl groups / trehalose-phosphatase	1	60	-	Chr:1	218568679	218568738	+	56	1.40E-24	98.3	60	Sb07g020270	LOC_Os08g31980, LOC_Os05g06160, LOC_Os12g32130, LOC_Os05g03810	At1g23870 = ATTPS9
TC311848	(GRMZM2G157505	5.80E-27	NP_001152610 (101 aa)	EGG APPARATUS-1 protein	1	60	+	Chr:7	164457084	164457143	+	60	5.80E-27	100.0	60	Sb02g038820	Os07g0605400	ND
TC280797	GRMZM2G136455	5.80E-27	ND	ND	1	60	+	Chr:6	42732597	42732656	+	60	5.80E-27	100.0	60	ND	LOC_Os05g01490	ND
TC302844	GRMZM2G007791	5.80E-27	NP_001169873 (330 aa)	Protein Argonaute ; similar to AGO2	1	60	-	Chr:2	9971973	9972032	+	60	5.80E-27	100.0	60	Sb06g028510	LOC_Os04g52540 (Os04g0615700 = OsAGO2), LOC_Os04g52550 (Os04g0615800 = OsAGO3)	At1g31290 = AtAGO3
TC289753	GRMZM5G878823	5.80E-27	NP_001145579 (120 aa)	Putative RNA-binding protein RNP-D precursor	1	60	+	Chr:6	4841138	4841197	+	60	5.80E-27	100.0	60	Sb07g001610	LOC_Os08g02390	At1g01080
TC287318	GRMZM2G028369	8.70E-23	ACG40583 (312 aa)	Chorismate mutase type II	1	53	+	Chr:3	194549278	194549330	+	53	8.70E-23	100.0	53	Sb03g035460	LOC_Os01g55870	At3g29200 = AtCM1
TC307997	GRMZM5G892926	5.80E-27	ND	ND	1	60	+	Chr:7	148486563	148486622	+	60	5.80E-27	100.0	60	ND	ND	ND
DT943243	No annotated gene	2.70E-08	ND	ND	1	39	+	Chr:6	3063780	3063818	+	31	2.70E-08	94.9	39	ND	ND	ND
TC307673	No annotated gene	4.10E-19	ND	ND	1	60	+	Chr:10	124148162	124148220	+	48	4.10E-19	95.0	60	ND	ND	ND
TC301790	GRMZM2G354827	5.80E-27	NP_001168288 (175 aa)	Benzothiadiazole-induced somatic embryogenesis receptor kinase 1 (SERK1)	1	60	-	Chr:4	30203073	30203132	+	60	5.80E-27	100.0	60	ND	Os08g0174700 = OsSERK1	At1g71830 = AtSERK1
TC297030	GRMZM2G020766	5.80E-27	ND	Pseudogene of a potassium transporter	1	60	-	Chr:7	172260136	172260195	+	60	5.80E-27	100.0	60	ND	LOC_Os07g47350	ND
TC310187	Multiple hits	5.80E-27	ND	Ribulose bisphosphate carboxylase large chain precursor	1	60	+	Chr:Pt	58180	58239	+	60	5.80E-27	100.0	60	ND	ND	ND
TC301356	GRMZM2G043509	5.80E-27	NP_001146148 (572 aa)	Endonuclease/exonuclease/phosphatase family protein	1	60	+	Chr:5	34326190	34326249	+	60	5.80E-27	100.0	60	Sb01g022180	LOC_Os10g27230	At3g58560
TC285165	GRMZM2G432075	1.20E-15	ND	ND	1	32	-	Chr:7*	71984691	71984722	+	32	1.20E-15	100.0	32	ND	LOC_Os07g16224	ND
TC281589	GRMZM2G159034	9.00E-18	NP_001168395 (203 aa)	Putative ankyrin-kinase	1	46	-	Chr:7	167841314	167841359	+	46	9.00E-18	100.0	46	Sb03g034570	LOC_Os07g43900	At1g14000
TC306070	GRMZM2G014419	1.40E-02	NP_001140496 (239 aa)	Late embryogenesis abundant protein 2 (LEA2)	1	60	-	Chr:1	37664348	37664407	+	56	1.40E-24	98.3	60	Sb01g040310 H158	LOC_Os03g15630	At2g01080
TC279657	GRMZM5G823563	1.40E-02	NP_001170355 (208 aa)	Unknown protein	1	56	-	Chr:2	19452782	19452837	+	56	1.40E-24	100.0	56	ND	ND	ND
TC313076	GRMZM2G006341	2.30E-17	ND	ND	1	22	+	Chr:9*	136515191	136515212	+	22	2.30E-17	100.0	22	ND	LOC_Os03g18980	ND
TC306072	GRMZM2G179215	5.80E-27	ND	ND	1	60	-	Chr:8	155003174	155003233	+	60	5.80E-27	100.0	60	ND	LOC_Os01g51700	ND
CO441573	GRMZM2G083975	1.30E-21	ND	RNA recognition motif family protein	1	58	+	Chr:6	124863479	124863537	+	51	1.30E-21	96.6	59	Sb09g004685	Os03g0285900	At3g53500

TC280985	GRMZM2G356938	0.0087	NP_001148812 (328 aa)	Voltage-gated potassium channel beta subunit	10	31	+	Chr:5	215816393	215816414	+	22	0.0087	100.0	22	Sb04g037250	LOC_Os02g57240	ND
CA827264	GRMZM5G882708	5.80E-27	ACG34546 (398 aa)	Vacuolar membrane protein	1	60	+	Chr:2	5831398	5831457	+	60	5.80E-27	100.0	60	Sb06g030570	LOC_Os04g55260	At4g32140
TC285655	GRMZM2G173428	5.80E-27	ND	CID11	1	60	-	Chr:6	4434038	4434097	+	60	5.80E-27	100.0	60	ND	LOC_Os08g02330	ND
TC284552	No annotated gene	1.10E-07	ND	ND	23	60	+	Chr:6	127436141	127436178	+	30	1.10E-07	94.7	38	ND	ND	ND
DT647408	GRMZM2G155806	4.10E-19	ACG37722 (328 aa)	Pumilio/Puf RNA-binding domain-containing protein-like	1	60	-	Chr:7	101123486	101123545	+	48	4.10E-19	95.0	60	Sb10g021290	LOC_Os09g20560	At4g10610
TC315034	No annotated gene	4.10E-19	ND	ND	1	60	-	Chr:9	129403528	129403587	+	48	4.10E-19	95.0	60	ND	ND	ND
TC313063	GRMZM2G032047	5.80E-27	ACG37172 (170 aa)	Cp protein ; Protein of unknown function, DUF538	1	60	-	Chr:1	46125118	46125177	+	60	5.80E-27	100.0	60	Sb01g038180	Os03g0297000	At5g37070
TC281079	GRMZM2G027173	5.80E-27	NP_001167675 (266 aa)	Phagocytosis and cell motility ELMO domain-containing protein 2	1	60	-	Chr:10	134431414	134431473	+	60	5.80E-27	100.0	60	Sb06g024230	LOC_Os04g46079	At2g44770
TC283790	GRMZM2G342685	1.40E-24	NP_001151906 (243 aa)	Caleosin (RESPONSIVE TO DESSICATION 20), Ca2+-binding EF-hand protein	1	60	+	Chr:1	27868287	27868346	+	56	1.40E-24	98.3	60	Sb01g042490	LOC_Os03g12230	At2g33380 =RD20
CN844996	GRMZM2G149392	5.80E-27	NP_001146434 (231 aa)	Unknown protein	1	60	-	Chr:6	142947627	142947686	+	60	5.80E-27	100.0	60	ND	AAV31331	At4g25330
TC303407	GRMZM2G068331	3.40E-22	NP_001159230 (591 aa)	**PHD-finger family protein, similar to male meiocyte death1 (MMD1) /DUET**	1	60	-	Chr:4	10230334	10230393	+	52	3.40E-22	96.7	60	Sb03g041550	LOC_Os11g12650	At1g33420 = MMD1/DUET
TC283852	GRMZM2G459702	5.80E-27	NP_001130340 (701 aa)	Potential U2 snRNA pseudouridine synthase-like	1	60	-	Chr:7	167854247	167854306	+	60	5.80E-27	100.0	60	Sb02g040320	LOC_Os01g56620	At3g04820
DR813132	GRMZM2G169173	3.40E-22	NP_001151251 (408 aa)	F-box domain containing protein	1	60	-	Chr:1	47369737	47369796	+	52	3.40E-22	96.7	60	Sb01g037880	Os12g0128000, LOC_Os10g03600	At3g07870
TC306103	No annotated gene	5.80E-27	ND	ND	1	60	+	Chr:4	175273606	175273665	+	60	5.80E-27	100.0	60	ND	ND	ND
TC289774	GRMZM2G106604	5.80E-27	NP_001143072 (457 aa)	Unknown protein	1	60	-	Chr:2	226376413	226376472	+	60	5.80E-27	100.0	60	Sb08g014930	Os12g0488700	At3g54740
DN559761	GRMZM2G135073	5.80E-27	NP_001151604 (690 aa)	STE20/SPS1-related proline-alanine-rich protein kinase	1	60	-	Chr:4	174767281	174767340	+	60	5.80E-27	100.0	60	Sb10g014850	LOC_Os02g54900	At4g24100
DR795221	GRMZM2G019596	5.80E-27	NP_001170517 (362 aa)	MSP (Major sperm protein) domain, VAMP/SYNAPTOBREVIN-ASSOCIATED PROTEIN 27-2	1	60	-	Chr:8	115230316	115230375	+	60	5.80E-27	100.0	60	Sb09g018590	Os01g0936800, LOC_Os05g30950	At1g08820 =VAP27-2
TC314264	GRMZM2G423333	6.30E-20	ND	ND	12	60	-	Chr:5	170186492	170186540	+	49	6.30E-20	100.0	49	ND	LOC_Os07g28850	ND
TC291467	GRMZM2G174574	5.80E-27	NP_001146510 (325 aa)	4-Coumarate-CoA ligase-like	1	60	+	Chr:1	245028760	245028819	+	60	5.80E-27	100.0	60	Sb01g016420	Os12g0488700	At3g54740
BM259506	GRMZM2G063316	3.40E-22	NP_001151430 (491 aa)	SET domain containing protein	1	60	-	Chr:4	166972768	166972827	+	52	3.40E-22	96.7	60	Sb09g030160	LOC_Os02g50100	At1g24610
AW231811	GRMZM2G033236	8.5	ND	ND	25	41	+	Chr:4	198780909	198780925	+	17	8.5	100.0	17	ND	ND	ND
AI944295	No annotated gene	1.40E-24	ND	ND	1	60	-	Chr:5	34019355	34019414	+	56	1.40E-24	98.3	60	ND	ND	ND
CF633046	GRMZM2G055501	9.70E-18	ND	ND	1	18	+	Chr:6*	19212445	19212462	+	18	9.70E-18	100.0	18	ND	LOC_Os09g39790	ND

TC289727	GRMZM2G083950	5.80E-27	AAC25599 (668 aa)	Chloroplast RNA processing1, Pentatricopeptide repeat-containing protein	1	60	-	Chr:7	155833857	155833916	+	60	5.80E-27	100.0	60	Sb02g035800	LOC_Os07g36390	At5g42310
TC283691	GRMZM2G588241	5.80E-27	ND	ND	1	60	-	Chr:1	229518536	229518595	+	60	5.80E-27	100.0	60	ND	ND	ND
TC297993	GRMZM2G105387	3.40E-22	NP_001148873 (227 aa)	MADS-box transcription factor 26, similar to AGAMOUS-LIKE 12	1	60	+	Chr:4	38758329	38758388	+	52	3.40E-22	96.7	60	Sb07g001250	LOC_Os08g02070	At1g71692 = AGL12
TC307363	GRMZM2G139931	5.80E-27	NP_001132496 (163 aa)	Probable potassium transporter 14	1	60	-	Chr:7	152178704	152178763	+	60	5.80E-27	100.0	60	Sb02g034330	LOC_Os07g32530 (Os07g0509200 = OsHAK14)	At5g09400 = AtHAK7
TC286486	GRMZM2G071630	5.80E-27	NP_001105385 (337 aa)	Cytosolic glyceroldehyde-3-phosphate dehydrogenase GAPC3	1	60	+	Chr:4	133107048	133107107	+	60	5.80E-27	100.0	60	Sb04g025120	LOC_Os02g38920	At1g13440
TC302041	AC235547.1_FG005	5.80E-27	BAD95671 (790 aa)	Wound and phytochrome signaling involved receptor like kinase	1	60	-	Chr:1	282405907	282405966	+	60	5.80E-27	100.0	60	Sb01g006710	LOC_Os03g56250	At4g39270
TC284424	GRMZM2G134502	2.50E-17	ND	ND	1	21	-	Chr:2*	185379524	185379544	+	21	2.50E-17	100.0	21	ND	LOC_Os09g27990	ND
TC310988	GRMZM2G138907	3.40E-22	ACG36947 (371 aa)	GDP-mannose 3,5-epimerase 2	1	52	+	Chr:4	9757828	9757879	+	52	3.40E-22	100.0	52	Sb01g021890	LOC_Os11g37890	At5g28840
TC279890	GRMZM2G005036	5.80E-27	NP_001146680 (499 aa)	Mitochondrial processing peptidase alpha subunit	1	60	-	Chr:6	160041039	160041098	+	60	5.80E-27	100.0	60	Sb03g003160	LOC_Os05g44916	At3g16480
TC295938	GRMZM2G060349	5.80E-27	NP_001168465 (689 aa)	**DNA mismatch repair protein MutS2-like**	1	60	-	Chr:5	23337156	23337215	+	60	5.80E-27	100.0	60	Sb01g017480	LOC_Os10g36530	At5g54090
TC281453	GRMZM2G143211	5.80E-27	ACR36024 (417 aa)	WD40 domain , autophagy gene ATG18d	1	60	+	Chr:8	16745735	16745794	+	60	5.80E-27	100.0	60	Sb03g004650	LOC_Os01g07400	At3g56440 =AtATG18d
TC286746	GRMZM2G115504	5.80E-27	NP_001150434 (366 aa)	Hydrolase, alpha/beta fold protein-like	1	60	+	Chr:5	168889398	168889457	+	60	5.80E-27	100.0	60	Sb04g021660	LOC_Os02g32970	At2g36290
TC284316	GRMZM2G019596	5.80E-27	NP_001170517 (362 aa)	Putative VAMP/synaptobrevin-associated protein 27-2 (VAP27)	1	60	-	Chr:8	115234650	115234709	+	60	5.80E-27	100.0	60	Sb03g045240	Os01g0936800, LOC_Os05g30950	At1g08820
TC291853	GRMZM2G003916	1.40E-24	ND	ND	3	58	-	Chr:10	124756784	124756839	+	56	1.40E-24	100.0	56	ND	ND	ND
TC310688	GRMZM2G178960	4.10E-19	NP_001148585 (274 aa)	Ribulose-phosphate 3-epimerase	1	56	-	Chr:9	151316259	151316314	+	48	4.10E-19	96.4	56	ND	LOC_Os03g07300	At5g61410
TC314530	GRMZM2G133006	5.80E-27	NP_001140720 (473 aa)	MATE family efflux protein	1	60	-	Chr:6	139595236	139595295	+	60	5.80E-27	100.0	60	Sb09g017210	LOC_Os10g20470	At5g52450
CD573220	GRMZM5G818213	5.80E-27	NP_001141804 (333 aa)	Lipase class 3 family protein	1	60	+	Chr:1	173679423	173679482	+	60	5.80E-27	100.0	60	Sb08g021485	Os05g0390000, LOC_Os12g42010, LOC_Os12g41970, LOC_Os12g41980	At1g06800
TC296845	GRMZM2G067350	5.80E-27	NP_001130479 (250 aa)	4/1 protein	1	60	-	Chr:5	80196102	80196161	+	60	5.80E-27	100.0	60	Sb04g003530	LOC_Os02g05520	At4g26020
TC308047	GRMZM5G854901	5.80E-27	ND	tRNA/rRNA methyltransferase (SpoU) family protein	1	60	-	Chr:1	188014703	188014762	+	60	5.80E-27	100.0	60	Sb07g027830	LOC_Os08g39600, LOC_Os08g39610	At5g26880
TC314676	GRMZM2G119523	5.80E-27	NP_001140861 (424 aa)	Unknown protein	1	60	-	Chr:3	90410520	90410579	+	60	5.80E-27	100.0	60	Sb01g006430	LOC_Os12g43580, LOC_Os05g40920	ND
TC304579	GRMZM2G164470	5.80E-27	ND	Early-responsive to dehydration protein	1	60	+	Chr:5	9376926	9376985	+	60	5.80E-27	100.0	60	Sb01g009540	LOC_Os03g51620	At1g58520
TC289341	GRMZM2G116243	5.80E-27	NP_001130613 (554 aa)	Calcineurin B subunit-related	1	60	+	Chr:5	60002671	60002730	+	60	5.80E-27	100.0	60	ND	LOC_Os06g49790	At2g45670
TC310354	GRMZM2G028640	5.80E-27	NP_001150376 (145 aa)	Enzyme of the cupin superfamily	1	60	+	Chr:2	45958144	45958203	+	60	5.80E-27	100.0	60	Sb04g026280	LOC_Os04g36760	At4g10300

TC286055	No annotated gene	2.20E-23	ND	ND	7	60	+	Chr:5	5198890	5198943	+	54	0.0	100.0	54	ND	ND	ND
TC282058	GRMZM2G108655	1.10E-20	NP_00113713 3 (294 aa)	Similar to COP1-interacting protein 7 (CIP7)-like	1	50	+	Chr:9	131731461	131731510	+	50	1.10E-20	100.0	50	Sb01g036410	LOC_Os03g21270	At1g72410
TC283684	GRMZM5G863229	9.70E-17	NP_00113158 6 (400 aa)	EamA-like transporter family ; similar to At Golgi Nucleotide Sugar Transporter 1	1	20	-	Chr:4*	39783997	39784016	+	20	9.70E-17	100.0	20	Sb07g000765	LOC_Os08g01610	At2g13650
CD995221	GRMZM2G349247	5.80E-27	ND	ND	1	60	-	Chr:3	88010089	88010148	+	60	5.80E-27	100.0	60	ND	ND	ND
TC284035	GRMZM5G890815	9.10E-26	ND	ND	3	60	-	Chr:7*	173070503	173070560	+	58	9.10E-26	100.0	58	ND	LOC_Os12g18530	ND
TC295868	No hit		ND	ND												ND	ND	ND
BG841754	GRMZM2G026050	5.80E-27	NP_00113070 9 (124 aa)	PPPDE putative peptidase domain	1	60	-	Chr:2	20530949	20531008	+	60	5.80E-27	100.0	60	Sb06g024410	LOC_Os04g46290	At5g47310
TC279480	GRMZM2G041328	7.90E-16	NP_00113083 0 (538 aa)	Histone-arginine methyltransferase CARM1	1	51	+	Chr:7	172584231	172584280	+	43	7.90E-16	96.1	51	Sb02g042540	LOC_Os07g47500	At3g06930 = At PRMT4b
DT943053	GRMZM2G103119, GRMZM2G318174, GRMZM2G072088	5.80E-27	ND	Unknown protein	1	60	+	Chr:9*	144446481	144446540	+	60	5.80E-27	100.0	60	Sb01g041830	LOC_Os03g12550, LOC_Os03g12550, LOC_Os03g12550	At3g58050
TC287319	GRMZM2G124365	5.80E-27	NP_00114598 3 (312 aa)	Chorismate mutase type II	1	60	+	Chr:8	173107428	173107487	+	60	5.80E-27	100.0	60	Sb03g035460	LOC_Os01g55870	At1g69370
TC293287	GRMZM2G063961	5.80E-27	ACG46236 (359 aa)	Serine/threonine-protein kinase SAPK4	1	60	+	Chr:6	150465569	150465628	+	60	5.80E-27	100.0	60	Sb09g021410	Os01g0869900, LOC_Os05g35770, LOC_Os05g35760	At1g10940
TC302095	GRMZM2G000397	1.40E-24	NP_00115057 1 (172 aa)	EF hand (Calcium binding motif) family protein	1	60	+	Chr:2	12130114	12130173	+	56	1.40E-24	98.3	60	Sb06g027480	LOC_Os04g51240	At1g64850
TC292387	GRMZM2G003762	3.40E-22	NP_00118368 9 (238 aa)	Glutamine amidotransferases class-II (GATase)	1	60	-	Chr:10 *	4673930	4673989	+	52	3.40E-22	96.7	60	Sb05g002860	LOC_Os11g05050, LOC_Os12g05050	At5g19140
TC307255	GRMZM2G438524	5.80E-02	NP_00113029 1 (716 aa)	67kD chloroplastic RNA-binding protein	1	60	+	Chr:1	299407277	299407336	+	60	5.80E-27	100.0	60	Sb01g042790	LOC_Os03g63910	At4g16390
TC290945	GRMZM2G459702	5.80E-27	NP_00113034 0 (701 aa)	Potential U2 snRNA pseudouridine synthase-like	1	60	-	Chr:7	167863928	167863987	+	60	5.80E-27	100.0	60	Sb02g040320	LOC_Os01g56620	At3g04820
TC295259	GRMZM2G081310	1.60E-14	Q41789_MAIZ E (451 aa)	Calcium-dependent protein kinase	16	60	-	Chr:4	156995794	156995838	+	41	1.60E-14	97.8	45	Sb04g031570	LOC_Os02g46090	At4g35310= CDPK5
TC311526	GRMZM2G456626	5.80E-27	Q84Y01_MAIZ E (342 aa)	Inositol-tetrakisphosphate 1-kinase 1	1	60	-	Chr:1	101831017	101831076	+	60	5.80E-27	100.0	60	Sb01g028090	LOC_Os10g42550	At5g16760
TC289461	GRMZM2G140994	5.80E-27	ACG37501 (344 aa)	Rhomboid domain containing 1	1	60	-	Chr:8	1353083	1353142	+	60	5.80E-27	100.0	60	Sb03g011560	LOC_Os01g18100	At3g58460
DR906542	GRMZM2G039650	5.80E-27	NP_00116863 4 (623 aa)	Unknown protein, putative phosphatase domain	1	60	+	Chr:3	177675491	177675550	+	60	5.80E-27	100.0	60	Sb03g040355	LOC_Os01g63820	ND
TC293448	GRMZM2G138060	5.80E-27	AAB97167 (789 aa)	Sugary1 isoamylase	1	60	+	Chr:4	41377903	41377962	+	60	5.80E-27	100.0	60	Sb07g027200	LOC_Os08g40930	At2g39930 = AtISA1
TC305399	GRMZM2G050329	5.80E-27	ND	SPX (SYG1/Pho81/XPR1) domain-containing protein	1	60	-	Chr:9	24461904	24461963	+	60	5.80E-27	100.0	60	Sb06g025950	LOC_Os06g03860	At1g63010
DT645987	GRMZM2G177461	5.80E-27	NP_00117075 8 (509 aa)	Putative Myo-inositol-1-phosphate synthase (MI-1-P synthase)	1	60	+	Chr:1	137822547	137822606	+	60	5.80E-27	100.0	60	Sb12s002210	LOC_Os10g22450	At2g22240 = MIPS2

TC306328	GRMZM2G161787	5.80E-27	ND	Ndr family protein	1	60	+	Chr:8	20309791	20309850	+	60	5.80E-27	100.0	60	Sb03g003080	LOC_Os01g09670	At5g56750
TC307982	GRMZM2G043992	1.40E-24	NP_001146378 (389 aa)	Unknown protein	5	60	-	Chr:2	2819667	2819722	+	56	1.40E-24	100.0	56	ND	LOC_Os03g51260	ND
TC294630	GRMZM2G114113	5.80E-27	ND	ND	1	60	+	Chr:5	199769985	199770044	+	60	5.80E-27	100.0	60	ND	LOC_Os02g47130, LOC_Os07g07210	ND
TC279806	GRMZM5G802801	5.80E-27	ND	HSP70 (heat shock protein 70); ATP binding	1	60	-	Chr:8	116617900	116617959	+	60	5.80E-27	100.0	60	Sb09g022580	Os01g0840100	At3g12580
TC295047	GRMZM2G153899	5.80E-27	NP_001146071 (191 aa)	Uncharacterized conserved protein (DUF2343)	1	60	+	Chr:4	237398813	237398872	+	60	5.80E-27	100.0	60	Sb04g003740	LOC_Os02g05870	At1g53760
TC310367	GRMZM5G828630	2.30E-26	ND	ND	1	59	+	Chr:1	141736660	141736718	+	59	2.30E-26	100.0	59	ND	LOC_Os10g25130	ND
CD447985	GRMZM2G109383	2.30E-16	NP_001105703 (583 aa)	Cytoplasmic phosphoglucomutase 1	1	44	-	Chr:5	10858297	10858340	+	44	2.30E-16	100.0	44	Sb01g010280	LOC_Os03g50480	At1g23190
TC280195	GRMZM2G134708	5.80E-27	NP_001168352 (433 aa)	Pyridine nucleotide-disulphide oxidoreductase	1	60	-	Chr:4	53078629	53078688	+	60	5.80E-27	100.0	60	Sb07g024320	LOC_Os08g44340	At3g52880
TC295884	GRMZM2G148872	5.80E-27	NP_001141979 (398 aa)	SOUL heme-binding protein	1	60	+	Chr:9	86669948	86670007	+	60	5.80E-27	100.0	60	Sb10g023280	LOC_Os06g39690	At5g20140
TC289354	GRMZM2G052474	5.80E-27	NP_001146091 (268 aa)	Similar to NC domain-containing protein-related	1	60	+	Chr:7	141903686	141903745	+	60	5.80E-27	100.0	60	Sb02g030510	LOC_Os09g35810	At1g01225
TC314450	GRMZM2G068331	1.40E-24	NP_001159230 (591 aa)	PHD-finger family protein, similar to male meiocyte death1 (MMD1) /DUET	1	60	-	Chr:4	10228893	10228952	+	56	1.40E-24	98.3	60	Sb03g041550	LOC_Os11g12650	At1g33420 = MMD1/DUET
TC306026	GRMZM2G020721	5.80E-27	NP_001170173 (391 aa)	O-acetyltransferase family protein	1	60	+	Chr:9	134303131	134303190	+	60	5.80E-27	100.0	60	Sb01g037160	LOC_Os03g19970	At2g34410
TC284639	GRMZM2G006341	7.10E-17	ND	ND	1	31	+	Chr:9*	136512242	136512272	+	31	7.10E-17	100.0	31	ND	LOC_Os03g18980	ND
TC283905	GRMZM2G008528	5.80E-27	NP_001141449 (409 aa)	F-box domain containing protein	1	60	+	Chr:7	156510644	156510703	+	60	5.80E-27	100.0	60	Sb02g036090	LOC_Os07g36830	At3g16210
CF629011	GRMZM2G315726	5.80E-27	ACG46204 (307 aa)	Alternaria stem canker resistance (ASC)-like protein 2	1	60	+	Chr:5	204583540	204583599	+	60	5.80E-27	100.0	60	Sb04g029190	LOC_Os02g49590	At1g13580 =At LAG3
TC284496	GRMZM2G145061	6.70E-17	NP_001159002 (307 aa)	Prolyl 4-hydroxylase alpha-2 subunit	1	28	+	Chr:5*	216555446	216555473	+	28	6.70E-17	100.0	28	Sb04g038020	LOC_Os02g58070	At1g20270
TC309689	GRMZM2G102255	5.80E-27	ND	ABI2 (ABA INSENSITIVE 2); protein serine/threonine phosphatase	1	60	+	Chr:6	167405481	167405540	+	60	5.80E-27	100.0	60	Sb09g030600	LOC_Os05g51510	At5g57050 = AtABI2
TC295891	GRMZM2G459702	5.80E-27	NP_001130340 (701 aa)	Potential U2 snRNA pseudouridine synthase-like	1	60	-	Chr:7	167855783	167855842	+	60	5.80E-27	100.0	60	Sb02g040320	LOC_Os01g56620	At3g04820
TC290304	GRMZM2G330453	5.80E-27	NP_001143119 (499 aa)	Plasma-membrane choline transporter	1	60	+	Chr:1	259824162	259824221	+	60	5.80E-27	100.0	60	Sb01g013160	LOC_Os03g44840	At3g03700
CO526721	AC182482.3_FG003	5.80E-27	ND	ND	1	60	+	Chr:3	23011581	23011640	+	60	5.80E-27	100.0	60	ND	LOC_Os01g03914	ND
TC280500	GRMZM2G109383	1.70E-17	NP_001105703 (583 aa)	Cytoplasmic phosphoglucomutase 1	1	35	-	Chr:5*	10857462	10857496	+	35	1.70E-17	100.0	35	Sb01g010280	LOC_Os03g50480	At1g23190
TC294269	GRMZM2G102238	1.40E-24	NP_001146954 (156 aa)	Acid phosphatase/vanadium-dependent haloperoxidase related	1	60	-	Chr:2	36321958	36322017	+	56	1.40E-24	98.3	60	Sb06g020710	LOC_Os04g40980	At1g67600
TC285412	GRMZM5G806358	3.10E-16	ND	ND	1	34	-	Chr:5	69513850	69513883	+	34	3.10E-16	100.0	34	ND	LOC_Os04g25550, LOC_Os08g31240	ND
TC311214	GRMZM2G451882	3.60E-25	NP_001150045 (616 aa)	Calmodulin binding protein	1	57	-	Chr:5	89449141	89449197	+	57	3.60E-25	100.0	57	Sb04g005240	LOC_Os02g08120	At5g57580

TC282818	GRMZM2G013639	5.80E-27	NP_001141020 (368 aa)	Domon-like ligand-binding domain protein	1	60	-	Chr:1	49814889	49814948	+	60	5.80E-27	100.0	60	Sb01g037350	LOC_Os03g19580	At3g62370
TC290471	GRMZM2G109496	2.20E-23	NP_001140758 (365 aa)	Probable protein phosphatase 2C	1	60	+	Chr:10	128328389	128328450	+	54	2.20E-23	96.8	62	Sb06g021650	LOC_Os04g42260	At2g25070 = AtPPC4;2
CD995946	GRMZM2G128617	1.40E-24	ND	ND	1	60	+	Chr:1	275641233	275641292	+	56	1.40E-24	98.3	60	ND	LOC_Os03g53400	ND
CB280793	GRMZM2G029385	5.80E-27	NP_001148450 (132 aa)	Mitochondrial import inner membrane translocase subunit TIM14	1	60	+	Chr:1	283206946	283207005	+	60	5.80E-27	100.0	60	Sb01g006420	LOC_Os03g56540	At5g03030
TC292342	GRMZM2G104876	1.40E-24	NP_001150247 (199 aa)	Glycine-rich protein A3	1	56	-	Chr:6	30254670	30254725	+	56	1.40E-24	100.0	56	Sb09g001970	LOC_Os05g02780, LOC_Os05g02770	At4g19200
TC297828	GRMZM2G154146, GRMZM2G357349, AC194974.3_FG005, GRMZM2G071206	5.80E-27	ND	ND	1	60	+	Chr:9*	26242616	26242675	+	60	5.80E-27	100.0	60	ND	LOC_Os04g34620, LOC_Os04g34620, LOC_Os04g34620	ND
TC283544	GRMZM2G000665	2.00E-16	ND	ND	16	60	-	Chr:2	193165648	193165692	+	45	2.00E-16	100.0	45	ND	LOC_Os09g33830	ND
TC311757	GRMZM2G054905	1.40E-24	ND	ND	1	60	-	Chr:3	191125053	191125112	+	56	1.40E-24	98.3	60	ND	LOC_Os01g57073, LOC_Os01g57082	
DT643307	GRMZM2G018450	5.80E-27	ND	ND	1	60	-	Chr:4	233634967	233635026	+	60	5.80E-27	100.0	60	ND	ND	ND
TC313835	GRMZM2G168261	1.40E-24	NP_001146439 (319 aa)	Unknown protein	1	60	-	Chr:7	134225442	134225501	+	56	1.40E-24	98.3	60	Sb07g027140	LOC_Os09g32095, LOC_Os11g14030	At5g35320
TC284163	GRMZM2G347717	5.80E-27	NP_001141656 (312 aa)	UDP-glucuronic acid decarboxylase	1	60	+	Chr:9	138883712	138883771	+	60	5.80E-27	100.0	60	Sb01g039050	LOC_Os03g17230	At3g53520 = UXS1
TC295705	GRMZM2G157269	1.40E-24	ND	ND	1	60	-	Chr:2	60272436	60272495	+	56	1.40E-24	98.3	60	ND	LOC_Os04g33190	ND
TC313084	(GRMZM2G563190	5.80E-27	NP_001142169 (578 aa)	Putative NADPH dehydrogenase	1	60	-	Chr:3	190890866	190890925	+	60	5.80E-27	100.0	60	Sb03g036480	LOC_Os05g26660	At4g05020
TC295697	GRMZM2G044495	1.10E-20	NP_001150816 (479 aa)	Rac GTPase activating protein 2	11	60	+	Chr:5	196177489	196177538	+	50	1.10E-20	100.0	50	Sb04g031870	LOC_Os02g45600	At2g46710
DT946613	GRMZM2G310739	5.80E-27	NP_001152213 (481 aa)	Glucan endo-1,3-beta-glucosidase 5	1	60	+	Chr:6	161115897	161115956	+	60	5.80E-27	100.0	60	Sb09g026690	LOC_Os05g45860	ND
TC296831	GRMZM2G136710	5.80E-27	NP_001140262 (414 aa)	Unknown protein	1	60	-	Chr:3	191329621	191329680	+	60	5.80E-27	100.0	60	Sb03g036300	LOC_Os01g57020	At1g05577
TC312974	GRMZM2G348578	5.80E-27	NP_001150030 (294 aa)	Prolyl 4-hydroxylase	1	60	+	Chr:1	279414851	279414910	+	60	5.80E-27	100.0	60	Sb09g023860	LOC_Os03g55380	At4g17180
TC312299	AC231745.1_FG004	5.80E-27	ND	ND	1	60	+	Chr:9	18239352	18239411	+	60	5.80E-27	100.0	60	ND	ND	ND
TC301395	GRMZM2G050890	5.80E-27	NP_001148484 (193 aa)	Prenylated rab acceptor family protein	1	60	+	Chr:3	5671537	5671596	+	60	5.80E-27	100.0	60	Sb03g002890	LOC_Os01g10010	At3g13720 = AtPRA8
TC293138	GRMZM2G077823	5.80E-27	NP_001140562 (251 aa)	Putative splicing factor, arginine/serine-rich 2	1	60	+	Chr:2	212353228	212353287	+	60	5.80E-27	100.0	60	Sb01g033710	LOC_Os07g43050	At5g64200 = ATSC35
BG319836	GRMZM2G164696	1.40E-24	NP_001141544 (300 aa)	Tubulin beta-1 chain	1	60	+	Chr:1	2040052	2040111	+	56	1.40E-24	98.3	60	Sb01g050310	LOC_Os03g01530	At5g62690 = AtTUB2, AT5g62700= At TUB3
TC300898	GRMZM2G006745	5.80E-27	NP_001105876 (317 aa)	DEHYDRATION-RESPONSIVE ELEMENT BINDING PROTEIN 2 ; ERF/AP2 domain containing transcription factor	1	60	+	Chr:8	95022764	95022823	+	60	5.80E-27	100.0	60	Sb03g004980	LOC_Os05g27930	At5g05410 = AtDREB2A
BG837957	GRMZM2G146750	3.40E-15	NP_001142428 (90 aa)	**Similar to CENP-E like kinetochore protein (NP_001146907 ; 614 aa)**	4	60	+	Chr:6	123686879	123686935	+	42	3.40E-15	93.1	58	Sb09g005020	LOC_Os05g07680	At5g05180

TC292121	GRMZM2G046055	5.80E-27	ND	ND	1	60	-	Chr:5	9951711	9951770	+	60	5.80E-27	100.0	60	ND	LOC_Os03g51200	ND
TC286409	GRMZM2G180625	7.50E-08	NP_001105700 (337 aa)	Glyceraldehyde-3-phosphate dehydrogenase, cytosolic 2	1	41	-	Chr:6	6898865	6898906	+	30	7.50E-08	92.9	42	Sb04g025120	LOC_Os08g03290	At3g04120 = GAPC
TC313657	No annotated gene	8.70E-23	ND	ND	4	60	-	Chr:8	161760671	161760727	+	53	8.70E-23	98.3	57	ND	ND	ND
TC315043	GRMZM2G315199	3.40E-22	NP_001170162 (122 aa)	Unknown protein	1	56	-	Chr:6	156122783	156122838	+	52	3.40E-22	98.2	56	ND	ND	ND
BG319898	GRMZM2G003682	5.80E-27	NP_001151109 (467 aa)	Nematode-resistance protein	1	60	+	Chr:3	178100346	178100405	+	60	5.80E-27	100.0	60	Sb03g040300	LOC_Os01g63690	At3g55840
TC304530	GRMZM5G806108	5.80E-27	ND	Protein kinase family protein	1	60	-	Chr:2	51279874	51279933	+	60	5.80E-27	100.0	60	Sb06g016830	LOC_Os04g35114	At3g19300
TC280737	GRMZM2G062024	5.80E-27	NP_001152095 (599 aa)	Carbohydrate transporter/ sugar porter/ transporter	1	60	-	Chr:2	26830568	26830627	+	60	5.80E-27	100.0	60	Sb06g023125	LOC_Os04g44430	At5g64500
TC282918	GRMZM2G133048	5.80E-27	ND	Phosphatidate cytidylyltransferase family protein	1	60	+	Chr:5	78918619	78918678	+	60	5.80E-27	100.0	60	Sb04g003380	LOC_Os02g05320, LOC_Os02g04945, LOC_Os02g05400	At3g45040
CK787298	AC207814.3_FG002	3.40E-22	ND	ND	1	60	+	Chr:3	58537356	58537415	+	52	3.40E-22	96.7	60	ND	LOC_Os12g06550, LOC_Os06g06610	ND
TC310318	GRMZM2G306679	5.80E-27	NP_001130454 (159 aa)	17.4 kDa class I heat shock protein 3	1	60	+	Chr:1	38847922	38847981	+	60	5.80E-27	100.0	60	Sb01g039990	Os03g0266300	At5g59720
TC279580	GRMZM2G399901	5.80E-27	NP_001146070 (365 aa)	E3 ubiquitin-protein ligase makorin RING finger protein	1	60	-	Chr:5	203403819	203403878	+	60	5.80E-27	100.0	60	Sb04g029760	Os06g0318700 = OsC3H41	At3g63550 = C3H69
TC308668	GRMZM2G099481	7.40E-18	NP_001146282 (969 aa)	Lipin family protein	1	17	-	Chr:6*	152082910	152082926	+	17	7.40E-18	100.0	17	Sb09g022740	LOC_Os05g38710	At3g09560
TC304331	GRMZM2G457370	5.80E-27	NP_001145806 (747 aa)	Putative leaf development protein Argonaute , similar to At AGO1	1	60	+	Chr:1	250075675	250075734	+	60	5.80E-27	100.0	60	Sb02g032980	LOC_Os07g28850 (Os07g0471300 = OsAGO18)	At1g48410 = AGO1
TC296050	GRMZM2G093766	5.80E-27	ND	**myosin heavy chain-related**	1	60	-	Chr:7	5072768	5072827	+	60	5.80E-27	100.0	60	Sb02g002890	LOC_Os07g05390	At2g34730
TC302598	GRMZM2G172410	5.80E-27	ND	Phosphotransferase-related protein	1	60	+	Chr:1	12985341	12985400	+	60	5.80E-27	100.0	60	Sb01g046270	LOC_Os03g06720	At5g02940
TC284042	GRMZM2G067373	5.80E-27	ND	ND	1	60	-	Chr:6	112349476	112349535	+	60	5.80E-27	100.0	60	ND	LOC_Os06g12030	ND
TC293263	GRMZM2G105398	5.80E-27	ND	ND	1	60	+	Chr:6	102830052	102830111	+	60	5.80E-27	100.0	60	ND	LOC_Os06g39760	ND
TC287858	GRMZM5G805289	5.80E-27	ND	ND	1	60	+	Chr:6	160623282	160623341	+	60	5.80E-27	100.0	60	ND	ND	ND
TC283769	GRMZM2G442489	5.80E-27	NP_001141678 (396 aa)	F-box domain containing protein	1	60	-	Chr:9	98920427	98920486	+	60	5.80E-27	100.0	60	Sb10g024600	LOC_Os02g33840	At3g49980
TC303615	GRMZM2G090152	5.80E-27	NP_001169393 (403 aa)	Unknown protein	1	60	+	Chr:1	272830071	272830130	+	60	5.80E-27	100.0	60	Sb01g009210	ND	At5g11730
TC314126	GRMZM2G043992	5.80E-27	NP_001146378 (389 aa)	Unknown protein	1	60	-	Chr:2	2822438	2822497	+	60	5.80E-27	100.0	60	ND	LOC_Os03g51260	ND
CF626131	GRMZM2G454838, GRMZM2G328795	5.80E-27	NP_001183831 (958 aa)	ZIP4/ SPO22-LIKE	1	60	-	Chr:6*	105068634	105068693	+	60	5.80E-27	100.0	60	ND	LOC_Os01g66690	ND
TC296253	GRMZM2G417525	5.80E-27	NP_001168656 (559 aa)	Unknown protein	1	60	-	Chr:5	89002983	89003042	+	60	5.80E-27	100.0	60	Sb04g005185	LOC_Os02g08080	At4g31430
TC283173	GRMZM2G083138	5.80E-27	ND	**DNA repair protein RAD54-like**	1	60	-	Chr:5	209743778	209743837	+	60	5.80E-27	100.0	60	ND	LOC_Os02g52510	At3g19210
TC305717	GRMZM5G801875	5.80E-27	NP_001132241 (534 aa)	MATE efflux family protein	1	60	+	Chr:4	49918034	49918093	+	60	5.80E-27	100.0	60	Sb07g023840	LOC_Os08g44870	At4g00350

TC307556	GRMZM2G039650	1.90E-17	NP_001168634 (623 aa)	Double-stranded RNA binding motif protein	1	29	+	Chr:3*	177678135	177678163	+	29	1.90E-17	100.0	29	Sb03g040355	LOC_Os01g63820	ND
TC284771	AC231745.1_FG004	5.80E-27	ND	ND	1	60	+	Chr:9	18244374	18244433	+	60	5.80E-27	100.0	60	ND	ND	ND
TC294651	GRMZM5G842071	5.80E-27	NP_001105789 (641 aa)	Laccase 1	1	60	-	Chr:3	179116291	179116350	+	60	5.80E-27	100.0	60	Sb03g039970	LOC_Os01g63190	At5g01040 = LAC7
TC294126	GRMZM2G039978	1.10E-07	NP_001144895 (159 aa)	Integral membrane protein, putative	31	60	-	Chr:1	280979679	280979708	+	30	1.10E-07	100.0	30	Sb01g006950	LOC_Os03g55870	At1g49405
TC308574	GRMZM2G039577	3.40E-22	NP_001168388 (401 aa)	26S proteasome regulatory particle non-ATPase subunit10	5	56	+	Chr:9*	56878561	56878616	+	52	3.40E-22	98.2	56	Sb01g026350	Os03g0243300	At4g38630 = MBP1 = RPN10
TC305158	GRMZM2G159724	0.00018	NP_001152396 (593 aa)	NADP-dependent malic enzyme	4	30	-	Chr:3	201684823	201684853	+	27	0.00018	96.8	31	Sb09g005810	LOC_Os01g52500	At5g11670 = ATNADP-ME2
TC302695	GRMZM2G017388	7.90E-16	NP_001142348 (586 aa)	Cation/calcium exchanger 4	18	60	-	Chr:1	238565211	238565265	+	43	7.90E-16	94.6	55	Sb08g022240	LOC_Os10g30070, LOC_Os11g01580, LOC_Os03g40320	At1g54115 = CCX4
TC301530	GRMZM2G054115	5.80E-27	NP_001141015 (192 aa)	Alliinase family protein ; cysteine sulphoxide lyase	1	60	-	Chr:3	202718500	202718559	+	60	5.80E-27	100.0	60	Sb09g005070	LOC_Os01g52010, LOC_Os01g51980	At1g34060
CO533393	GRMZM2G173186	5.80E-27	ND	**DNA mismatch repair protein MutS4-like MSH4**	1	60	+	Chr:2	199667589	199667648	+	60	5.80E-27	100.0	60	Sb02g033470, Sb02g033480	LOC_Os07g30240	At4g17380 = AtMSH4
BM378145	GRMZM2G041418	5.80E-27	ACN29078 (557 aa)	Putative NADH dehydrogenase	1	60	+	Chr:7	157897423	157897482	+	60	5.80E-27	100.0	60	Sb02g036490	LOC_Os07g37730	At2g29990 = NAP2
TC303749	GRMZM2G020766	5.80E-27	ND	Pseudogene of a potassium transporter	1	60	-	Chr:7	172260743	172260802	+	60	5.80E-27	100.0	60	Sb02g042430	LOC_Os07g47350	At3g02050 = KUP3
TC295193	GRMZM2G536584, GRMZM2G441932	1.40E-24	ND	ND	5	60	+	Chr:10*	88675965	88676024	+	56	1.40E-24	98.3	60	ND	ND	ND
TC291009	GRMZM2G032562	5.80E-27	NP_001148633 (131 aa)	**SKP1-like protein 1B**	1	60	+	Chr:7	175642452	175642511	+	60	5.80E-27	100.0	60	Sb04g000330	LOC_Os09g36830	At5g42190 = ASK2
TC298798	GRMZM2G032003	5.80E-27	NP_001130742 (473 aa)	UTP-glucose-1-phosphate uridylyltransferase	1	60	-	Chr:7	146492461	146492520	+	60	5.80E-27	100.0	60	Sb04g001320	LOC_Os09g38030	At5g17310
TC294408	AC203841.3_FG009	5.80E-27	ACG29294 (346 aa)	CAB2	1	60	-	Chr:10	124231623	124231682	+	60	5.80E-27	100.0	60	Sb06g019590	LOC_Os05g13330	At1g16560
TC279550	GRMZM2G146206	5.80E-27	NP_001147215 (256 aa)	Triosephosphate isomerase, cytosolic	1	60	+	Chr:8	168749080	168749139	+	60	5.80E-27	100.0	60	Sb03g006130	LOC_Os01g62420	At3g55440 = TPI
DT652253	GRMZM2G050684	5.80E-27	NP_001149526 (422 aa)	CBS domain containing protein	1	60	-	Chr:1	257213276	257213335	+	60	5.80E-27	100.0	60	Sb01g012180	LOC_Os03g47120	At4g33700
TC310105	GRMZM2G366065	5.80E-27	NP_001168646 (421 aa)	Nodulin-like protein	1	60	-	Chr:2	31200911	31200970	+	60	5.80E-27	100.0	60	Sb06g021760	LOC_Os04g42420	At1g80530
TC312497	GRMZM2G052821	9.10E-26	NP_001146620 (421 aa)	**DNA photolyase ; UV REPAIR DEFECTIVE 3 (UVR3)**	3	60	-	Chr:5	110514186	110514243	+	58	9.10E-26	100.0	58	ND	LOC_Os02g10990	At3g15620 = At UVR3
TC314427	GRMZM2G125775	1.80E-12	NP_001106261 (204 aa)	AN17 ; zinc finger (AN1-like)-like protein	1	40	-	Chr:2*	178362550	178362589	+	40	1.80E-12	100.0	40	Sb02g023910	LOC_Os09g21710	At3g28210 = At SAP12

Highlighted items are genes previously reported to be associated with meiosis or meiosis-related processes; ND: not determined; *: multiple genome locations found.
