# Supplementary material for: Global transcriptome analysis of two ameiotic1 alleles in maize anthers: defining steps in meiotic entry and progression through prophase I
Source: BMC Plant Biol. 2011 Aug 26;11:120. doi: 10.1186/1471-2229-11-120 (PMC3180651; doi:10.1186/1471-2229-11-120)
Supplement: Additional file 8 — List of 37 PMC-enriched genes clustered with the Am1 transcript expression pattern as in Figure 6. Highlighted items are genes previously reported to be associated with meiosis or meiosis-related processes; ND: not determined. [file 1471-2229-11-120-S8.PDF]

Probe ID	Description
DQ663482	***Ameiotic1***
TC283097	CBS (cystathionine beta-synthase) domain containing protein
TC309808	Flavin-containing monooxygenase YUCCA-type
TC298200	ND
CO440202	ND
TC301734	SPX (SYG1/Pho81/XPR1) domain-containing protein / zinc finger (C3HC4-type RING finger) protein-related
TC282507	Transmembrane emp24 domain-containing protein 10 precursor
TC299943	Suppressor of Gene Silencing 3 (SGS3) homolog
TC297465	GRAS family transcription factor similar to SCARECROW-like 26
TC295587	MPS one binder kinase activator-like 1A; **cell cycle associated protein Mob1-like protein**
TC307549	ASC1-like protein 2 (Alternaria stem canker resistance-like protein 2); similar to At LAG1 LONGEVITY ASSURANCE HOMOLOG 3
CD436448	Unkown function
CF040072	Unknown protein
TC308341	Transferring glycosyl groups / trehalose-phosphatase
TC311848	Egg Apparatus-1 protein
TC280797	ND
CO441573	RNA recognition motif family protein
TC280985	Voltage-gated potassium channel beta subunit
TC285655	CID11
DT647408	Pumilio/Puf RNA-binding domain-containing protein-like
TC313063	Cp protein; Protein of unknown function, DUF538
TC314264	ND
TC297993	MADS-box transcription factor 26, similar to Agamous-like 12
TC310988	GDP-mannose 3,5-epimerase 2
TC279890	Mitochondrial processing peptidase alpha subunit
TC286746	Hydrolase, alpha/beta fold protein-like
TC304579	Early-responsive to dehydration protein
TC286055	ND
TC284035	ND
TC293287	Serine/threonine-protein kinase SAPK4
TC302095	EF hand (Calcium binding motif) family protein
TC292387	Glutamine amidotransferases class-II (GATase)
CF629011	Alternaria stem canker resistance (ASC)-like protein 2
TC282818	Domon-like ligand-binding domain protein
TC283544	ND
TC313084	Putative NADPH dehydrogenase
TC301530	Alliinase family protein; cysteine sulphoxide lyase
