# Supplementary material for: Global transcriptome analysis of two ameiotic1 alleles in maize anthers: defining steps in meiotic entry and progression through prophase I
Source: BMC Plant Biol. 2011 Aug 26;11:120. doi: 10.1186/1471-2229-11-120 (PMC3180651; doi:10.1186/1471-2229-11-120)
Supplement: Additional file 9 — Heat map and annotation of the six transcripts most similarly regulated with the Ameiotic1 gene. F = fertile; S = male sterile; ND = not determined. [file 1471-2229-11-120-S9.PDF]

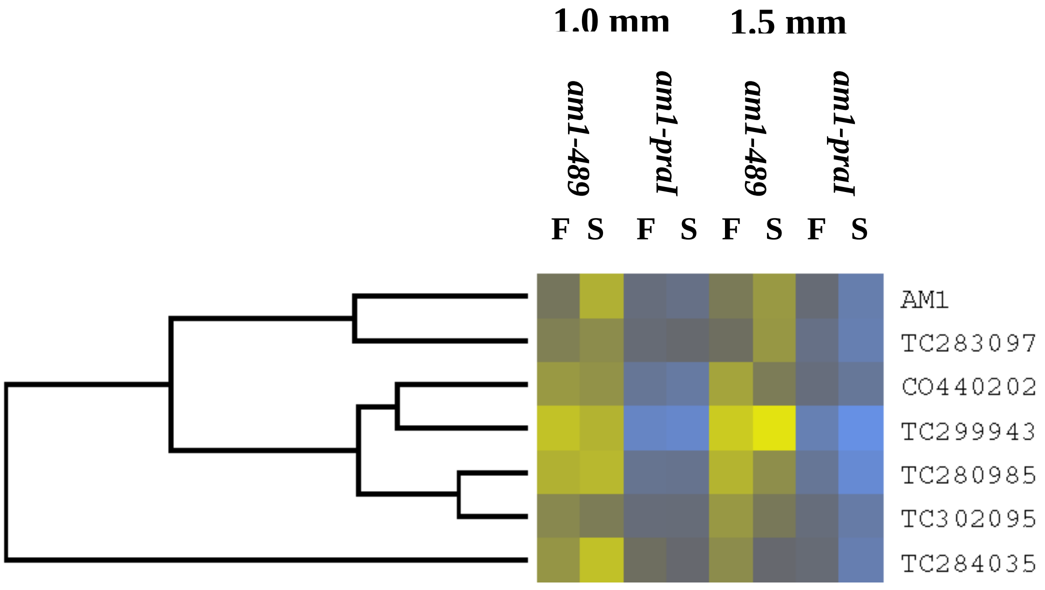

Probe ID	Sequence ID	Description
DQ663482	GRMZM5G883855	**Ameiotic1**
TC283097	GRMZM2G050684	CBS (cystathionine beta-synthase) domain containing protein
TC299943	GRMZM2G163514	Suppressor of gene silencing 3 (SGS3) homolog
TC280985	GRMZM2G356938	Voltage-gated potassium channel beta subunit
TC302095	GRMZM2G000397	EF hand (Calcium binding motif) family protein
CO440202	GRMZM2G083394	ND
TC284035	GRMZM5G890815	ND
