# Supplementary material for: Global transcriptome analysis of two ameiotic1 alleles in maize anthers: defining steps in meiotic entry and progression through prophase I
Source: BMC Plant Biol. 2011 Aug 26;11:120. doi: 10.1186/1471-2229-11-120 (PMC3180651; doi:10.1186/1471-2229-11-120)
Supplement: Additional file 10 — List of 45 PMC-enriched genes clustered with the Skp1B transcript expression pattern as in Figure 7. Highlighted items are genes previously reported to be associated with meiosis or meiosis-related processes. ND: not determined. [file 1471-2229-11-120-S10.PDF]

	Description
TC314580	Auxin-independent growth promoter-like protein
DT647788	Unknown protein
TC293449	SU1 isoamylase
TC292774	Unknown protein
TC298797	UTP-glucose-1-phosphate uridylyltransferase
TC315488	Plant calmodulin-binding domain
TC284111	Ras-related protein Rab-6A (microtubule-dependent transport pathways through the Golgi)
DR829208	Putative O-acetyltransferase
TC305157	NADP-dependent malic enzyme
TC310683	Ribulose-phosphate 3-epimerase, chloroplast precursor
TC309747	ND
TC313491	ND
TC296255	Putative RSZp22 splicing factor
TC283431	Unknown protein
TC288590	BAG domain containing protein (BCL-2-ASSOCIATED ATHANOGENE 5)
TC289753	Putative RNA-binding protein RNP-D precursor
TC301790	**Benzothiadiazole-induced somatic embryogenesis receptor kinase 1 (SERK1)**
TC285165	ND
TC306072	ND
TC283852	Potential U2 snRNA pseudouridine synthase-like
DR795221	MSP (major sperm protein) domain, VAMP/SYNAPTOBREVIN-ASSOCIATED PROTEIN 27-2
AW231811	ND
TC283691	ND
TC307363	Probable potassium transporter 14
TC310688	Ribulose-phosphate 3-epimerase
TC314676	Unknown protein
CD995221	ND
TC287319	Chorismate mutase type II
TC311526	Inositol-tetrakisphosphate 1-kinase 1
DR906542	Unknown protein
TC307982	Unknown protein
TC289354	Similar to NC domain-containing protein-related
TC306026	O-acetyltransferase family protein
TC284639	Unknown protein
TC294269	Acid phosphatase/vanadium-dependent haloperoxidase related
TC290471	Unknown protein
TC295705	Acetyl-CoA synthetase-like protein
TC314126	Unknown protein
TC296253	Unknown protein
TC283173	**DNA repair protein RAD54-like**
TC305717	Unknown protein
TC307556	Unknown protein
TC308574	Unknown protein
TC305158	ND
TC291009	**SKP1-like protein 1B**
