# Supplementary material for: Global transcriptome analysis of two ameiotic1 alleles in maize anthers: defining steps in meiotic entry and progression through prophase I
Source: BMC Plant Biol. 2011 Aug 26;11:120. doi: 10.1186/1471-2229-11-120 (PMC3180651; doi:10.1186/1471-2229-11-120)
Supplement: Additional file 11 — Primer sequences and RT product sizes of all genes surveyed in qRT validation experiments. [file 1471-2229-11-120-S11.PDF]

Gene	Identifier	Primer Sequence (5'→3')	Product Size (bp)
Am1	DQ663482	CCTCTCCAGCTCCTTCCTTT<br>GCTTCACTTGGAGCCAGTTC	182
Afd1	AY788900	AGGTTACTCAGCTGGAACGG<br>TCGCTCCCTTTCTTTCACC	218
Dmc1	TC313913	TCAGGTGGAATCATGGACG<br>GGAAACATGTAATCCGGGG	237
Rad51A	TC301172	TAAAACGATGCCTGTTGCC<br>CACCACAATGCTACCTGCC	203
Rad51A2	TC302198	TACAGGCTTCCAGAATGCG<br>ACGCCCACCATAGGGC	150
Rad54	TC283173	GCAAGCAATTTGGTGATGG<br>AAACGGAATAGCAGCCACC	163
RPA70	TC313965	TCTGCTACACAGCTTGCCC<br>TGTCACAGAAGTTGTTCCCG	175
Skp1A	BM660128	CTCTGACTTCATCGCCACC<br>TCGTTCCCAATCATCTTGC	171
Skp1B	TC291009	GGAGATACGCGAGACATTCG<br>CACTTCATCTCTGGCCTGG	155
Spo11-1	TC306885	AAGGAAATGGAATTTGCGG<br>CAAACTTGTTGCGTGTCCC	243
Zyp1	TC283445	AAGATCCCGTGCACATCC<br>TAGGATTGACCCAACACGG	177
